# Topoisomerase I Inhibition in ETV4‐overexpressed Non‐Small Cell Lung Cancer Promotes Replication and Transcription Mediated R‐Loop Accumulation and DNA Damage

**DOI:** 10.1002/advs.202409307

**Published:** 2025-06-23

**Authors:** Jiaxi Zhang, Yan Wang, Shanhu Cao, Shelly M. Xie, Bei Liu, Yimeng Li, Yuqi Hou, Xue Meng, Mingzhu Ruan, Di Bu, Jia Kang, Ruxin Li, Lei Lou, Juan Wang, Lingxiao Xing

**Affiliations:** ^1^ Department of Pathology Hebei Medical University Center of Metabolic Diseases and Cancer Research Institute of Medical and Health Science Hebei Medical University Shijiazhuang 050017 P. R. China; ^2^ Department of Cardiology The First Hospital of Hebei Medical University Hebei Key Laboratory of Heart and Metabolism Department of Biochemistry and Molecular Biology Hebei Medical University Shijiazhuang 050017 P. R. China; ^3^ Department of Pathology Second Hospital of Hebei Medical University Shijiazhuang 050017 P. R. China

**Keywords:** DNA damage, DNA replication, ETV4, NSCLC, R‐loop, TOP1 inhibitor

## Abstract

Coordinating transcription and replication via transcription factors (TFs) is a conserved mechanism in higher eukaryotes. The role of TFs in regulating these processes in cancers remains unclear. Here, it is shown that oncogenetic ETS transcription factor ETV4 controls DNA replication through both transcriptional and non‐transcriptional mechanisms in non‐small cell lung cancer (NSCLC). ETV4 localizes to specific DNA replication origins and interacts with the origin recognition complex subunits ORC1 and ORC6 during the G1/S phase, facilitating origin formation. Using quantitative in situ analysis of protein interactions at DNA replication forks (SIRF) assays, it is shown that ETV4 transiently localizes to replication forks in the S phase. It interacts with replicative helicase MCM2 N‐terminal, histone H3, and histone‐chaperone FACT and is involved in histone processing during replication. Additionally, ETV4 transcriptionally regulates key replisome genes MCM2, MCM4, MCM5, MCM10, and ORC1, influencing their expression and recruitment to chromatin. Due to its binding at the origin‐promoter locus like the MCM4 gene, ETV4 overexpression increases R‐loop formation, DNA damage, and cell death under external replication stress induced by topoisomerase I (TOP1) inhibitor. These findings highlight the dual role of ETV4 in replication and transcription and suggest that targeting TOP1 could be a synthetic‐lethal approach in ETV4‐overexpressed lung cancer.

## Graphical abstract

The oncogenic ETS factor ETV4 exerts a pleiotropic control over DNA replication both in a transcription‐dependent and ‐independent fashion in NSCLC cells. High‐ETV4 expression leads to R‐loop formation and DNA damage in response to TOP1 inhibition. As targeting ETV4 directly is challenging, our findings present an exciting alternative strategy to exploit TOP1 as novel targets in ETV4‐dysregulated lung cancers.

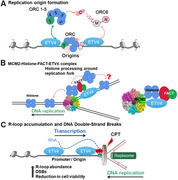

## 1 Introduction

Eukaryotic DNA replication is a highly organized process that initiates at thousands of specific sites, known as replication origins, across the entire genome to ensure complete replication. DNA replication is achieved by assembling a large, multi‐protein complex called the “replisome” at each potential origin before DNA synthesis.^[^ ^1^ ^]^ In the G1 phase, DNA replication origins are licensed by the sequential loading of the origin recognition complex (ORC1‐6), Cdc6, Cdt1, and two MCM2‐7 ring hexamers onto the origin DNA, forming the pre‐replicative complex (pre‐RC). In the S phase, the stable CMG (Cdc45–MCMs–GINS) replicative helicase complex assembles on these licensed origins through Dbf4‐dependent kinase (DDK) and cyclin‐dependent kinase (CDK), forming the pre‐initiation complex. Formation of a complete replisome requires recruiting additional firing factors, such as MCM10, RPA, and replicative polymerases, and initiating DNA synthesis at the leading and lagging strands (origin firing).^[^ ^2^, ^3^ ^]^ Origin distribution and firing define the timing and genomic regions replicated in the cell. While bulk DNA replication occurs during the S phase, recent findings suggest that DNA synthesis persists after the S phase, into G2, mitosis, and the subsequent G1 phase to ensure the genome is fully duplicated.^[^ ^4^, ^5^ ^]^

In higher eukaryotes, replication origin selection lacks a defined consensus sequence. This absence is attributed to the large genome sizes, large replication initiation zones, movement of MCM2‐7 double‐hexamer, and technical limitations.^[^ ^6^ ^]^ Transcription is known to be profoundly correlated with replication origin placement and efficiency.^[^ ^7^, ^8^ ^]^ Transcription could induce changes in chromatin structure and cooperate with CTCF and cohesin‐based loop extrusion to position high‐efficiency replication initiation zones replicating in the early S phase.^[^ ^9^, ^10^ ^]^ Replication origins in higher eukaryotes are frequently found in close proximity to promoters of actively transcribing genes, suggesting a conserved mechanism that utilizes transcription factors (TFs) to coordinate replication and transcription processes flexibly.^[^ ^11^, ^12^ ^]^ Several reports have suggested a direct role of TFs in replication licensing at mammalian origins. For example, c‐Myc can interact with pre‐RC proteins, such as MCMs, ORC2, and CDC6,^[^ ^13^ ^]^ binding transiently to Lamin B2 origin in the G1 phase, leading to histone hyperacetylation and pre‐RC loading on the origin.^[^ ^14^ ^]^ Gain‐of‐function mutant p53 R273H interacts with replicating DNA, recruits MCMs and PARP1 chromatin interaction, and increases synergistic PARPi plus temozolomide treatment sensitivity in breast cancer.^[^ ^15^ ^]^ ETS transcription factor ETS2 is recruited to the MCM4 origin in the G1 phase via active histone acetylation.^[^ ^12^ ^]^ Despite these findings, the molecular mechanism by which TFs regulate origin selection and DNA replication remains poorly understood.

DNA replication and nucleosome assembly are tightly linked to maintain genome integrity and function. Nucleosomes ahead of the replication fork act as barriers for the replication machinery and must be disassembled to allow the fork to pass. New nucleosomes must be reformed to restore the chromatin state.^[^ ^16^ ^]^ One of the critical factors linking these processes is MCM2, which hijacks interaction sites used by nucleosomal DNA to chaperone both new and old histones H3‐H4 at replication forks through the histone‐binding domain (HBD) in its N‐terminal tail, thus endows the replicative helicase with ideal properties for recycling parental histones with their posttranslational modifications (PTMs) during replication. Impaired MCM2 histone‐binding hampers efficient S phase progression, cell growth, and the transfer of parental histones.^[^ ^17^, ^18^ ^]^ Also, MCM2 binds histones cooperatively with the histone chaperone, such as Asf1 and the FACT (facilitates chromatin transcription, consisting of subunits SUPT16H and SSRP1) complex.^[^ ^19^, ^20^ ^]^ FACT is a conserved histone chaperone that can both destabilize and reassemble nucleosomes.^[^ ^21^ ^]^ Each subunit comprises multiple histone‐binding modules connected by unstructured linkers.^[^ ^22^ ^]^ A recent cryo‐EM structure revealed that the architecture of the FACT‐nucleosome complex resembles a unicycle to maintain chromatin integrity during polymerase passage.^[^ ^23^ ^]^ Therefore, the unique role of the MCM2‐FACT association is critical for the tight coupling between histone processing and DNA unwinding during replication. It has been reported that MYC colocalizes with some replication origins and interacts with the MCMs, including MCM2.^[^ ^13^ ^]^ However, whether the TFs‐MCM2 interaction at the replication sites confers specificity in histones re‐associating remains poorly defined.

The ETS family of oncogenic TFs is emerging as crucial mediators of tumorigenesis in solid tumors.^[^ ^24^ ^]^ We have previously shown that ETV4 is the preponderant ETS factor associated with glycolytic metabolism, tumor progression, and worse prognosis in NSCLC.^[^ ^25^, ^26^ ^]^ In a stabilized ETV4‐driven murine prostate cancer model, it was demonstrated that ETV4 overexpression alone, when expressed at high dosage, can initiate tumorigenesis and cooperate with TP53 loss for tumor progression.^[^ ^27^ ^]^ ETV4 increases CXCL1 and CXCL8 secretion to recruit tumor‐associated neutrophil infiltration and promotes lymphangiogenesis and lymphatic metastasis, identifying ETV4 as a therapeutic target in bladder cancer.^[^ ^28^ ^]^ In small‐cell lung cancer, ETV4 and ETV5 are critical regulators of clonogenic regrowth from a diapause‐like state in drug‐tolerant persisters (DTPs) cells following cisplatin and etoposide challenge.^[^ ^29^ ^]^ Furthermore, ETV4 is related to the resistance against kinase inhibitors (e.g., MAPKi, EGFR‐TKI) and immune therapies (e.g., Lenalidomide, PD‐L1) in multiple cancers.^[^ ^30^, ^31^, ^32^, ^33^ ^]^ ETV4 is among the 568 cancer‐driven genes from 28,076 tumor samples of 66 cancer types.^[^ ^34^ ^]^ Beyond the critical oncogenetic function in multiple cancers, ETV4 acts as an on/off switch with ultrasensitive dependence on cell crowding that links mechanical microenvironments and gene expression to drive spatiotemporal lineage specification in human embryonic stem cells. The dynamic ETV4 expression captured by mathematical modeling might involve the mechanisms underlying tumor progression and suppression.^[^ ^35^ ^]^ Therefore, new insights into the molecular mechanisms hijacked by ETV4 will pave the way for novel therapeutic strategies.

We report that ETV4 is located at the DNA replication origins and contributes to origin formation by interacting with ORC subunits. ETV4 binds with the N‐terminus of MCM2 and the FACT complex, localizes in proximity to newly replicated DNA, and might participate in histone processing during replication. In addition, ETV4 transcriptionally controls the essential replisome gene expression and affects their recruitment to chromatin. Because ETV4 binds to the origin‐promoter locus like the MCM4 gene, we further show that ETV4 level is correlated with R‐loop accumulation‐induced DNA damage and reduction in cellular viability following TOP1 inhibitor‐camptothecin (CPT) treatment in NSCLC cells. Recently, several CPT analogs and TOP1 antibody–drug conjugates (ADCs) have been FDA‐approved for treating solid tumors.^[^ ^36^ ^]^ Our work suggests that inhibition of TOP1 might be a promising target for ETV4‐dysregulated lung cancer.

## 2 Results

### 2.1 ETV4 is Located at the DNA Replication Origins and Contributes to Origin Formation by Interacting with ORC Subunits in NSCLC Cells

**Figure 1:**
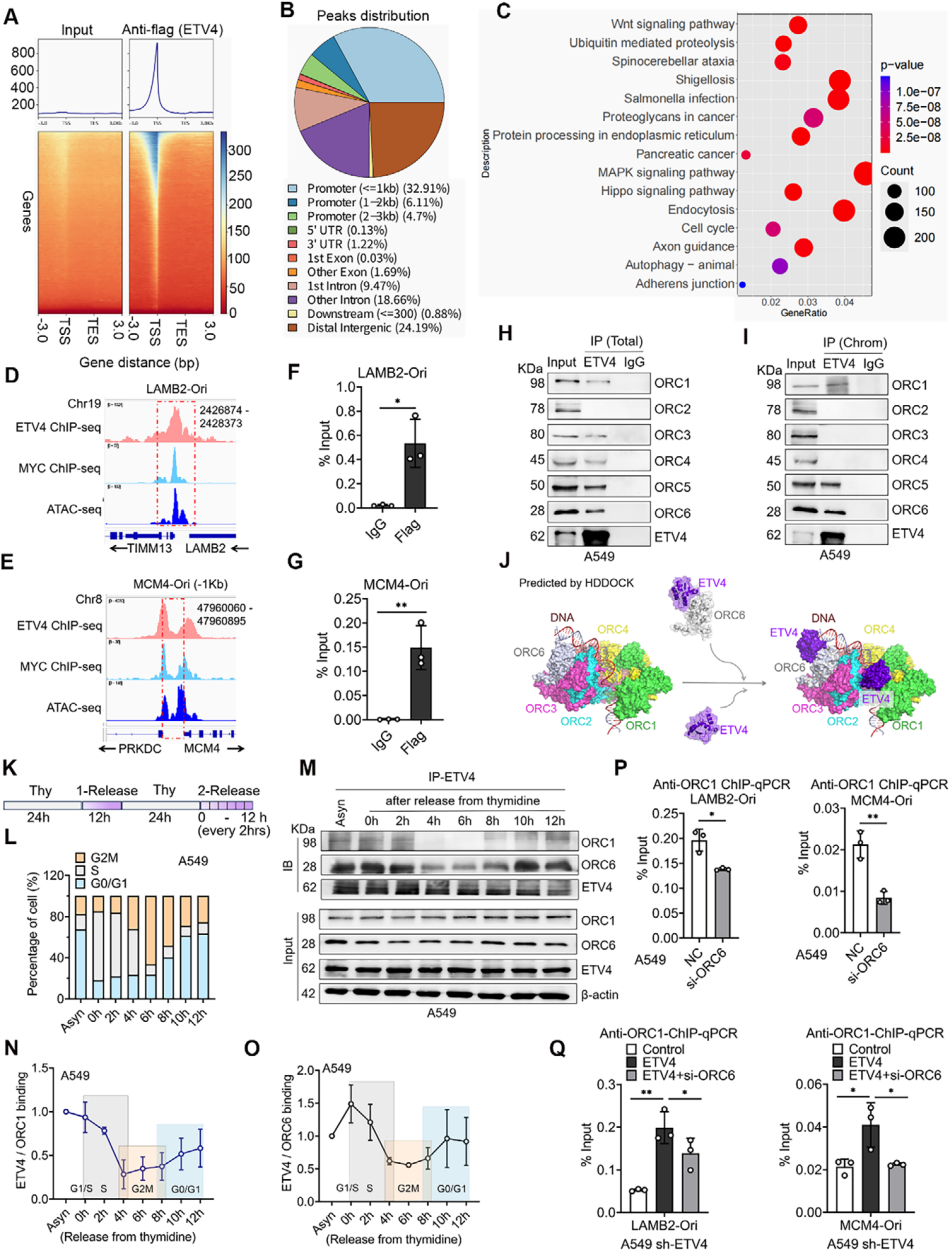
ETV4 is located at the origins of LAMB2 and MCM4 DNA replication and plays a role in ORC loading at the origins of NSCLC cells. A) Gene body enrichment heat map of anti‐Flag ChIP‐seq signals in A549‐shETV4 cells transfected with Flag‐ETV4 plasmids. B) Pie chart demonstrating the percentage of ETV4 peak distribution. C) Functional analysis showing the top KEGG pathways significantly associated with genes of ETV4 binding by ChIP‐seq assay. D,E) IGV tracks showing the co‐occupancy of ETV4 and c‐MYC with the chromatin accessibility at the LAMB2 origin and MCM4 origin (‐1Kb) regions from our ETV4 ChIP‐seq, MYC ChIP‐seq (ENCSR537FDJ), and ATAC‐seq (ENCSR220ASC) from A549 cells. F,G) ChIP‐qPCR assay showing the binding of ETV4 at LAMB2 origin and MCM4 origin regions in A549‐shETV4 cells transfected with Flag‐ETV4 plasmids (mean ± SD, *n* = 3; two‐tailed unpaired *t*‐test). ^*^ *p* < 0.05, ^**^ *p* < 0.01. H,I) IP of ETV4 and Immunoblots against ORC1‐6 subunits from whole‐cell proteins or chromatin‐bound (Chrom.) proteins of A549 cells. J) Predicting model of the structural arrangement of ETV4 and ORC by superimposing the structure of ETV4 (PDB code 4co8) with the ORC complex (PDB code 7mca). dsDNA, double‐strand DNA. K,L) Schematic of the protocol for cells synchronized and released using a double thymidine block. The cell cycle distribution was detected by Flow cytometry. M) IP of ETV4 and Immunoblots against ORC1 and ORC6 from thymidine block and released synchronized A549 cells. N,O) Quantification of ETV4‐ORC1 or ORC6 binding in M. P) ChIP‐qPCR assay showing the binding of ORC1 at LAMB2 and MCM4 origins in A549 cells transfected with ORC6 siRNA or NC siRNA. (mean ± SD, *n* = 3; two‐tailed unpaired *t*‐test) ^*^ *p* < 0.05, ^**^ *p* < 0.01. Q) ChIP‐qPCR assay showing the binding of ORC1 at LAMB2 and MCM4 origins in A549‐shETV4 cells transfected with ETV4 plasmids or combined with ORC6 siRNA, respectively. (mean ± SD, *n* = 3; one‐way ANOVA followed by Tukey's multiple comparisons test). ^*^ *p* < 0.05, ^**^ *p* < 0.01.

To determine the genome‐wide chromatin binding of ETV4, we performed Chromatin‐IP‐sequence (ChIP‐seq) studies in A549‐shETV4 cells after re‐transfected with Flag‐ETV4 using a Flag antibody. In total, 54,389 ETV4 binding peaks representing 25,708 genes were identified (FC > 2), and 43.71% of peaks were enriched in the promoter regions. The top enrich‐KEGG pathway included the WNT, MAPK, cell cycle, autophagy signaling, etc (**Figure** **1**A–C). A cluster of transcription factors, including c‐MYC, binds to the well‐known LAMB2‐origin (Chr19:2426874‐2428373).^[^ ^8^, ^14^ ^]^ However, whether ETV4 binds with human replication origins is unknown. Interestingly, we found that ETV4 is located at the open chromatin regions of LAMB2‐origin by aligning the data obtained from our ETV4 ChIP‐seq with that of MYC ChIP‐seq (ENCSR537FDJ) and ATAC‐seq (ENCSR220ASC) for A549 cells using Integrative Genomics Viewer (IGV) (Figure 1D). In addition, the ‐1kb promoter region of MCM4 has been confirmed to be the region of prominent origin (Chr8: 47960060–47960895).^[^ ^12^, ^37^ ^]^ IGV tracks showed that ETV4 and c‐MYC were both enriched in MCM4‐origin (Figure 1E), which was further confirmed by ChIP‐qPCR experiments (Figure 1F,G). Moreover, we provided evidence that ETV4 is located at some other human replication initiation loci such as TOP1, JUNB, HPRT1, CTCF, and two intergenic replication initiation sites (CRPCCP2‐MST1P2 and PIK3CB‐Linc01391 origins).^[^ ^38^, ^39^ ^]^ using ChIP‐seq profiles and ChIP‐qPCR assays (Figure S1A–E, Supporting Information).

To further explore the binding of ETV4 at the origin location, we performed an Immunoprecipitation (IP) assay to see if ETV4 could bind with ORC subunits. H1299 and A549 cells showed relatively higher ETV4 expression, while H358 cells had a lower level of ETV4 protein (Figure S2A, Supporting Information). IP results showed that ETV4 interacted with ORC1‐6 subunits to a various extent except ORC2 from whole‐cell proteins, while the interaction was obviously detected between ETV4 and ORC1, ORC5, and ORC6 from chromatin‐bound (Chrom.) proteins in A549 (Figure 1H,I) and H1299 cells (Figure S2B, Supporting Information). Furthermore, a model for the structural arrangement of ETV4 with ORC at dsDNA was predicated by superimposing the structure of ETV4‐DBD (PDB code 4co8) with ORC complex (PDB code 7mca), further supporting the origins‐binding of ETV4, especially with ORC1 and ORC6 (Figure 1J). To explore ETV4 and ORC1/6 associations across the cell cycle, cells were synchronized into the G1/S phase using a double thymidine block, and following release, cell extracts were used for the IP test. Flow cytometry confirmed the cell cycle distribution as expected. The binding of ETV4 with ORC1 and ORC6 in G1/S was decreased in G2‐M phases, while it was increased during G1 phases (Figure 1K–O; Figure S2C–F, Supporting Information). To define the domain(s) of ETV4 required for the interaction with ORC1 and ORC6 subunit, the Flag‐tagged full‐length (FL, 1–484aa) or truncated ETV4 (ETV4‐ΔC, ‐ΔNID, ‐ΔN, and ‐ΔDBD) vectors were transfected into HEK293T, and cell extracts were used for IP. Compared with the binding of ETV4‐FL, N‐terminal deletion (Δ1‐164aa) bound more weakly to ORC1 and ORC6 proteins (Figure S3A–D, Supporting Information). To determine if ETV4 N‐terminal or DBD might affect the recruitment of ETV4 itself to DNA replication origins, we performed ChIP‐qPCR assays in A549 sh‐ETV4 cells. Anti‐Flag ChIP‐qPCR results showed that DBD deletion decreased the binding of ETV4 at the origins especially those located in the promoter region, such as MCM4, TOP1, and CTCF. In contrast, N‐terminal deletion significantly decreased ETV4 binding at all origins (Figure S4A–H, Supporting Information). Moreover, ETV4‐ΔN and ETV4‐ΔDBD inhibited cell EdU incorporation compared with the ETV4‐FL transfection (Figure S4I,J, Supporting Information), suggesting that the activity of ETV4‐linked DNA replication is dependent on both of the N‐terminal and DBD domains through different mechanisms. All these data indicate that ETV4 binds at specific replication initiation sites and interacts with ORC subunits ORC1 and ORC6 mainly through its N‐terminal.

Human ORC1‐5 and ORC6 can bind to DNA independently.^[^ ^40^ ^]^ ORC6 directly binds to the ORC3 subunit to form the functional six‐subunit ORC complex and qualifies as a regulatory subunit of ORC that plays roles in targeting, positioning, and assembling the functional ORC at the origins.^[^ ^41^, ^42^ ^]^ ChIP‐qPCR assays indicated that loss of ORC6 caused a reduction in ORC1‐5 (represented by ORC1) enrichment at LAMB2 and MCM4 origins, without affecting ORC1 protein expression in A549 and H1299 cells (Figure 1P; Figure S5A–C, Supporting Information). In addition, si‐ETV4 led to a decrease in the recruitment of ORC6 at both origins (Figure S5D–G, Supporting Information). Next, we explored the role of ETV4‐ORC6 on ORC1 loading at origins. ChIP‐qPCR results indicated that ORC6 silencing partially reversed the effects of ETV4 overexpression‐induced origin‐associated ORC1 levels in A549 sh‐ETV4 cells (Figure 1Q). Because the ETV4 N‐terminal is much more essential for the binding with ORC6, we next determined if ETV4 N‐terminal deletion might affect the ORC recruitment to the origins. Compared with the ETV4‐FL transfection, the ETV4‐ΔN construct significantly decreased the ORC6 and ORC1 levels at LAMB2 and MCM4 origins (Figure S5H–L, Supporting Information). Moreover, depleting ORC6 using specific siRNA significantly inhibited cell proliferation, EdU incorporation, and S phase progression in A549 and H1299 cells. Depletion of ORC6 reversed the effects of ETV4 on cell proliferation and EdU incorporation of H358‐ETV4 cells (Figure S6A–I, Supporting Information). These results indicate that origin‐associated ETV4 may function as ORC‐interacting protein and contribute to the replication origin formation in NSCLC cells.

### 2.2 ETV4 Binds with the Subunits of the DNA Replicative Helicase MCM2‐7 Complex and MCM2 N‐Terminal Region in NSCLC Cells

**Figure 2:**
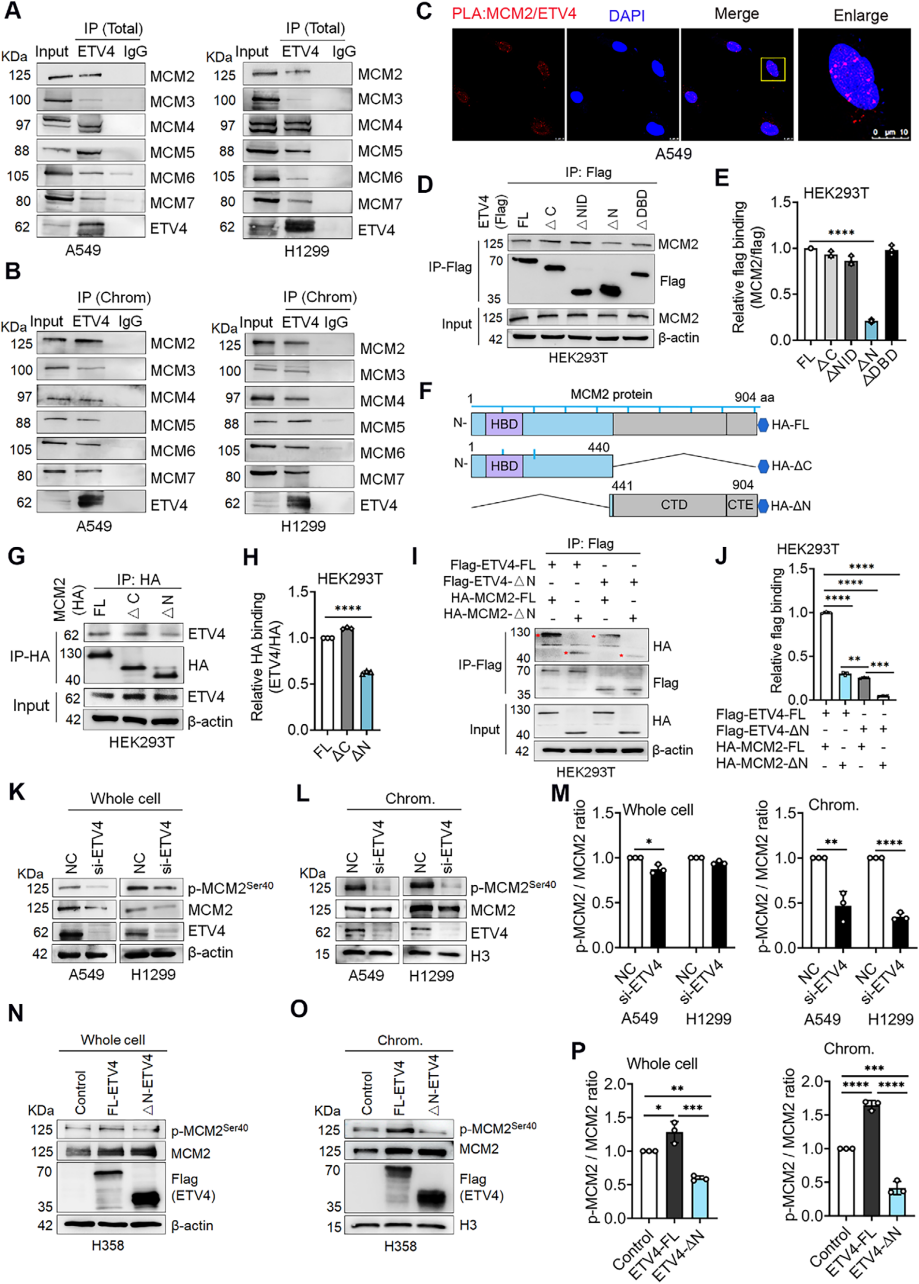
ETV4 binds with the subunits of the DNA replicative helicase MCM2‐7 complex and MCM2 N‐terminal region in NSCLC cells. A,B) IP of ETV4 and Immunoblots against MCM2‐7 protein from whole‐cell proteins or chromatin‐bound (Chrom.) proteins of A549 and H1299 cells. C) Analysis of ETV4/MCM2 association (red) by in situ proximity ligation assay (PLA) in A549 cells. DNA was counterstained with DAPI (blue). D,E) HEK293T cells were transfected with the indicated Flag‐ETV4 vectors, and the lysates were subjected to anti‐Flag immunoprecipitation followed by immunoblot analysis. Quantification of D (*n* = 3) presented as mean ± SD. One‐way ANOVA, ^****^ *p* < 0.0001. (F) Schematic representation of the C‐terminally HA‐tagged MCM2 deletion constructs. G,H) HEK293T cells were transfected as indicated, and lysates were subjected to anti‐HA immunoprecipitation followed by immunoblot analysis. Quantification of G (*n* = 3) presented as mean ± SD. Two‐tailed unpaired *t*‐test, ^****^ *p* < 0.0001. I,J) The interaction between ETV4 N‐terminal deletion (ETV4‐ΔN) and MCM2 N‐terminal deletion (MCM2‐ΔN) was tested using the Co‐IP assay. Quantification of I (*n* = 3) presented as mean ± SD. *P*‐values were based on the Brown‐Forsythe ANOVA test and Dunnett's T3 multiple comparisons test. ^**^ *p* < 0.01, ^***^ *p* < 0.001, ^****^ *p* < 0.0001. K–M) The expression of p‐MCM2^Ser40^ and MCM2 protein in A549 and H1299 ETV4‐knockdown cells from whole cell lysis and Chromatin‐bound fractions, respectively. Quantification of the ratio of p‐MCM2^Ser40^/MCM2 in K and L. (mean ± SD, *n* = 3; two‐tailed unpaired *t*‐test) ^*^ *p* < 0.05, ^**^ *p* < 0.01, ^****^ *p* < 0.0001. N–P) H358 cells were transfected with the full‐length Flag‐tagged ETV4 protein or ETV4‐ΔN plasmids, and the expression of p‐MCM2^Ser40^ and MCM2 protein was detected from whole cell lysis and Chromatin‐bound fractions using Western blot assay. Quantification of the ratio of p‐MCM2^Ser40^/MCM2 in N and O. (mean ± SD, *n* = 3; One‐way ANOVA followed by Tukey's multiple comparisons test). ^*^ *p* < 0.05, ^**^ *p* < 0.01, ^***^ *p* < 0.001, ****p < 0.0001.

In eukaryotes, the MCM2‐7 replicative helicase motor is deposited onto DNA by the ORC and co‐loader proteins to license replication origins.^[^ ^43^ ^]^ We performed co‐IP assays from whole‐cell and chromatin‐bound proteins to determine whether ETV4 might interact with the MCM2‐7 complex. IP‐ETV4 demonstrated the interaction between ETV4 and MCM2, MCM3, MCM4, MCM5, MCM6, and MCM7 in whole‐cell and chromatin‐bound proteins of A549 and H1299 cells (**Figure** **2**A,B). In addition, Immunoprecipitation of ETV4 demonstrated that ETV4 interacted with MCMs obviously during S phase synchronized cells and persisted after the S phase (Figure S7, Supporting Information). Because the MCM2 helicase subunit is required for MCM2‐7 histone‐chaperone function and normal cell proliferation,^[^ ^17^ ^]^ we next determined to extensively explore the association of ETV4 with MCM2 protein. Proximity ligation assay (PLA) signals confirmed the in situ interaction of ETV4 and MCM2 in the nucleus of A549 cells (Figure 2C). To define the domain(s) required for their interaction, the Flag‐tagged ETV4‐FL or truncated ETV4 constructs were transfected into HEK293T, and cell extracts were used for IP. Figure 2D,E showed that the ETV4‐full protein and deletion mutants could coimmunoprecipitate with MCM2, while the ETV4‐ΔN variant bound more weakly. In addition, the HA‐tagged MCM2‐FL and deletion constructs MCM2‐ΔN (Δ1‐440) and MCM2‐ΔC (Δ441‐904) were tested in a similar manner. It showed that the N‐terminal deletion of MCM2 markedly decreased the interaction with ETV4 (Figure 2F–H). Co‐transfection of ETV4‐ΔN and MCM2‐ΔN vectors further demonstrated the crucial binding between the ETV4 N‐terminal and MCM2 N‐terminal region (Figure 2I,J). Multiple phosphorylation sites of MCM2, MCM4, and MCM6 by specific kinases is essential in initiating DNA replication.^[^ ^44^ ^]^ CDC7 kinase‐dependent phosphorylation of MCM2 at Ser‐40, Ser‐53, and partially Ser‐108 is relevant for MCM2 function during the S phase of the cell cycle.^[^ ^45^ ^]^ Consistently, ETV4 knockdown inhibited both total and chromatin‐bound MCM2 and p‐MCM2^Ser40^ levels and a similar change of chromatin‐bound p‐MCM2^Ser40^/MCM2 ratio in A549 and H1299 cells (Figure 2K–M). Furthermore, ETV4‐N terminal deletion decreased the p‐MCM2^Ser40^/MCM2 ratio from the overall and chromatin‐bound proteins compared to the ETV4‐FL transfection in H358 cells (Figure 2N–P). Together, these findings show that ETV4 interacts with the MCM2‐7 complex, especially with the MCM2 N‐terminal region.

### 2.3 ETV4‐MCM2 Interacts with Histone H3 and Histone Chaperone FACT Subunits SUPT16H, SSRP1 in NSCLC Cells

**Figure 3:**
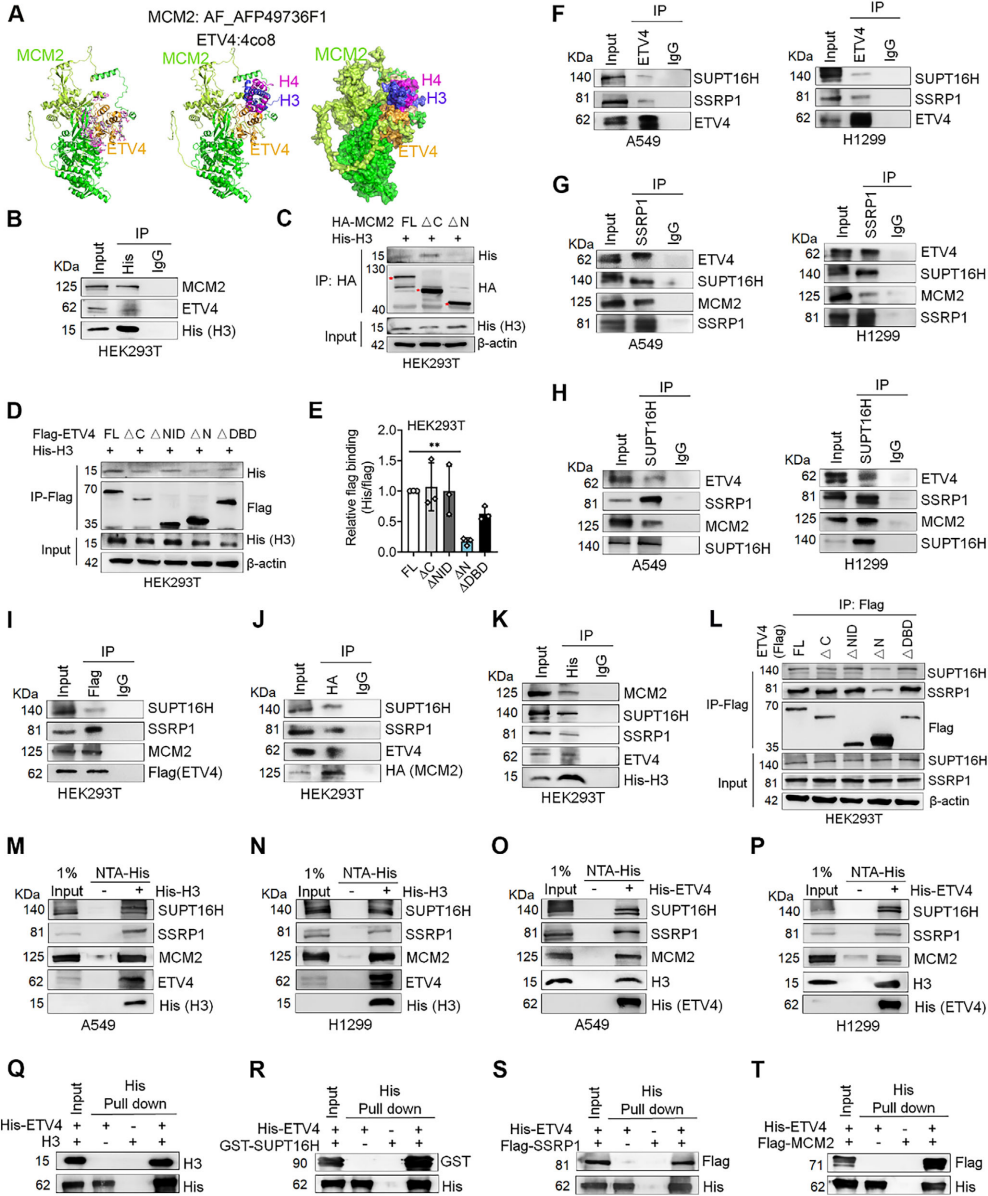
ETV4‐MCM2 interacts with histone H3 and histone chaperone FACT subunits SUPT16H and SSRP1. A) Predicting model of the structural arrangement of the MCM2‐H3‐H4‐ETV4 complex obtained by superimposing the structure of H3‐H4 dimer with MCM2 (PDB code AF‐AFP49736F1) and ETV4 (PDB code 4co8). B) HEK293T cells were transfected with His‐histone H3, and lysates subjected to anti‐His‐IP followed by immunoblot against MCM2 and ETV4. C) Co‐IP assay showing the interaction between HA‐MCM2 (‐FL, ‐ΔC, and ‐ΔN) and His‐histone H3 from HEK293T cells. D,E) Co‐IP assay showing the interaction between Flag‐ETV4 (‐FL, ‐ΔC, ‐ΔNID, ‐ΔN, and ‐ΔDBD) and His‐histone H3 from HEK293T cells. Quantification of D (*n* = 3) presented as mean ± SD. One‐way ANOVA, ^**^ *p* < 0.01. F) IP of ETV4 and immunoblot against SUPT16H and SSRP1 in A549 and H1299 cells. G) IP of SSRP1 and immunoblot against ETV4, SUPT16H, and MCM2 in A549 and H1299 cells. H) IP of SUPT16H and immunoblot against ETV4, SSRP1, and MCM2 in A549 and H1299 cells. I) The interactions of Flag‐ETV4 with SUPT16H, SSRP1, and MCM2 from HEK293T cells. J) The interactions of HA‐MCM2 with SUPT16H, SSRP1, and ETV4 from HEK293T cells. K) The interactions of His‐histone H3 with SSRP1, SUPT16H, MCM2, and ETV4. L) Co‐IP assay showing the interaction between Flag‐ETV4 (‐FL, ‐ΔC, ‐ΔNID, ‐ΔN, and ‐ΔDBD) and SUPT16H/SSRP1 from HEK293T cells. M,N) His‐histone H3 interacts with ETV4, MCM2, SUPT16H, and SSRP1 protein from the lysates of A549 and H1299 cells by in vitro His‐tag pull‐down assays. O,P) His‐ETV4 interacts with MCM2, SUPT16H, SSRP1, and histone H3 protein from the lysates of A549 and H1299 cells by in vitro His‐tag pull‐down assays. (Q–T) His pull‐down assays showed direct binding of histone H3 (Q), GST‐tag SUPT16H (1‐510aa) (R), Flag‐tag SSRP1 (S), or Flag‐tag MCM2 (1‐440aa) (T) to His‐ETV4.

MCM2 has histone‐chaperone activity via its N‐terminal HBD (43‐160aa) and collaborates with histone chaperones (FACT or ASF1) to mediate redeposition of new and old histones at replication forks.^[^ ^17^, ^20^ ^]^ We want to know if the ETV4‐MCM2 N‐terminal association might be involved in histone management during DNA replication. To this end, a model for the structural arrangement of the MCM2‐H3/H4‐ETV4 complex was predicated by superimposing the structure of the H3/H4 dimer with MCM2 (PDB code AF‐AFP49736F1) and ETV4 (PDB code 4co8) (**Figure** **3**A). IP with His (histone H3) showed the interactions of histone H3 with MCM2 and ETV4 proteins (Figure 3B). As expected, MCM2‐ΔN (Δ1‐440) almost lost the interaction with histone H3 (Figure 3C). Furthermore, ETV4‐ΔN or ΔDBD decreased its association with H3, especially the N‐terminal deletion (Figure 3D,E). In addition, reciprocal‐IP results demonstrated the interactions between endogenous ETV4 with SUPT16H, SSRP1, and MCM2 protein in A549 and H1299 cells (Figure 3F–H). IP with a Flag (ETV4), HA (MCM2), or His (histone H3) further confirmed the interaction between ETV4, MCM2, FACT, and histone H3 proteins (Figure 3I–K). Similar to ETV4‐MCM2 binding, the N‐terminal deletion of ETV4 markedly decreased the interaction with SUPT16H and SSRP1 (Figure 3L). To further clarify the interactions between ETV4, MCM2, FACT, and histone H3 proteins, the His‐histone H3 and His‐ETV4 proteins were expressed and purified, and the lysates from A549 and H1299 cells were incubated with histone H3 or ETV4 combined Ni‐NTA His‐bind resin column to capture target proteins. According to the results of pull‐down assay, ETV4, MCM2, SUPT16H, and SSRP1 protein all bound to histone H3 (Figure 3M,N), and MCM2, SUPT16H, SSRP1, and histone H3 could bind to ETV4 protein (Figure 3O,P). In addition, we purified histone H3 (without tag), Flag‐tag MCM2 (1‐440aa), GST‐tag SUPT16H (1‐510aa), and Flag‐tag SSRP1 proteins and performed pull‐downs using His‐ETV4 as bait. It revealed that histone H3, SUPT16H, SSRP1, and MCM2 were pulled down individually by ETV4, indicating their direct interactions (Figure 3Q–T). The above data indicates that ETV4 interacts with MCM2 and histone chaperone FACT subunits, which raises the possibility that ETV4 plays an important role in the DNA replication of NSCLC cells.

### 2.4 MCM2, ETV4, and FACT are in Proximity to Newly Replicated DNA in NSCLC Cells

**Figure 4:**
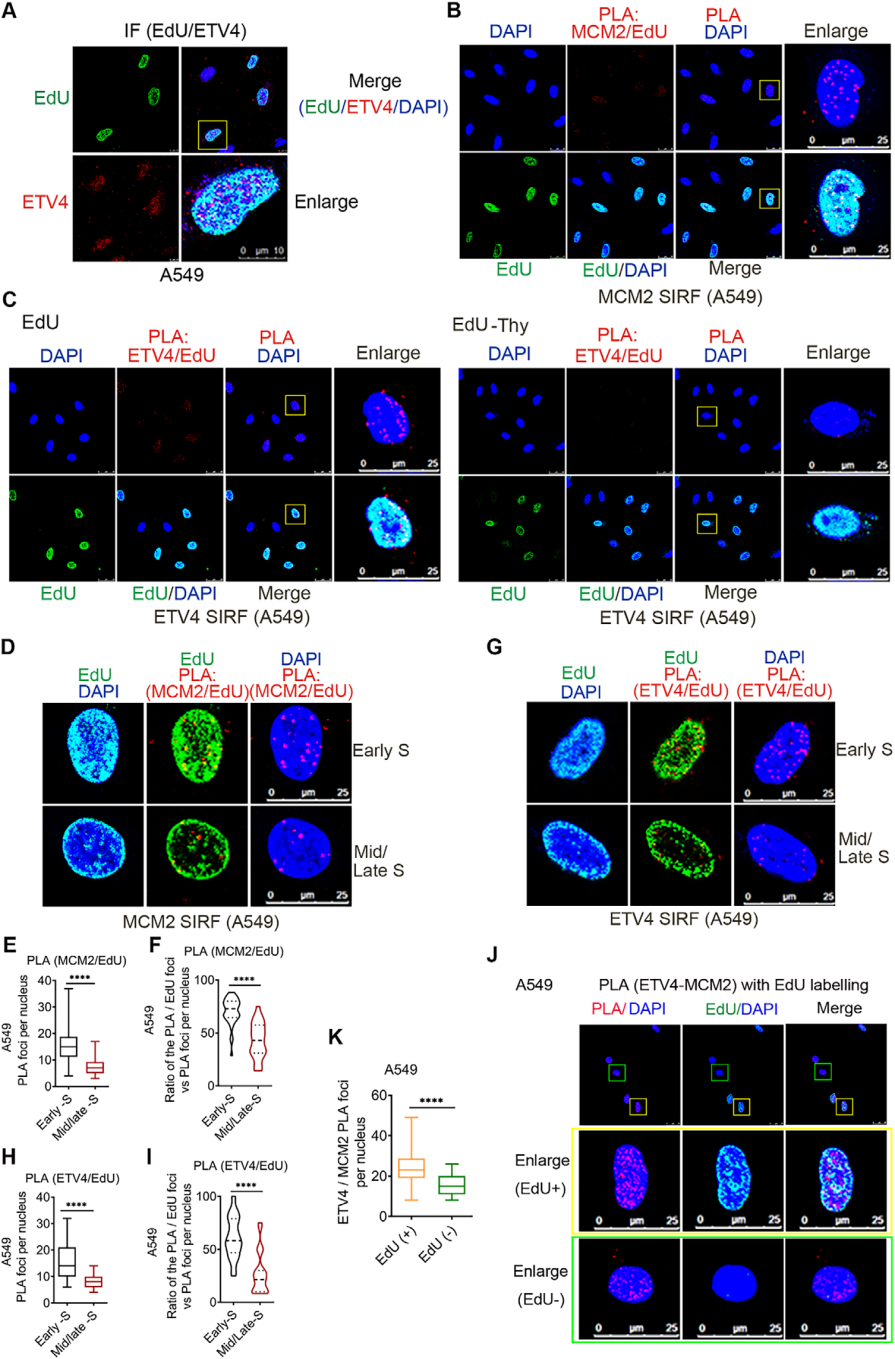
MCM2 and ETV4 are in proximity to newly replicated DNA in A549 cells. A) EdU‐labeling combined with the immunostaining method was used to detect the co‐localization of endogenous ETV4 with sites of replication (marked by EdU incorporation) in A549 cells after a 15 min EdU pulse and covalent linkage to a biotin‐azide using click chemistry. B) In situ proximity ligation assay with pulse EdU labeling (SIRF) assay showing the interaction of MCM2 with newly replicated DNA in A549 cells after labeled with 125 µm EdU for 15 min and click chemistry. PLA of MCM2/EdU (Red) was performed using anti‐biotin and anti‐MCM2 antibodies. FITC‐conjugated anti‐biotin IF (Green) indicates cells undergoing DNA synthesis. DNA was counterstained with DAPI (Blue). C) ETV4/EdU SIRF assay showing the interaction of ETV4 with newly replicated DNA in A549 cells labeled with 125 µm EdU for 15 min with or without 100 µM thymidine chases for 60 min. D) Representative images of cells with MCM2 SIRF signals in early or late S phase A549 cells. FITC‐EdU stains allow for the distinction between late S phase replication structures and early S‐phase cells. E,F) Average MCM2 SIRF (PLA foci) signals or the ratio of the PLA/EdU foci (yellow) to the PLA foci per nucleus in early and later S phase A549 cells. ^****^ *p* < 0.0001; two‐tailed Mann‐Whitney test. G) Representative images of cells with ETV4 SIRF signals in early S phase or late S phase A549 cells. H,I) Average ETV4 SIRF (PLA foci) signals or the ratio of the PLA/EdU foci (yellow) to the PLA foci per nucleus in early and later S phase A549 cells. ^****^ *p* < 0.0001; two‐tailed Mann‐Whitney test. J,K) PLA between ETV4‐MCM2 was performed using the Duolink® Proximity Ligation Assay, co‐stained of EdU with a Cell‐Light EdU Apollo 488 In Vitro Imaging Kit to label S phase cells, and analyzed the proportion of PLA signal in EdU‐positive vs. negative A549 cells. Average PLA foci per nucleus in each group were analyzed using the two‐tailed Mann‐Whitney test. ^****^ *p* < 0.0001.

To explore the possible role of ETV4 in DNA replication, EdU‐labeling combined with the immunostaining method was first used to determine whether ETV4 localizes at the replication foci (marked by EdU incorporation) in NSCLC cells. After a 15 min EdU pulse and biotinylated using click chemistry, it was found that the nuclear ETV4 signal was colocalized with replicating foci in A549 (**Figure** **4**A) and H1299 cells (Figure S8A, Supporting Information). Next, quantitative in situ analysis of protein interactions at DNA replication forks (SIRF)^[^ ^46^ ^]^ was carried out to see if ETV4 associates at the sites. We first measured MCM2, which is expected to be associated with active replication forks. SIRF signals against MCM2/EdU, as evidenced by red foci, were observed clearly in the nucleus of A549 cells (Figure 4B) and H1299 cells (Figure S8B, Supporting Information). Cells outside the S phase will remain EdU‐negative, and thus, will not display a PLA signal. In this setting, SIRF signal against ETV4/EdU (red foci) was also observed in the nucleus of A549 and H1299 cells, while it was decreased significantly after a 1 hr thymidine chase following the 15 min EdU pulse (Figure 4C; Figure S8C, Supporting Information). With a thymidine chase, the previously incorporated EdU is no longer present at an active replication fork, and replisome components will lose proximity with the biotinylated EdU.^[^ ^46^ ^]^ These results indicated that ETV4 is transiently near DNA replication forks during the S phase in A549 and H1299 cells. EdU is used to label S phase cells, and cells in the late S phase exhibit large replication foci, which can be distinguished from cells in the early S phase showing diffused signals throughout the nucleus.^[^ ^46^ ^]^ Further analysis showed that the higher MCM2‐SIRF (Figure 4D–F; Figure S8D,E, Supporting Information) and ETV4‐SIRF (Figure 4G–I; Figure S8F,G, Supporting Information) signals are associated with early S phase cells compared with late S phase cells both in PLA foci and the ratio of the PLA/EdU foci (yellow) to the PLA foci per nucleus of A549 and H1299 cells. In addition, FACT binds to several replisome components and travels with the replication fork.^[^ ^47^ ^]^ In line with this, SIRF assays confirmed that SUPT16H and SSRP1 both localized near newly replicating DNA (Figure S9A–D, Supporting Information). To further confirm the association of MCM2, FACT, and ETV4 on replicating DNA, we performed isolation of proteins on nascent DNA (iPOND) assay. Nascent DNA was labeled with EdU and conjugated with biotin, and proteins associated with biotin‐EdU‐labeled DNA were pulled down. We found that MCM2, SUPT16H, SSRP1, and ETV4 were enriched at/around DNA replication forks in A549 and H1299 cells (Figure S10 Supporting Information). To clarify whether ETV4‐MCM2 interaction occurs on nascent DNA, we performed PLA between ETV4 and MCM2 with co‐staining of EdU to label S phase cells. It showed that the intensity of ETV4‐MCM2 PLA signals in EdU positive cells was significantly higher than that of the EdU negative A549 (Figure 4J,K) and H1299 cells (Figure S11A,B, Supporting Information). Moreover, the ETV4‐SUPT16H PLA/EdU signals showed a similar change as ETV4‐MCM2 (Figure S11C–F, Supporting Information). These results demonstrate that ETV4, MCM2, and FACT are near newly replicated DNA, and their interactions occur on nascent DNA during DNA replication.

### 2.5 ETV4 Might Play Roles in Preserving Histone and its PTMs Near Sites of DNA Replication in NSCLC Cells

**Figure 5:**
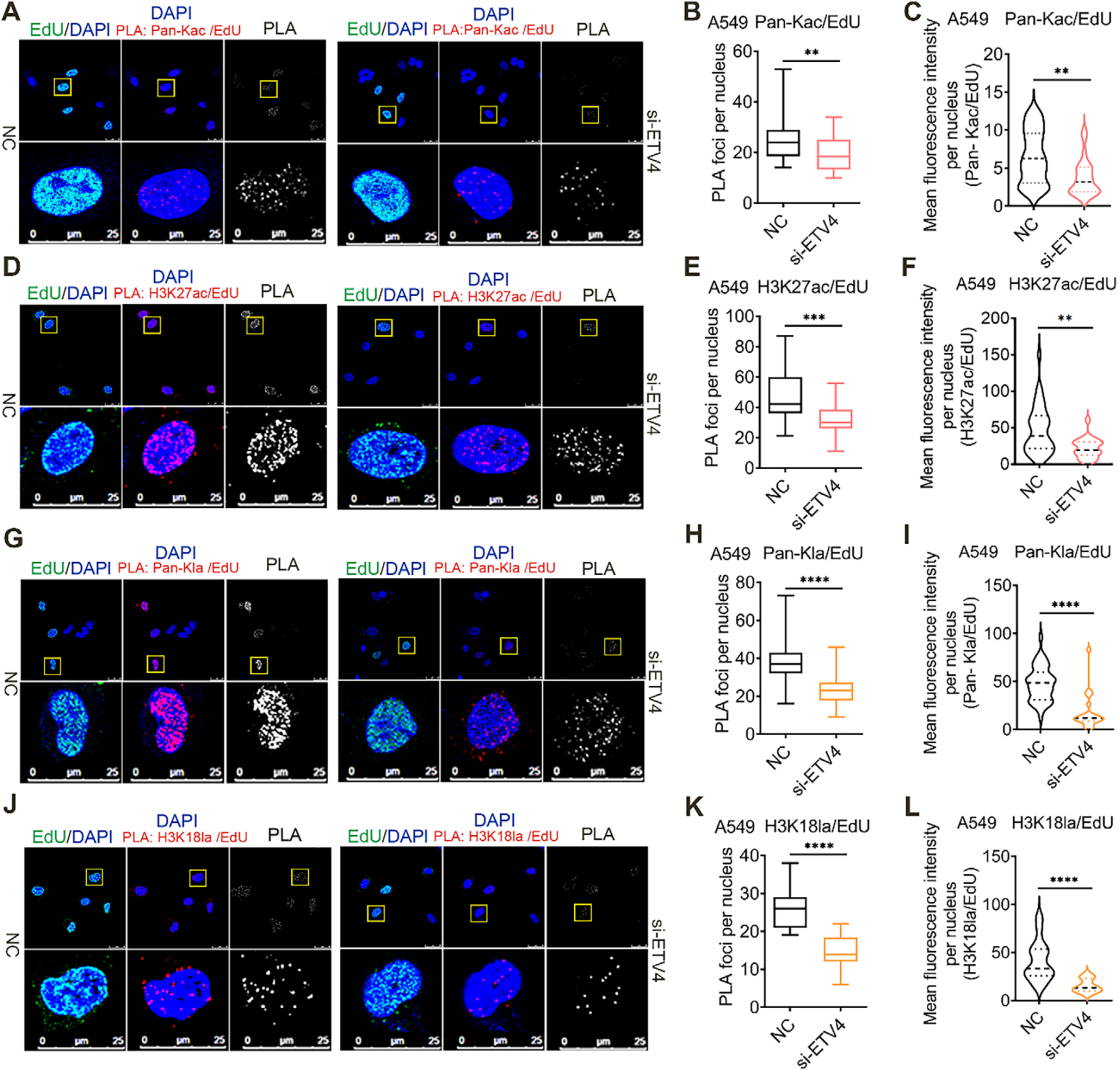
ETV4 plays roles in maintaining histone acetylation and lacylation near sites of DNA replication in A549 cells. A) Chromatin Assembly Assay (CAA) showing the pan‐Kac/EdU signals at the newly replicated DNA in A549 cells pulsed with 125 µM EdU for 15 min. FITC‐conjugated anti‐biotin IF (Green) indicates cells undergoing DNA synthesis. DNA was counterstained with DAPI (Blue). B,C) Quantification of the average pan‐Kac CAA foci per nucleus and the intensity of Immunofluorescence per nucleus in A549 cells. D) CAA for H3K27ac/EdU in A549 cells. E,F) Quantification of the average H3K27ac CAA foci per nucleus and the intensity of Immunofluorescence per nucleus in A549 cells. G–I) CAA for pan‐Kla/EdU in A549 cells as in (A–C). J–L) CAA for H3K18la/EdU in A549 cells as in A‐C. All CAA data were analyzed using the two‐tailed Mann‐Whitney test. For all statistical tests, ^**^ *p* < 0.01; ^***^ *p* < 0.001; ^****^ *p* < 0.0001.

Parental histones and the PTMs landscape are duplicated during DNA replication. PTM occupancy patterns are reproduced on newly replicated DNA with high accuracy in both repressed and active genomic regions, demonstrating that the positional information of histone marks is faithfully inherited during DNA replication.^[^ ^48^ ^]^ To elucidate the possible role of ETV4 in proximity to newly replicated DNA, we performed Chromatin Assembly Assay (CAA), the only method currently for quantitative detection of specific histone modifications in the nascent chromatin at or behind replication forks in single cells.^[^ ^49^, ^50^ ^]^ We first determined whether MCM2 N‐terminal (including HBD) deletion disturbs histone processing during replication using histone acetylation marker H3K27ac. Indeed, MCM2‐ΔN markedly decreased the H3K27ac/EdU CAA signals compared to the MCM2‐FL group in endogenous MCM2 knocked‐down H1299 cells, confirming the role of MCM2 N‐terminal in histone‐binding at replication forks (Figure S12A,B, Supporting Information). In addition, MCM2‐ΔN and/or ETV4‐ΔN deletion led to a significant decrease of H3K27ac/EdU signals compared with ETV4/MCM2‐FL group in endogenous ETV4 and MCM2 knocked‐down H1299 cells, suggesting that ETV4‐MCM2 N‐terminal region is essential for maintaining chromatin acetylation near sites of DNA replication (Figure S12C,D, Supporting Information). Based on the setting, we next performed CAA after ETV4 knockdown in NSCLC cells. CAA signals against pan‐Kac/EdU and H3K27ac/EdU were colocalized with DAPI. At the same time, the PLA foci and the mean fluorescence intensity per nucleus both were decreased significantly in si‐ETV4 A549 (**Figure** **5**A–F) and H1299 (Figure S13A–F, Supporting Information) cells. It was recently reported that Lysine L‐lactylation (Kla) is a newly discovered histone marker.^[^ ^51^ ^]^ Therefore, we performed CAA using pan‐Kla and H3K18la to analyze histone lactylation presented near replication forks. Similar to the results of histone acetylation, ETV4 silencing decreased the accumulation of CAA signals of both the pan‐Kla/EdU and H3K18la/EdU in A549 (Figure 5G–L) and H1299 (Figure S13G–L, Supporting Information) cells. Moreover, FACT subunit SUPT16H knockdown significantly decreased the H3K27ac/EdU and H3K18la/EdU CAA signals (Figure S14A–C, Supporting Information). Together, these data highlight the importance of localized histone acetylation and lactylation at replicating DNA and increase the likelihood that ETV4 has roles in preserving histone and its PTMs together with histone chaperone MCM2‐FACT during replication.

### 2.6 ETV4 Transcriptionally Controls Key Genes Involved in DNA Replication of NSCLC Cells

**Figure 6:**
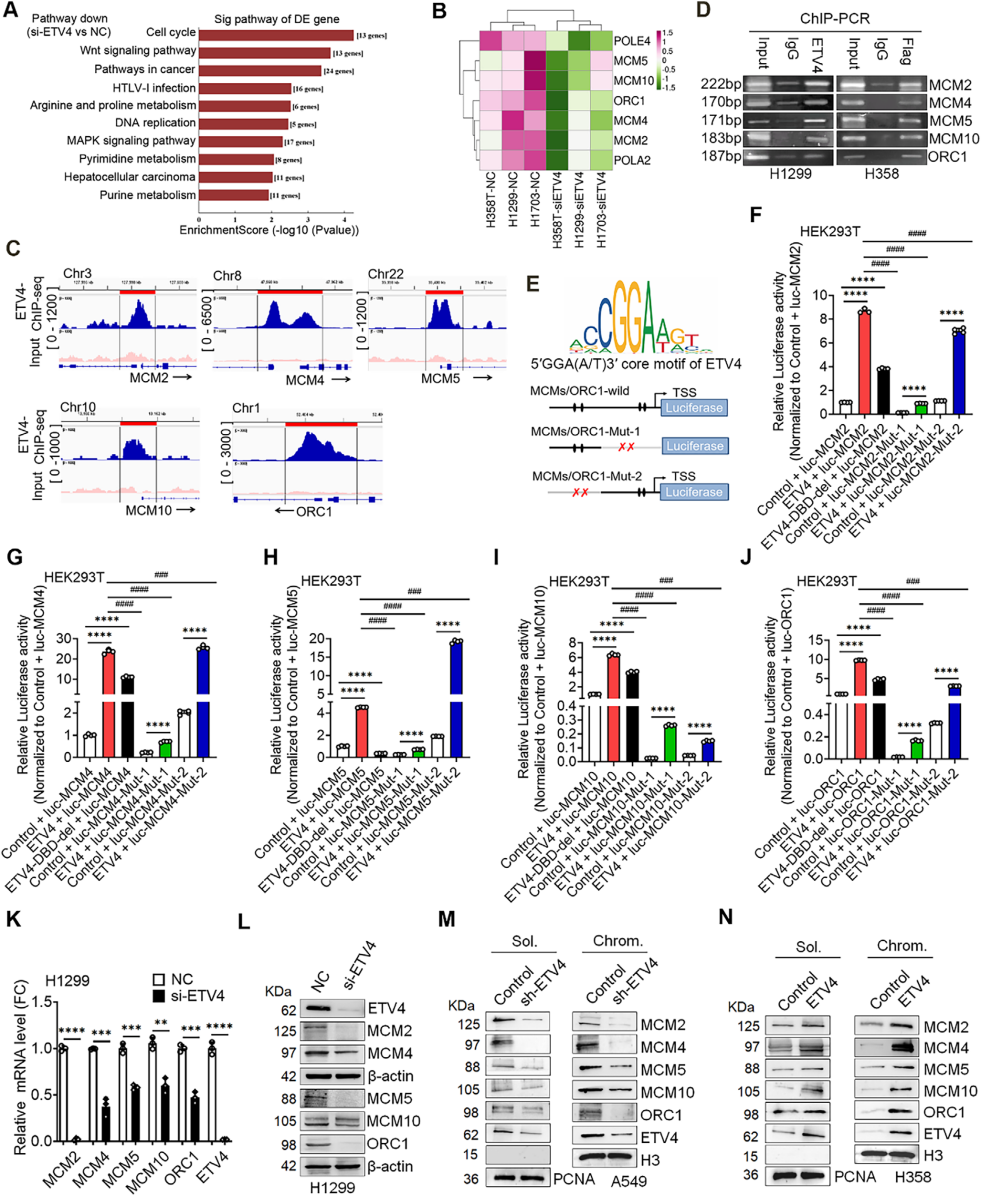
ETV4 transcriptionally controls key genes involved in DNA replication of NSCLC cells. A) Functional analysis showing the top KEGG pathways that are significantly associated with down‐regulated genes in H1299, H1703, and H358T si‐ETV4 groups normalized to negative control (NC) siRNA groups by Human Microarray (GSE137445). B) Clustering analysis showing the gene signature is enriched in the DNA replication pathway. C) IGV tracks showing the enrichment of ETV4 at the promoter region of MCM2, ‐4, ‐5, ‐10, and ORC1 from ChIP‐seq signals of A549‐shETV4 cells transfected with Flag‐ETV4 plasmids. D) ChIP‐PCR analysis for endogenous or exogenous ETV4 binding to the promoter region of MCM2, ‐4, ‐5, ‐10, and ORC1 genes in H1299 cells using anti‐ETV4 antibody or H358‐ETV4 cells using anti‐Flag antibody. E) Schematic depicting the core motif of ETV4 binding with its target genes, the wild‐type luciferase reporter constructs, and the mutations in the putative ETV4‐binding sites (mut‐1 or mut‐2 type) of MCMs/ORC1 promoter region. F–J) The wild‐type luciferase reporter plasmids of MCMs (ORC1) were co‐transfected along with ETV4, ETV4‐DBD deletion plasmid, or the empty vector into HEK293T cells. The constructed luc‐MCMs (ORC1)‐mut‐1 or ‐mut‐2 type plasmids were co‐transfected along with ETV4 plasmid or the empty vector into HEK293T cells. Relative luciferase activity was normalized against Renilla luciferase activity, respectively (mean ± SD; *n* = 4; ordinary one‐way ANOVA with Tukey's multiple comparisons test). ^****^ *p* < 0.0001; ^###^ *p* < 0.001, ^####^ *p* < 0.0001. K) RT‐qPCR analysis of MCM2, ‐4, ‐5, ‐10, and ORC1 mRNA expression in H1299 cells transfected with ETV4 or NC siRNA, Transcript levels were normalized to ACTB gene expression (mean ± SD, *n* = 3; two‐tailed unpaired *t*‐test). ^**^ *p* < 0.01; ^***^ *p* < 0.001; ^****^ *p* < 0.0001. L) Immunoblots showing MCM2, ‐4, ‐5, ‐10, and ORC1 protein levels in NC and ETV4‐knockdown H1299 cells. M,N) Immunoblots showing the Chromatin‐bound proteins (Chrom.) and unbound proteins (Sol.) levels of MCM2, ‐4, ‐5, ‐10, and ORC1 protein in control and sh‐ETV4 A549 cells, or control and ETV4‐overexpression H358 cells.

To determine whether ETV4 can regulate the expression of genes involved in DNA replication and cell cycle transition to transcriptional control DNA replication, we checked our previous microarray data (GSE137445) and found that the cell cycle and DNA replication signaling were among the top down‐regulated pathways in si‐ETV4‐transfected H1299, H1703, and H358T NSCLC cells (**Figure** **6**A). H358T is TGF‐β‐induced EMT‐ed H358 cells with higher ETV4 expression than H358 cells (Figure S2A, Supporting Information). Among those genes, the replicative helicase‐MCM2, MCM4, MCM5, origin licensing factor‐ORC1, and firing factor‐MCM10 were significantly inhibited after ETV4 knockdown (Figure 6B). Consistent with the microarray results, our ChIP‐seq data showed that ETV4 was enriched in the promoter region of MCM2, ‐4, ‐5, ‐10, and ORC1 genes (Figure 6C). Thus, we next focused on the regulatory role of ETV4 on the five replisome genes. ChIP‐PCR experiments with anti‐ETV4 (H1299, endogenous) or anti‐Flag (H358, exogenous) demonstrated the binding of ETV4 at the promoter of MCMs and ORC1 genes (Figure 6D). To further determine the effects of ETV4 on their promoter activity, we performed luciferase assays after deletion of the DNA binding domain of ETV4 (ETV4‐DBD, 337–430 aa) or mutation in the putative ETV4‐binding sites on the promoter of MCMs/ORC1 genes. As shown in Figure 6E–J, ETV4 overexpression significantly increased the luciferase activity of wild‐type MCMs and ORC1 promoter‐reporter. In contrast, deletion of the DBD domain of ETV4 or the putative ETV4‐binding sites near the transcription starting site (TSS) decreased the promoter activity. RT‐qPCR and immunoblot results verified the effects of ETV4 on MCM2, ‐4, ‐5, ‐10, and ORC1 mRNA and protein expressions (Figure 6K,L; Figure S15A–F, Supporting Information). In addition, the MCMs and ORC1 expression levels were significantly lower in A549‐shETV4 cells. In the contrast, they were higher in H358 cells stably overexpressing ETV4 (H358‐ETV4) than in their control cells, both in chromatin‐bound proteins (Chrom.) and unbound proteins (Sol.) (Figure 6M,N). Furthermore, the effects of ETV4 on MCM2, ‐4, ‐5, ‐10, and ORC1 protein expression and chromatin recruitment were confirmed in A549‐shETV4 cells after re‐transfected with Flag‐ETV4 (Figure S15G,H, Supporting Information). These data indicate that ETV4 transcriptionally controls the key replisome genes MCM2, ‐4, ‐5, ‐10, and ORC1 expression and affects their recruitment to chromatin in NSCLC cells.

### 2.7 Elevated ETV4‐MCMs/ORC1 Axes are Associated with Cell Proliferation, Tumor Growth, and Poor Prognosis of NSCLCs

**Figure 7:**
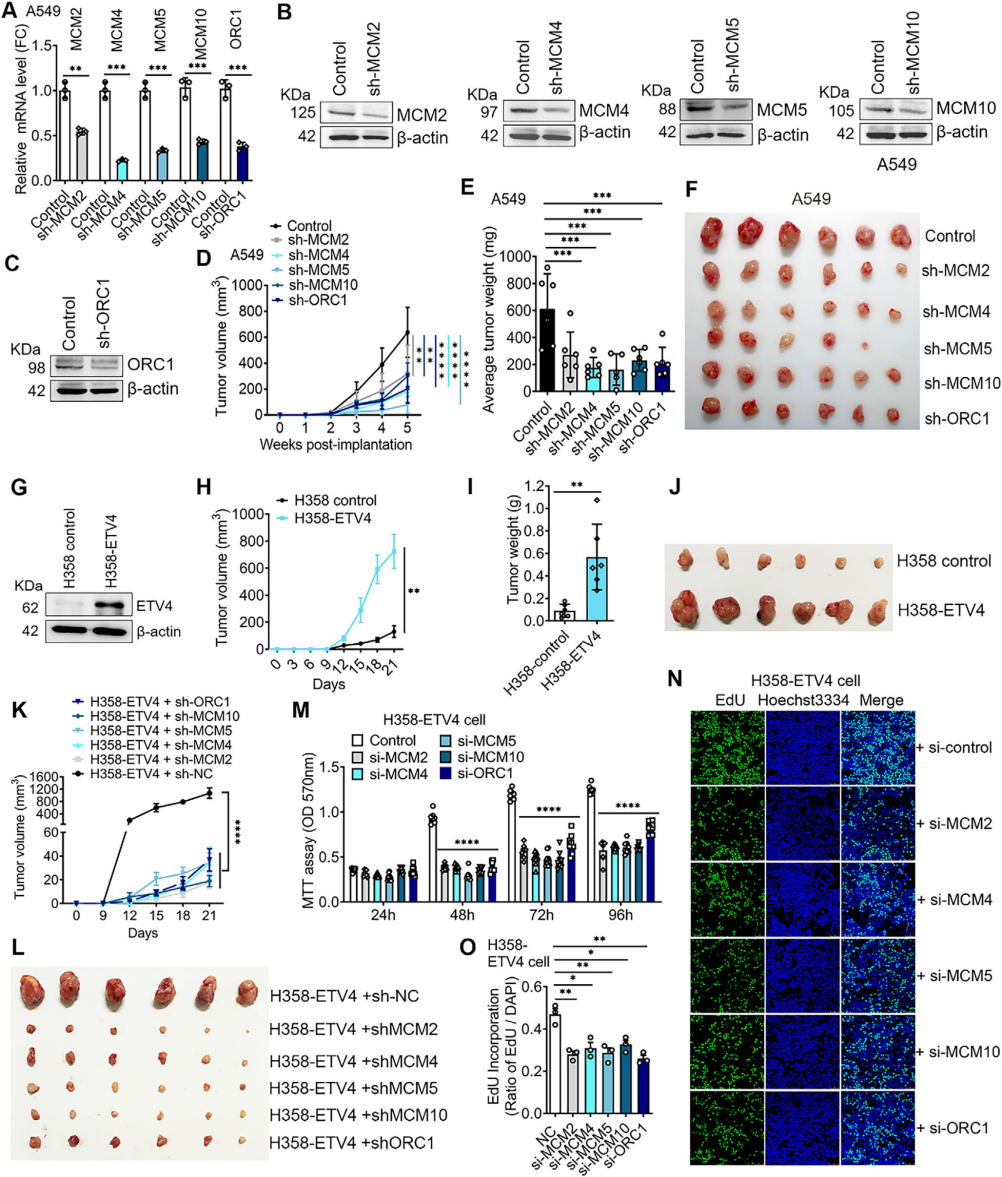
MCMs and ORC1 are important regulators of ETV4‐dependent tumorigenic properties in NSCLC cells. A–C) A549 cells stably expressing shRNA targeting MCM2, ‐4, ‐5, ‐10, ORC1, or negative control were generated using LV3/Puro vector. Gene expressions were detected by RT‐qPCR and Western blot, respectively. (For RT‐qPCR assays, mean ± SD, *n* = 3). ^**^ *p* < 0.01; ^***^ *p* < 0.001. D) Growth curves of xenograft tumors after the subcutaneous injection of A549 cells stably knockdown of MCM2, ‐4, ‐5, ‐10, ORC1, or negative control. The tumor volumes were measured every week after injection, *n* = 6. Ordinary one‐way ANOVA with Tukey's multiple comparisons test). ^**^ *p* < 0.01; ^****^ *p* < 0.0001. E) The average weight of tumors derived from each group was measured by week 5 after injection. Ordinary one‐way ANOVA. ^***^ *p* < 0.001. F) Representative images of tumors from each group excised from mice 5 weeks after injection. G) H358 cells stably expressing ETV4 or control (empty vector) were generated. ETV4 protein expression was detected by Western blot assay. H) Growth curves of xenograft tumors in athymic BALB/c nude mice after the subcutaneous injection of H358‐ETV4 or control cells. The tumor volumes were measured every 3 days after injection. *n* = 6. Two‐tailed unpaired *t‐*test. ^**^ *p* < 0.01. I) The average weight of tumors derived from H358‐ETV4 cells or control groups was measured 21 days after injection. *n* = 6. Two‐tailed unpaired *t‐*test. ^**^ *p* < 0.01. J) Representative images of tumors excised from mice 21 days after injection. K) H358‐ETV4 cells stably expressing shRNA targeting MCM2, ‐4, ‐5, ‐10, ORC1, or negative control were generated after double selection using neomycin and puromycin to perform the in vivo tumor growth assay. The tumor volumes were measured every 3 days after the subcutaneous injection. The graph represents tumor volume (mm^3^) versus time 3 weeks after injection (*n* = 6). Ordinary one‐way ANOVA with Tukey's multiple comparisons test). ^****^ *p* < 0.0001. L) Representative images of tumors excised from mice 21 days after injection from H358‐ETV4 + sh‐control cells and H358‐ETV4 + sh‐MCMs/ORC1 cells. M) Cell viability was evaluated using the MTT assay in H358‐ETV4 cells transfected with MCM2, ‐4, ‐5, ‐10, ORC1, or NC siRNA. (mean ± SD; *n* = 8; Two‐way ANOVA with Bonferroni's multiple comparisons test). ^****^ *p* < 0.0001. N,O) DNA replication was evaluated using EdU‐incorporation assays in H358‐ETV4 cells transfected with MCM2, ‐4, ‐5, ‐10, ORC1, or NC siRNA. Replicating cells label with EdU. (mean ± SD; *n* = 3; Two‐tailed unpaired *t‐*test). ^*^ *p* < 0.05; ^**^ *p* < 0.01.

To determine the effects of MCMs and ORC1 involved in cell proliferation, we knocked down their expression in H1299, H1703, and H358T cells using specific siRNA transfection (Figure S16A,B, Supporting Information). MCMs and ORC1 knockdown reduced proliferation and EdU incorporation in the three cells based on MTT and EdU assay (Figure S16C–E, Supporting Information). In the subcutaneously implanted model, tumor growth was significantly inhibited in sh‐MCM2, ‐4, ‐5, ‐10, and sh‐ORC1 groups compared with the control A549 group by week 5 (**Figure** **7**A–F). To determine the role of ETV4 on tumor growth, H358 control or H358‐ETV4 cells were used in the subcutaneously implanted model. Mice injected with H358‐ETV4 cells initiated tumor growth at ≈12 d, and the tumors grew faster than those of control groups. The weight and size of the tumors derived from H358‐ETV4 cells were significantly larger than those of the control groups by week 3 (Figure 7G–J). To determine if MCMs/ORC1 participate in ETV4‐regulated tumor growth in vivo, we stably repressed MCMs/ORC1 in the H358‐ETV4 cells by sh‐MCMs or sh‐ORC1 transfection and double antibiotic‐resistant cell selection (Figure S17A–C, Supporting Information). Strikingly, depletion of MCMs or ORC1 almost completely inhibited tumorigenesis of H358‐ETV4 cells in vivo for 21 days when the average tumor volume derived from the control group reached ≈1000 mm^3^ (Figure 7K,L). Besides, the depletion of MCMs/ORC1 using siRNA transfection significantly inhibited H358‐ETV4 cell proliferation and EdU incorporation (Figure 7M–O). Moreover, overexpression of ETV4 significantly reversed the inhibitory effects of MCM2, ‐4, ‐5, ‐10, or ORC1 deletion on cell proliferation and EdU incorporation of H358 cells (Figure S18A–D, Supporting Information). These results confirm that MCM2, ‐4, ‐5, ‐10, and ORC1 are critical mediators of ETV4 oncogenic activity in NSCLC cells.

To investigate the clinical significance of the ETV4‐MCMs/ORC1 axis involved in NSCLC progression, we analyzed their expression and relationships in human NSCLC tissues. At the mRNA level, TCGA data indicated that ETV4, MCM2/4/5/10, and ORC1 mRNA levels were all significantly upregulated in 515 lung adenocarcinoma (LUAD) and 503 lung squamous cell carcinoma (LUSC) compared with normal lung tissue. The significant positive co‐expression patterns between ETV4 and MCM2/4/5/10, ORC1 mRNA in LUSC and LUAD were confirmed by Spearman's correlation analysis (Figure S19A–D, Supporting Information). In addition, IHC analysis was performed in FFPE sections of 81 NSCLC patients. Positive nuclear ETV4 staining was observed in 49 of 81 (60.49%) tumor tissues, which was positively associated with tumor size, lymph node metastasis, and TNM stage (all *p* < 0.01, Figure S19E, Table S1, Supporting Information). The nuclear staining of MCM2, ‐4, ‐5, ‐10, and ORC1 was positive in 56.79%, 55.56%, 51.85%, 44.44%, and 50.62% of tumor tissues, respectively, and were all positively associated with tumor size, lymph node metastasis, and TNM stage (all *p* < 0.05, Figure S19E, Table S1,S2, Supporting Information). As indicated in Figure S19F, ETV4 expression was positively correlated with MCM2 (r = 0.468, *p* < 0.001), MCM4 (r = 0.497, *p* < 0.001), MCM5 (r = 0.434, *p* < 0.001), MCM10 (r = 0.520, *p* < 0.001), as well as ORC1 expression (r = 0.515, *p* < 0.001) in the human NSCLC specimens. Besides, Kaplan‐Meier analysis showed that the overall survival rates of the patients were inversely correlated with ETV4 (Log‐rank *p* < 0.001), MCM2 (Log‐rank *p* = 0.006), MCM4 (Log‐rank *p* = 0.014), MCM5 (Log‐rank *p* = 0.002), MCM10 (Log‐rank *p* = 0.002), and ORC1 protein expression (Log‐rank *p* = 0.002), respectively (Figure S19G, Supporting Information). In addition, the patients with ETV4^+^/MCM2^+^, ETV4^+^/MCM4^+^, ETV4^+^/MCM5^+^, ETV4^+^/MCM10^+^, or ETV4^+^/ORC1^+^ co‐expression showed worse prognosis than that of the others (Figure S19H, Supporting Information). Thus, elevated ETV4‐MCMs/ORC1 axes could be biomarkers to predict the metastasis and poor prognosis of NSCLCs.

### 2.8 High‐ETV4 Expression Leads to DNA Damage and R‐Loop Formation in Response to Replication Stress Induced by TOP1 Inhibitor‐Camptothecin (CPT)

**Figure 8:**
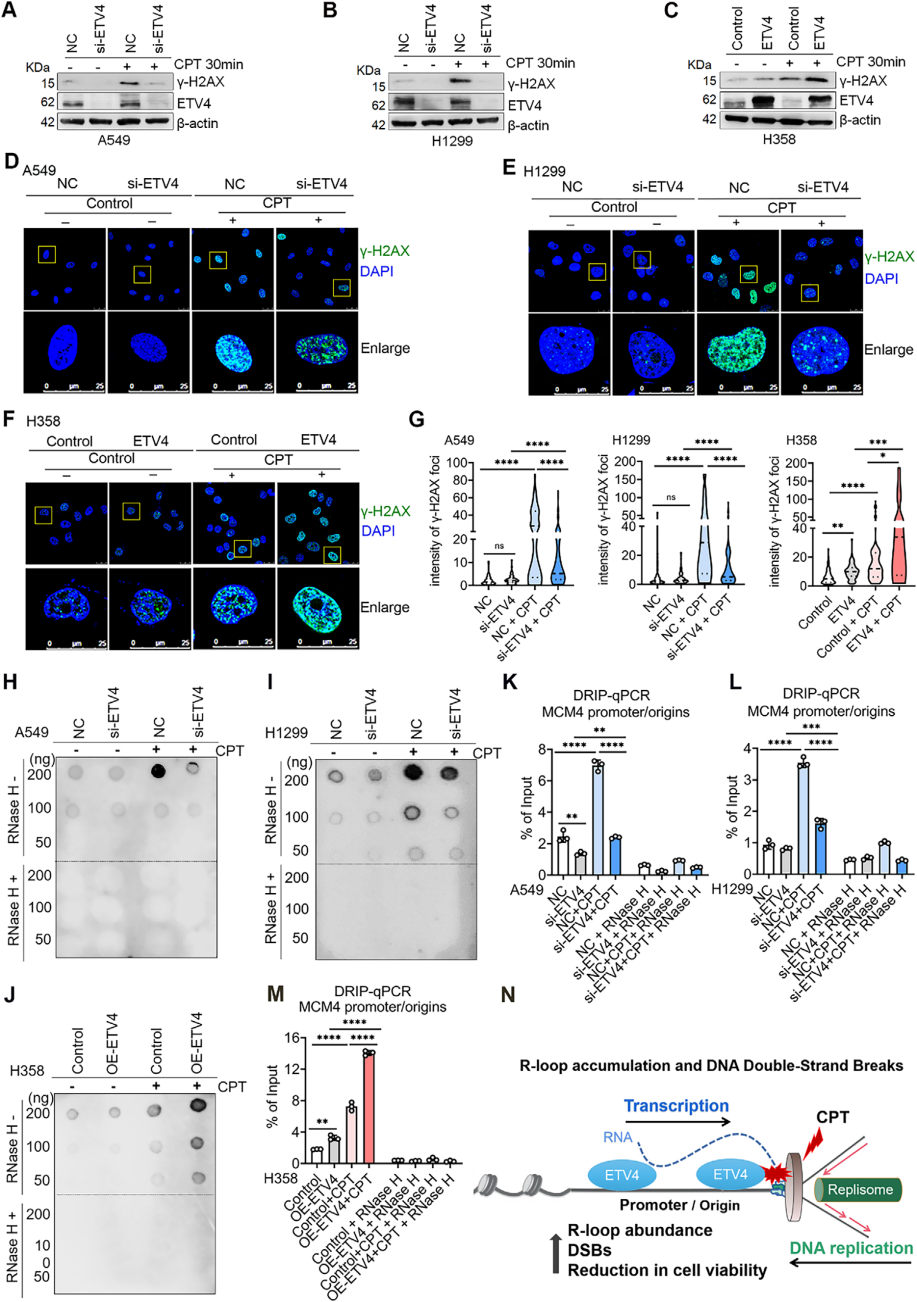
High‐ETV4 expression leads to DNA damage and R‐loop formation in response to replication stress induced by TOP1 inhibitor CPT. A–C) Immunoblots showing γ‐H2AX protein levels in NC and ETV4‐knockdown A549 and H1299 cells, or control and ETV4‐overexpression H358 cells in the presence or absence of 25 µM CPT treatment for 30 min. D–F) Immunofluorescence assay showing the accumulation of γ‐H2AX foci in NC and ETV4‐knockdown A549 and H1299 cells, or control and ETV4‐overexpression H358 cells with or without CPT treatment. G) Violin plot quantification of γ‐H2AX foci intensity per cell as shown in D‐F. *P*‐values for γ‐H2AX signals were based on the Kruskal‐Wallis test with Dunnett's multiple comparisons. ^*^ *p* < 0.05; ^**^ *p* < 0.01; ^***^ *p* < 0.001; ^****^ *p* < 0.0001; n.s., not significant. H–J) Dot blot analysis showing the abundance of R‐loop in ETV4‐knockdown A549 and H1299 cells, or control and ETV4‐overexpression H358 cells treated with or without CPT using the S9.6 antibody. RNase H treatment was included as a negative control. K–M) DRIP‐qPCR assays mapping the R‐loop accumulation at MCM4 promoter/origins loci in ETV4‐knockdown A549 and H1299 cells or H358‐ETV4 cells treated with or without CPT using the S9.6 antibody. RNase H treatment was included as a negative control. Ordinary one‐way ANOVA with Tukey's multiple comparisons test. ^*^ *p* < 0.05; ^**^ *p* < 0.01; ^***^ *p* < 0.001; ^****^ *p* < 0.0001. N) The proposed working model of high‐ETV4 expression leads to DNA damage and R‐loop formation in response to replication stress induced by CPT.

Both early DNA replication and gene transcription occur in active chromatin compartments, and transcription‐replication collisions (TRCs) within gene bodies lead to gross DNA damage.^[^ ^52^ ^]^ Because ETV4 binds to the origin‐promoter locus like the MCM4 gene, we wonder if ETV4 is related to collisions and DNA double‐strand breaks (DSBs). To this end, we examined the DNA damage marker γ‐H2AX level after ETV4 knockdown or overexpression with or without TOP1 inhibitor CPT treatment. TOP1 cleaves one strand of supercoiled DNA structures ahead of DNA replication or transcription to release torsional stress on the DNA, facilitating uninterrupted DNA synthesis and transcription.^[^ ^53^ ^]^ CPT induces replication‐associated DSBs when the replication fork collides with the ‘‘trapped’’ topo I–DNA complexes (Top1cc).^[^ ^54^, ^55^ ^]^ Recent study revealed an early replication‐specific and transcription‐dependent mechanism of genome instability elicited by CPT‐induced Top1ccs, which increases TRCs, DNA‐RNA hybrids (R‐loop), and DSBs at early/mid‐replicating initiation zones.^[^ ^56^ ^]^ In untreated conditions, the γ‐H2AX level detected by immunoblots showed slight differences following ETV4 knockdown or overexpression. However, it was effectively inhibited in si‐ETV4 knockdown A549 and H1299 cells but was induced in H358‐ETV4 cells upon CPT treatment (**Figure** **8**A–C). In addition, the accumulation of γ‐H2AX foci by Immunofluorescence (IF) assays further confirmed the correlation between ETV4 expression and CPT‐induced DSBs (Figure 8D–G). The mediator of DNA damage checkpoint protein 1 (MDC1) is necessary for the high‐density accumulation of γ‐H2AX directly surrounding the DNA lesion.^[^ ^57^ ^]^ Consistent with the results of γ‐H2AX foci, MDC1 foci formation was substantially suppressed in ETV4 knockdown cells but was induced in ETV4 overexpressing cells after CPT treatment (Figure S20A–D, Supporting Information).

R‐loop is a triple‐stranded structure composed of an RNA: DNA hybrid and a single DNA strand. It occurs naturally during transcription and has important physiological functions, such as gene expression and DNA replication. However, abnormal accumulation of R‐loops poses a threat to genome stability when encountered by the replication machinery, leading to replication stress and DSB formation.^[^ ^52^, ^58^ ^]^ We wondered if R‐loop formation played a role in controlling DSB formation. Therefore, we quantified global R‐loop levels using dot blot analysis with a monoclonal antibody (S9.6) that specifically recognizes RNA:DNA hybrids in a sequence‐independent manner. Indeed, the absence of ETV4 resulted in a marked decrease in the overall abundance of R‐loop in A549 and H1299 cells treated with CPT (25 µM, 30 min), which were sensitive to RNase H treatment. In contrast, upon CPT treatment, a significant increase in the abundance of RNase H‐sensitive R‐loop was observed in H358‐ETV4 cells (Figure 8H–J). As R‐loop form cotranscriptionally, we further evaluated whether RNAPII‐mediated transcription inhibitor‐DRB affected R‐loop and γ‐H2AX formation. Indeed, DRB treatment strongly reversed CPT‐induced γ‐H2AX formation and R‐loop level in A549 and H1299 cells as well as in H358‐ETV4 cells (Figure S21A–L, Supporting Information), indicating that TOP1 poisoning by CPT also causes transcription‐dependent R‐loop mediated DSBs. Consistently, ETV4‐ΔDBD led to a decrease of γ‐H2AX and R‐loop formation compared to the ETV4‐FL group in CPT‐treated H358 cells. In addition, the ETV4‐ΔDBD construct decreased the EdU incorporation of H358 cells compared with the ETV4‐FL transfection (Figure S22A‐F, Supporting Information). These results indicate that ETV4 induces DSBs in a manner dependent on R‐loop and replication under CPT treatment. To further clarify the relationship between ETV4 and CPT‐induced R‐loop accumulation at precise genomic loci, we performed DNA‐RNA immunoprecipitation (DRIP)‐qPCR assays to quantify R‐loop levels at two selected loci, EGR1 (previously known to have a propensity to form R‐loops)^[^ ^59^ ^]^ and the origin‐promoter locus of MCM4. Integrative analysis of our ETV4 ChIP‐seq data with the identified R‐loop forming sequence (RLFS) from the R‐loop database (R‐loop DB) showed that ETV4 binding overlapped with the RLFS at EGR1 and MCM4 loci (Figure S23A,B, Supporting Information). DRIP‐qPCR results in H1299 cells indicated that CPT treatment induced R‐loop accumulation at EGR1 and MCM4 loci when compared to the control group, and these were sensitive to RNase H treatment (Figure S23C,D, Supporting Information). More importantly, ETV4 knockdown in A549 and H1299 cells resulted in a significant decrease in CPT‐induced R‐loop in an RNase H‐dependent manner, whereas ETV4 overexpression in H358 cells increased the abundance of RNase H‐sensitive R‐loop at MCM4 (Figure 8K–M) and EGR1 loci (Figure S23E–G, Supporting Information). These findings suggest that ETV4 expression is related to CPT‐induced R‐loop accumulation at the overall level and specific genomic loci. The above data indicate that high‐ETV4 expression enhances R‐loop accumulation and DSB formation in response to CPT‐induced replication stress.

In addition, MTT and clonogenic assays indicated that CPT treatment significantly reduced cell viability and clonogenicity in H358‐ETV4 cells compared to the control cells (Figure S24A–C, Supporting Information). It was reported that cisplatin's cytotoxic activity is partly from the poisoning of TOP1, which is exacerbated in the presence of CPT analogs topotecan in MCF‐7 and WiDr cells.^[^ ^60^ ^]^ We further detected the effects of CPT combined with cisplatin on cell survival after ETV4 knockdown or overexpression. Clonogenic assay results indicated that CPT or cisplatin treatment strongly impaired the cell survival of H1299 and A549 cells in the NC group, whereas si‐ETV4 did not further increase sensitivity to the drugs. In contrast, ETV4 overexpression hypersensitized H358 cells to CPT and CPT/cisplatin combination treatment. Consistently, MTT results showed that cisplatin reduced the IC_50_ of CPT in H358‐ETV4 cells (Figure S24D–F, Supporting Information), suggesting the synergistic effects on cell survival. The above results indicate that NSCLC cells with higher ETV4 expression are sensitive to TOP1 poisoning‐induced DNA damage (Figure 8N).

## 3 Discussion

Deregulation of DNA replication is a widespread phenomenon in carcinogenesis. We describe here the critical functions of the cancer‐driven gene ETV4 in DNA replication in NSCLC (Figure S25, Supporting Information). It is located at specific human DNA replication origins and assists the loading of ORC at the origin by interacting with ORC subunits ORC1 and ORC6. ETV4 interacts with histone chaperone factors MCM2‐FACT and might be involved in histone processing during DNA replication. In addition, ETV4 transcriptionally controls the key replisome genes MCM2, ‐4, ‐5, ‐10, and ORC1 expression and affects tumor growth and prognosis of NSCLC patients. Since ETV4 is located at the origin‐promoter locus like the MCM4 gene, simultaneous engagement of replication origins and transcription might be a critical mechanism of ETV4 to coordinate both processes flexibly. However, upon external replication stress caused by TOP1 inhibitor, ETV4 overexpression is prone to induce the accumulation of unscheduled R‐loops, DNA damage, and cell death. These findings identify that ETV4 controls DNA replication via transcriptional and non‐transcriptional mechanisms in NSCLC cells.

The heterohexameric ORC1‐6 acts as a scaffold for the G1 phase assembly of pre‐RC at replication origins.^[^ ^61^ ^]^ Human ORC1‐5 core complex and ORC6 can bind independently to DNA, and their dynamic association is related to ORC3, CDC6 and ORC chaperone protein‐HMGA1a, which are recruited efficiently by ORC6 to stabilize ORC. This dynamic association model allows flexible associations of ORC6 with different partners to conduct its diverse cellular functions. Therefore, the ORC6 protein qualifies as a regulatory subunit of ORC that fulfills integrating functions at different stages of the replication initiation process and the cell cycle.^[^ ^40^, ^41^, ^42^ ^]^ Our molecular docking analysis and Co‐IP results suggested that ETV4 might bind with dsDNA‐ORC mainly through ORC1 and ORC6 subunits. ChIP results demonstrated that ETV4 could affect the recruitment of ORC6 at LAMB2 and MCM4 origins. Loss of ORC6 caused a reduction in ORC1 enrichment at origins and partially reversed the effects of ETV4 overexpression induced origin‐associated ORC1 levels. Moreover, we found that the ETV4 N‐terminal is much more essential for the binding with ORC6 and ORC1, and deletion of the N‐terminal led to a significant decrease of ORC6/ORC1 recruitment to both origins. Our results indicated that ETV4 may function as an ORC‐interacting protein and work together to promote replication origin formation. Additionally, DBD deletion decreased the binding of ETV4 at the DNA replication origins especially those located in the promoter region. In contrast, N‐terminal deletion decreased ETV4 binding at all the tested origins, indicating an unexpected role in replication origin‐DNA binding. Recent findings reported that the regions far from the DBD and commonly in intrinsically disordered regions (IDRs) can modulate DNA binding of TFs, particularly in the context of a chromatinized genome.^[^ ^62^ ^]^ Interestingly, we found that ETV4 protein bears IDRs in its N‐terminal at 90–115aa from the UniProt database. Although the N‐terminal amino acids in ETV4 origin‐DNA binding activity remain unclear and warrant further investigation, our data indicate that ETV4 regulates DNA replication depending on both of the DBD and the N‐terminal region through different mechanisms. It was recently reported that increased ORC6 expression is closely associated with tumor progression and an unfavorable prognosis in NSCLC patients.^[^ ^63^ ^]^ We found that depleting ORC6 significantly inhibited NSCLC cell proliferation, EdU incorporation, and S phase progression and reversed the effects of ETV4 on cell proliferation and EdU incorporation, which indicated the essential role of ORC6 in ETV4‐regulated tumor cell proliferation and replication. In addition, the largest subunit ORC1 is reported to be degraded after the initiation of replication; its rebinding to chromatin is an obligatory step for the establishment of the pre‐RC in G1.^[^ ^64^ ^]^ We found that ETV4 controls the total expression and chromatin‐bound ORC1, ensuring a sufficient amount for origin licensing in NSCLC cells. Together, our results support two possible mechanisms for ETV4 regulating origins licensing: one is locating at origins and regulating ORC recruitment by interacting with ORC subunits like ORC6 and ORC1, and the other one is ensuring a sufficient amount of ORC1 for its chromatin‐binding and origin licensing.

MCM2‐7 is the only complex present in both the pre‐RC and the active replisome and plays a crucial role in determining cellular replication potential. MCMs pool is sustained by recycling once‐licensed parental MCMs and synthesizing nascent MCMs in mother cells, ensuring that daughter cells receive a sufficient amount of licensing‐competent MCM units as soon as they enter the cell cycle.^[^ ^65^ ^]^ Excess DNA‐bound MCMs can be defined as “fork‐speed management” to enforce the pace of replication forks.^[^ ^66^ ^]^ We demonstrated that ETV4 transcriptionally controls MCM unit expression to sustain a sufficient amount and chromatin‐bound of MCM units, especially the MCM2/5 gate and MCM4. MCM2‐7 helicase subunit MCM2 has histone‐chaperone activity via the MCM2‐HBD and collaborates with histone chaperones (FACT or ASF1) to mediate the redeposition of new and old histones at replication forks.^[^ ^17^, ^18^, ^19^, ^20^ ^]^ Previous studies have reported that parental histones carrying repressive marks are recycled to their original genomic locations by the replisome, and those with active marks are also re‐incorporated close to their original positions, which may enable transcription to be resumed quickly and accurately after DNA replication.^[^ ^48^, ^67^, ^68^ ^]^ Interestingly, our data showed that ETV4 protein interacts with MCM2‐N terminal‐histone H3‐FACT subunits and is near newly replicated DNA using the SIRF and iPOND assays. This raises the question of whether ETV4 is located at the replication sites to help histones and their PTMs re‐associate after fork passage due to its sequence‐specific TF binding feature. To this end, we performed CAA to detect the level of histone PTMs in the nascent chromatin at or behind replication forks in single‐cell resolution.^[^ ^49^, ^50^ ^]^ Our preliminary data indicated that MCM2‐ΔN, ETV4‐ΔN, and ETV4 deletion decreased the histone PTM levels near the replication sites. Overall, we demonstrate that ETV4 exerts transcriptional and non‐transcriptional roles in assisting the function of the replicative helicase MCM2‐7 complex. However, the regulatory mechanisms of MCM2‐ETV4‐FACT in DNA replication and histone deposition are very complicated, additional work will be needed to fully elucidate its role in replication, such as how these processes are coordinated with the chromatin modifying enzymes and the spatiotemporal pattern of origin positioning and firing, etc.

In mammals, replication origins tend to cluster in the transcription promoter regions; therefore, transcription and replication are often co‐regulated.^[^ ^69^ ^]^ However, cell failures to prevent DNA replication initiation in transcribed regions leads to TRCs and subsequent DNA damage.^[^ ^52^ ^]^ We found that high‐ETV4 expression leads to R‐loop formation and DNA damage in response to TOP1 inhibitor CPT induced replication stress. However, those cells do not exhibit significant cellular DNA damage at undisturbed conditions, suggesting that ETV4 might use some safeguarding mechanisms to avoid inherent replication stress. Growing evidence indicates that MYC mitigates replication stress by induction of homologous recombination repair, upregulation of replication factors (MCM10, WEE1, etc.), cooperation with fork remodelers, and fine‐tuning of RNA polymerase dynamics.^[^ ^70^ ^]^ It was recently reported that MYC‐dysregulated cancers have an increased dependence on regulators of R‐loop formation to preserve genomic stability, and this vulnerability can be exploited by inhibiting TOP1, a regulator of R‐loop by DNA topology. These findings highlight that MYC level might be the predictive biomarker to identify cancers sensitive to TOP1 inhibitors.^[^ ^71^ ^]^ We found a significant correlation between ETV4 expression and R‐loop‐induced DSBs in NSCLC cells upon CPT treatment. Knocking down ETV4 reduced R‐loop burden and γ‐H2AX level, whereas overexpression of ETV4 led to the intolerable accumulation of R‐loops and DSBs following CPT treatment. CPT is known to induce replication‐associated and transcription‐dependent DSBs.^[^ ^57^ ^]^ Inhibiting transcription by DRB indeed reversed CPT‐induced R‐loop formation and γ‐H2AX level, which confirmed the transcription‐dependent replication stress induced by CPT treatment in NSCLC cells. Furthermore, in vitro experimental results demonstrate the enhanced efficacy of CPT on cell survival in the context of ETV4‐overexpressed lung cancers. Our data indicate that ETV4 is located at the origin‐promoter locus like the MCM4 gene, and its overexpression is more sensitive to TOP1 poisoning by replication‐ and transcription‐mediated R‐loop accumulation, DNA damage, and cell death.

In summary, the preponderant oncogenic ETS factor ETV4 exerts a pleiotropic control over DNA replication both in a transcription‐dependent and ‐independent fashion in NSCLC cells. High‐ETV4 expression leads to R‐loop formation and DNA damage in response to TOP1 inhibition. As directly targeting ETV4 is challenging, our findings present an exciting alternative strategy to exploit TOP1 as novel targets in ETV4‐dysregulated lung cancers. Future work should further investigate the benefit of TOP1 and other inhibitors in treating lung cancer addicted to ETV4‐overexpressed transcription and replication across panels of cell lines, patient‐derived organoids, and in vivo experimental test.

## 4 Experimental Section

### Cell Culture

A549, H1299, H358, and H1703 human NSCLC cell lines and HEK293T cells were obtained from the China Infrastructure of Cell Line Resources. H358T termed as EMT‐ed H358 cells treated with transforming growth factor (TGF)‐β showed high expression of ETV4 as compared with H358 cells.^[^ ^25^ ^]^ Cells were cultured in Roswell Park Memorial Institute (RPMI)‐1640 medium or Dulbecco's modified eagle medium (DMEM) supplemented with 10% fetal bovine serum and penicillin/streptomycin. Cultures were maintained at 37 °C in a 5% CO_2_ incubator. Cell synchronization was done using a thymidine block and released for G1/S, S, and G2 phase samples.

### siRNA Transfection, Plasmid Construction, and Transfection, Generation of Stable Cells

The siRNAs specifically targeting ETV4, MCM2, MCM4, MCM5, MCM10, ORC1, ORC6, SUPT16H, SSRP1, and control siRNA were synthesized by GenePharma (Shanghai, China). The siRNA sequences can be found in Table S3 (Supporting Information). Sh‐MCM2, sh‐MCM4, sh‐MCM5, sh‐MCM10, sh‐ORC1 lentiviral vectors (LV3, Puro) or negative control shRNA were kindly obtained from the human‐shRNA‐library of Tsinghua University (Beijing, China). Sh‐ETV4 lentiviral vectors (LV3, Puro) or negative control shRNA were obtained from GenePharma (Shanghai, China). The full‐length (EX‐T8074‐M11, neomycin) of ETV4 plasmids were obtained from GeneCopoeia (Guangzhou, China). Transfections were performed using Lipofectamine 2000 reagent (Invitrogen, Grand Island, NY) following the manufacturer's protocol. H358 cells transfected with ETV4 or control vectors were selected with neomycin to obtain stably overexpressing ETV4 (H358‐ETV4 cells) or control clones (H358 control cells). A549 cells transfected with MCM2, ‐4, ‐5, ‐10, ORC1, or control shRNA vectors were selected with puromycin to obtain stably gene‐depleting clones. Besides, H358‐ETV4 cells were transfected with MCM2, ‐4, ‐5, ‐10, ORC1, or control shRNA vectors selected with both neomycin and puromycin to obtain ETV4 + sh‐Ctrl, ETV4 + sh‐MCMs/ORC1 stable cells.

### Chromatin Immunoprecipitation (ChIP) and ChIP‐Sequence (ChIP‐seq)

The interaction of the ETV4 with the MCMs or ORC1 gene promoter and replication origins was analyzed using the ChIP Assay Kit (Merck Millipore, USA) according to the instruction manual provided by the manufacturer. Briefly, NSCLC cells were subjected to 1% formaldehyde incubation for 10 min to cross‐link DNA and its interacting proteins. The cross‐linked DNA‐protein complexes were then sheared into ≈500 bp DNA fragments using shearing by sonication, immunoprecipitated by a Flag antibody (14793, CST) for exogenous ETV4 detection, ETV4 antibody (sc‐113x, Santa Cruz), ORC1 (NBP100‐121, Novus), ORC6 (sc‐32735, Santa Cruz) for endogenous detection, or IgG (2729, CST) as a control. After the cross‐links are reversed, the precipitated chromatin DNA is eluted, followed by (q)PCR analysis. Primers used for MCMs and ORC1 gene promoter ChIP‐(q)PCR assays and for MCM4, LAMB2, and other origins binding assays were listed in Table S3 (Supporting Information). For anti‐Flag (ETV4) ChIP‐seq analysis, purified ChIP DNA from A549‐shETV4 cells transfected with Flag‐ETV4 was used for library construction following the Illumina ChIP–seq library generation protocol. Sequencing and data analysis were carried out by LC‐Bio Technology co.ltd., (Hangzhou, China) (GSE228833). In addition, ETV4 ChIP‐seq (ENCSR537FDJ, HepG2) and ATAC‐seq (ENCSR220ASC, A549) data were analyzed using a publicly available pipeline from The Encyclopedia of DNA Elements (ENCODE).

### Western Blot Analysis

Identical quantities of proteins extracted from NSCLC cells were analyzed by sodium dodecyl sulfate‐polyacrylamide gel electrophoresis (SDS‐PAGE) and transferred electrophoretically to the nitrocellulose membrane. Blots were blocked with 5% nonfat milk in Tris‐Buffered Saline and Tween 20 (TBST), and incubated with antibodies specific for ETV4 (10684‐1‐AP, Proteintech), MCM2 (3619, CST), MCM3 (15597‐1‐AP, Proteintech), MCM4 (13043‐1‐AP, Proteintech), MCM5 (11703‐1‐AP, Proteintech), MCM6 (13347‐2‐AP, Proteintech), MCM7 (11225‐1‐AP, Proteintech), MCM10 (12251‐1‐AP, Proteintech), ORC1 (NBP100‐121, Novus), ORC2 (12739‐1‐AP, Proteintech), ORC3 (sc‐374231, Santa Cruz), ORC4 (13026‐1‐AP, Proteintech), ORC5 (11542‐1‐AP, Proteintech), ORC6 (17784‐1‐AP, Proteintech), Histone‐H3 (17168‐1‐AP, Proteintech), PCNA (13110, CST), phospho‐MCM2^S40^ (ab133243, Abcam), CDC45 (15678‐1‐AP, Proteintech), SUPT16H (28598‐1‐AP, Proteintech), SSRP1 (15696‐1‐AP, Proteintech), γ‐H2AX (ET‐1602, HUABio), Flag (14793, CST), His (66005‐1‐Ig, Proteintech), HA (sc‐7392, Santa Cruz), GST (66001‐2‐lg, Proteintech), GAPDH (60004‐1‐Ig, Proteintech) and β‐actin (66009‐1‐Ig, Proteintech) was used as a loading control for Western blot. The blots were then re‐probed with a secondary antibody, visualized by the chemiluminescence and scanned using the ImageQuant LAS 4010 Imaging System (GE Healthcare Life Sciences, Piscataway, NJ).

### Co‐Immunoprecipitation (Co‐IP)

Cells were washed twice with PBS and lysed in ice‐cold RIPA lysis buffer (3201‐2, BestBio) supplemented with protease inhibitors for 30 min on ice and isolated by centrifugation 16100 × g for 10 min. Supernatants were transferred to new tubes, 1/10 of the sample was kept as input control, while the remaining lysates were incubated with antibodies against ETV4 antibody (sc‐113, Santa Cruz), Flag (14793, CST), His (66005‐1‐lg, Proteintech), HA (sc‐7392, Santa Cruz), SUPT16H (28598‐1‐AP, Proteintech), SSRP1 (15696‐1‐AP, Proteintech), MCM2 (3619, CST), or IgG (2729, CST) at 4 °C overnight with gentle agitation, followed by incubation with Protein A/G Plus‐Agarose (sc‐2003, Santa Cruz) at 4 °C for 2 h. Beads were washed more than three times with lysis buffer, and the immunoprecipitated proteins were eluted and denatured with 2 × Laemmli buffer and boiled for 10 min at 100°C, separated by SDS‐PAGE.

### His Pull‐Down Assay

Pull‐down assays were performed either from cellular extracts or from mixtures of purified recombinant proteins in vitro. His‐ETV4 (TP760035, Origene) and His‐histone H3 (TP761054, Origene) proteins were expressed, purified, and adjusted to 5 µg µL^−1^ as the final concentration. A549 or H1299 (1 × 10^7^) cells were lysed with 2 mL RIPA lysis buffer, and 200 µL was used as input. The remaining lysates were incubated with 20 µg of histone H3 or ETV4 combined Ni‐NTA His‐bind resin column at 4 °C for 6 h. Target proteins were further detected by Western blot. In addition, histone H3 (without tag, HY‐72332, MCE), Flag‐tag MCM2 (1‐440aa, Novoprotein), GST‐tag SUPT16H (1‐510aa, Novoprotein), and Flag‐tag SSRP1 (TP300414, Origene) proteins were expressed and purified, mixed with 10 µg of His‐ETV4 and incubated with Ni‐NTA agarose (Beyotime, China), respectively, for the His‐tag pull‐down assays. The pellets were washed three times and boiled in SDS loading buffer. The bound proteins were loaded onto an SDS‐polyacrylamide gel and examined by western blot with the anti‐tag antibody.

### Cell Fractionation and Analysis of Nuclear Fractions

For subcellular fractionation, cells in the mid‐exponential phase of growth were collected by scraping from the culture dish after two washes with ice‐cold phosphate‐buffered saline (PBS). Subcellular fractions were prepared using the Minute TM Cytoplasmic and Nuclear Fraction kit (SC‐003, Invent Biotechnologies). The nuclear fraction was subjected to further fractionation by 15 min incubation on ice with 200 µL of ice‐cold CSK buffer (10 mM PIPES, pH 6.8, 100 mM NaCl, 300 mM sucrose, 1 mm MgCl_2_, 1 mM EGTA, 1 mM DTT, 1 × Cocktail) containing 0.5% Triton X‐100. Chromatin‐bound and unbound proteins were separated by centrifugation (3 000 rpm, 5 min at 4 °C). The recovered precipitate was washed once with CSK buffer containing 0.5% Triton X‐100 and suspended in the above buffer to obtain chromatin‐bound proteins (Chrom.). The supernatants were then clarified by centrifugation at 18,800 × g for 15 min at 4 °C to constitute the chromatin‐unbound proteins (soluble fraction (Sol.)) For each fraction, protein amounts derived from the comparable number of cells were analyzed by SDS‐PAGE and Western blot. Histone H3 and PCNA were used as a loading control for Chrom. and Sol. proteins, respectively.

### Flow Cytometry Analysis

Cells were collected and washed twice with cold PBS. For cell cycle analysis, cells were fixed in 70% ethanol at 4 °C overnight. After centrifugation for 5 min at 1 000 rpm at 4 °C, the pellet was treated with 2 mg mL^−1^ RNase A at 37 °C for 20 min and stained with 50 µg mL^−1^ propidium iodide (PI) containing 0.1% Triton X‐100 and EDTA 0.02 mg mL^−1^. Cell suspensions were analyzed by flow cytometry on a FACS Calibur system (BD Biosciences, Heidelberg, Germany). Cell cycle distribution was counted using Multicycle AV software.

### Proximity Ligation Assay (PLA)

PLA was performed using Duolink® Proximity Ligation Assay (DUO92101, Sigma) following the manufacturer's protocol. Briefly, a total of 1 × 10^6^ cells were seeded overnight in a six‐well plate. The next day, cells were collected, fixed in 4% paraformaldehyde solution, permeabilized with 0.5% Triton‐X100, blocked with Duolink® Blocking Solution, and probed with antibodies directed against ETV4 (sc‐113, Santa Cruz) and MCM2 (3619, CST) or SUPT16H (28598‐1‐AP, Proteintech) at 4 °C overnight. In Situ PLA probes anti‐mouse plus and anti‐rabbit minus were diluted 1:5 and incubated for 1 h at 37 °C. Cells were washed in Buffer A solution three times for 5 min each. The ligation reaction was incubated at 37 °C for 30 min. Cells were washed in buffer A twice for 2 min each. Amplification reactions were incubated at 37 °C for 100 min. Cells were washed in wash buffer B solution three times for 10 min each and one time in 0.01 × diluted wash Buffer B solution for 1 min. Cells were mounted with mounting media containing DAPI. Images were taken using a Lecia Laser Confocal Microscope (Leica, German) with 60 × objective oil immersion.

### Prediction of Protein–Protein Interaction

The 3D structures of proteins were obtained from RCSB PDB (Protein Data Base, https://www.rcsb.org/). ORC complex from PDBID: 7MCA, ETV4 from PDBID: 4CO8 and 4UUV, and MCM complex from PDBID: 5BK4. There were no complete structures of MCM2 and MCM10. Thus we downloaded their structures from UniProt (https://www.uniprot.org/) predicted by AlpaFold. Then, we conducted protein docking for protein–protein interaction using GRAMM (https://gramm.compbio.ku. edu/). Finally, all the structures were exported as pictures using PyMOL software.

### EdU Assay for Confocal Microscope Test

EdU (5‐ethynyl‐2’‐deoxyuridine), a nucleoside analog of thymidine, is readily incorporated into cellular DNA during DNA replication. Cell proliferation was evaluated using a Cell‐Light EdU Apollo 488 In Vitro Imaging Kit (Ribobio, Guangzhou, China) as described by the manufacturer. Briefly, cells were previously seeded onto coverslips and incubated overnight. 48 h post‐transfection, cells were incubated with 50 µM EdU for 2 h at 37 °C, fixed with 4% formaldehyde, then stained with Apollo reaction cocktail and Hoechst 33342, protected from light. Images were acquired with a Lecia Laser Confocal Microscope (Leica, German).

### RNA Extraction, cDNA Synthesis, and RT‐qPCR

Total RNA from cultured cells was extracted using TRIzol reagent (Invitrogen). The first‐strand cDNA was first synthesized from 1 µg RNA using GoScript™ Reverse Transcription System (Promega, China), followed by PCR amplification using 5% cDNA for each reaction. The ACTB gene (β‐actin) was used as an internal control. The expression of each target, including ETV4, MCM2, ‐4, ‐5, ‐10, and ORC1, was calculated using the 2^−ΔΔCt^ method and presented as relative mRNA expression. Experiments were repeated at least three times. The respective primers can be found in Table S3 (Supporting Information).

### Quantitative In Situ Analysis of Protein Interactions at DNA Replication Forks (SIRF)

SIRF technology allows for the quantitative assessment of protein interactions with DNA replication forks using in situ Proximity Ligation Assay (PLA) technology. SIRF was performed as previously described.^[^ ^46^ ^]^ Cells grown in log‐phase were plated the day before the experiment at 50–60% confluence onto coverslips. Cells were incubated with 125 µM EdU in growth media for 15 min at 37°C. To perform a thymidine chase, EdU was removed, and cells were washed twice with media before adding media with 100 µM thymidine. After EdU pulse and/or thymidine chase, decant media, cells were immediately fixed with 4% formaldehyde in PBS (pH 7.4) for 15 min at room‐temperature. After fixation, cells were permeabilized, incubated with click reaction solution, and blocked as described in EdU‐labeling combined with Immunostaining. Rabbit anti‐human primary antibodies for ETV4 (HPA005768, ATLAS); MCM2 (10684‐1‐AP, Proteintech); SSRP1(15696‐1‐AP, Proteintech), SUPT16H (28598‐1‐AP, Proteintech); and mouse anti‐human primary antibody for Biotin (1:200; 03–3700, Invitrogen) were incubated at 4 °C overnight in a humidified chamber. Cells were washed three times with wash buffer A for 5 min each. PLA was performed using Duolink® Proximity Ligation Assay (DUO92101, Sigma) following the manufacturer's protocol, as described in the PLA assay. EdU was detected by incubation with FITC‐conjugated anti‐mouse antibody (115‐095‐166, Jackson ImmunoReaserch) for 1 h at room‐temperature, washed with PBS three times for 5 min each, and once in 0.01 × diluted wash buffer B solution for 1 min. Cells were mounted with mounting media containing DAPI. Images were taken using a Lecia Laser Confocal Microscope (Leica, German) with 60 × objective oil immersion. The results of SIRF experiments were quantified by counting the number of PLA foci and PLA/EdU foci per nucleus in NSCLC cells.

### Isolation of Proteins on Nascent DNA (iPOND)

iPOND was performed as described previously.^[^ ^15^ ^]^ Briefly, NSCLC cells were synchronized by thymidine block and released into the S phase. 1 × 10^8^ cells per condition were labeled with 10 µM EdU for 1 h. Cells were fixed with 1% formaldehyde for 20 min and quenched with 1.25 M glycine. After permeabilization with 0.25% Triton, the click reaction was performed at room‐temperature for 1 h in a buffer containing 2 mM copper sulfate, 10 µM biotin‐azide, and 10 mM sodium ascorbate added to PBS. For negative control samples, DMSO was used instead of biotin azide. Cells were then lysed in RIPA buffer supplemented with phosphatase and protease inhibitors and sonicated to make the lysate clear/opaque. After centrifugation at 13 000 rpm for 10 min, a 5% lysate volume was set aside as input. Biotin‐labeled EdU was captured by incubating samples with streptavidin‐coupled agarose beads (Beyotime, China) for 20 h. Beads were washed once with lysis buffer, once with 1 M NaCl, and twice with lysis buffer. Cross‐link reversal and elution of immunoprecipitated proteins were achieved by heating beads in 2 × SDS Laemmli sample buffer containing 0.2 M dithiothreitol (DTT) for 25 min at 95 °C. The eluted proteins were analyzed using Western blot. PCNA was used as a positive control within each experiment to ensure the procedure worked as expected.

### Chromatin Assembly Assay (CAA)

CAA was performed as previously described.^[^ ^49^, ^50^ ^]^ The detailed information is similar to the SIRF assay, including pulse‐labeled with 125 µm EdU, fixed with 4% formaldehyde, permeabilized by 0.3% Triton, subjected to Click‐iT reaction with biotin‐azide, PLA reactions between the anti‐biotin antibody and anti‐Acetyl‐lysine (105RM, PTM BIO), anti‐acetyl‐histone H3 (Lys27) (116, PTM BIO), anti‐Pan Kla (1401RM, PTM BIO), or anti‐lactylated H3 (Lys18) (1406RM, PTM BIO) were performed as described in the PLA assay. Following PLA, cells were immunostained with FITC‐conjugated anti‐mouse antibody (115‐095‐166, Jackson ImmunoReaserch) to detect EdU to control the specificity of CAA. The results of CAA experiments were quantified by counting the number of PLA foci and PLA/EdU foci per nucleus in NSCLC cells.

### Luciferase Reporter Assay

To create gene promoter luciferase reporter constructs, ≈1–1.5Kb fragments of promoter DNA were synthesized and cloned into the pEZX‐PL01 luciferase reporter vector (GeneCopoeia, Guangzhou, China). The plasmid of pEZX‐PL01‐MCM4 was obtained from GeneCopoeia (Guangzhou, China), while the other plasmids were constructed in our lab. The primers used for Luciferase reporter construction can be found in Table S3 (Supporting Information). The amplified fragment was cloned into the pEZX‐PL01 vector at MluI and XbaI (or BamHI) sites. In addition, mutations in the putative ETV4‐binding sites (mut‐1 or mut‐2 type) of MCM2, ‐4, ‐5, ‐10, or ORC1 promoter region were sub‐cloned into the pEZX‐PL01 luciferase reporter vector to construct mutant luciferase reporter plasmids (Genscript, Nanjing, China). All the plasmid sequences were confirmed by DNA sequencing. The wild‐type luciferase reporter plasmids were co‐transfected with the ETV4 plasmid, the ETV4‐DBD deletion plasmid, or the empty vector into HEK293T cells. The constructed luc‐MCMs (ORC1) ‐mut‐1 or luc‐MCMs (ORC1) ‐mut‐2 type luciferase reporter plasmids were co‐transfected along with the ETV4 plasmid or the empty vector into HEK293T cells. The luciferase activity was measured using the Luc‐Pair™ Duo‐Luciferase HS Assay Kit (GeneCopoeia) and normalized to Renilla luciferase activity. Experiments were repeated at least thrice.

### Animal Studies

The animal studies were approved by the Committee on Ethics of Biomedicine, Hebei Medical University (Protocol # IACUC‐Hebmu‐2023017). 5 weeks old female athymic BALB/c nude mice (Vital River, Beijing, China) were used for animal studies. In total 5 × 10^6^ cells were subcutaneously injected into the right flanks of mice, tumor growth was weekly recorded with a caliper, and the tumor volume was calculated as a × b^2^ × 0.5 (a, longest diameter; b, shortest diameter). The tumors were allowed to grow for varied times when the volume of tumors in a certain group reached ≈1 000 mm^3^. For A549 cells stably MCM2, ‐4, ‐5, ‐10, and ORC1 depleting or sh‐control cells were allowed to grow for 5 weeks. For H358 cells stably ETV4 overexpressing cells and control cells were allowed to grow for 3 weeks. For the H358‐ETV4 + sh‐control cells, H358‐ETV4 + sh‐MCMs/ORC1 cells, the tumors were allowed to grow for 3 weeks when the volume of tumors in the H358‐ETV4 + sh‐control cells group reached ≈1000 mm^3^. The mice were euthanized, and tumors were excised, weighed, harvested, fixed, and embedded. The average weight of tumors was calculated and compared between the groups. Tumor tissue specimens were also stained with hematoxylin and eosin for histological study.

### Patients and Clinical Specimens

The study protocol was approved by the Institutional Review Boards of Hebei Medical University. Formalin‐fixed and paraffin‐embedded (FFPE) sections of 81 patients with lung cancer (37 lung squamous cell carcinomas, LUSCs; and 44 lung adenocarcinomas, LUADs) were collected from the Second and Fourth Hospital of Hebei Medical University between 2010 and 2013. The clinical characteristics of the patients are shown in Tables S1 and S2 (Supporting Information). None of the patients had received preoperative adjuvant chemotherapy or radiotherapy. Survival time was defined as the period from the date of primary surgery to the date of death by 2019.

### Immunohistochemistry Assay

Paraffin‐embedded lung tissues were incubated with the following antibodies: anti‐ETV4 antibody (10684‐1‐AP, Proteintech), anti‐MCM2 antibody (3619, CST), anti‐MCM4 antibody (13043‐1‐AP, Proteintech), anti‐MCM5 antibody (11703‐2‐AP, Proteintech), anti‐MCM10 antibody (12251‐1‐AP, Proteintech), and anti‐ORC1 antibody (NB100‐121, Novus). Immunohistochemical scoring of nuclear staining of the above proteins was performed as follows: positive nuclear expression in over 50% of the tumor cells was scored as positive, while cytoplasmic expression and nuclear expression in < 50% of the tumor cells were scored as negative. Quantification of the four proteins’ expression was performed blindly by a pathologist.

### Cell Proliferation Assays

4 × 10^3^ cells were seeded onto 96‐well plates in standard culture conditions overnight, followed by siRNA or plasmid transfection. The cell viability was assessed in four to eight replicates at 24, 48, 72, and 96 h after transfection by MTT assay. The experiments were performed at least three times.

### Clonogenic Assay

NSCLC cells were seeded at a 6‐well plate 48 h after ETV4 knockdown or overexpression. Cells were treated with 0, 0.1, or 1µM CPT for 24 h. 14 days after treatment, colonies were fixed in 100% methanol for 10 min and stained using 0.5% crystal violet and 20% methanol for 10 min. The number of colonies was counted and normalized to the CPT‐untreated control. All experiments were conducted in triplicate replicates.

### γ‐H2AX or MDC1 Foci Assay by Immunofluorescence

4.0 × 10^4^ cells were seeded onto coverslips 24 h before siRNA was added. 48 h after transfection, cells were treated with CPT (25 µM, 30 min) and/or DRB (100 µM, 3 h). After fixed for 15 min in 4% formaldehyde, cells were washed in PBS, permeabilized for 5 min in PBS containing 0.5% Triton‐X100, washed twice in PBS, blocked for 1 h in 3% BSA/0.2% Tween‐20 in PBS, followed by immunostaining with anti‐γ‐H2AX (NBP100‐121, HUABio) or MDC1 (24721‐1‐AP, Proteintech) antibody at 4 °C overnight. After washing twice with 1 × PBS, the cells were incubated with secondary antibodies for 1 h at room‐temperature, washed with 1x PBS twice, and finally mounted with Antifade DAPI (DUO82040, Sigma–Aldrich). Images were obtained using a Lecia Laser Confocal Microscope (Leica, German). The intensity of γ‐H2AX and MDC1 were quantified by counting the number of foci per nucleus in NSCLC cells.

### Dot Blot Assay

After ETV4 knockdown or overexpression for 48 h, cells were treated with CPT (25 µM, 30 min) and/or DRB (100 µM, 3h). Total nucleic acid from cultured NSCLC cells was extracted using a DNA extraction kit (DP304, Tiangen Biotech). Indicated amounts of purified nucleic acid were applied to the Hybond N+/positive nylon membranes (RPN303B, Cytiva). The membrane was subsequently UV cross‐linked and blocked with 5% nonfat milk in TBST for 1 h at room‐temperature. The level of R‐loop was detected by incubation with mouse monoclonal S9.6 antibody (ZMS1017, Sigma–Aldrich) overnight at 4°C and with secondary antibodies at 37 °C for 1 h after three washes in TBST. The bolts were visualized by the chemiluminescence and scanned using ImageQuant LAS 4010 Imaging System (GE Healthcare Life Sciences, Piscataway, NJ).

### DNA‐RNA Immunoprecipitation (DRIP)‐qPCR

DRIP assays were performed as described previously with modification.^[^ ^59^ ^]^ Briefly, 5 × 10^6^ cells were lysed in DRIP lysis buffer (TE, 0.5% SDS) and incubated with 62.5 ng mL^−1^ Proteinase K overnight at 37 °C. Then, Phenol:Chloroform:Isoamyl Alcohol was added to the mixture and, once separated de aqueous phase, genomic DNA was precipitated by adding 1/10 volume 3 M NaOAc and 1 volume isopropanol. The harvested DNA was digested at 37 °C for 24 h using cocktail restriction enzymes (30 units/100 µg DNA, each of BsrGI, EcoRI, HindIII, SspI, and XbaI) according to supplier instructions (New England Biolabs). Digested DNAs were cleaned up by pheno‐chloroform extraction. Treat the half of the DNA (one tube) with RNase H (20 units/100 µg DNA, NEB) overnight at 37 °C. In the meantime keep the other half at 4 °C. After purification, DNA concentration was checked, and 1 µg of DNA of treated and untreated with RNase H samples was used as input. RNA: DNA hybrids from 4 µg digested DNA (treated or not with RNase H) were immunoprecipitated using 5 µg of S9.6 antibody (65983, Active Motif) and 50 µL of protein A/G agarose beads at 4 °C overnight in IP buffer (10 mM NaPO4, 140 mM NaCl, 0.05% Triton X‐100). The beads were then washed four times with IP buffer for 10 min at 4 °C, and the nucleic acids were eluted with elution buffer (50 mM Tris‐HCl, pH8.0, 10 mM EDTA, 0.5% SDS, and 1 µg µL^−1^ protease K) at 55 °C for 1 h. Immunoprecipitated DNA was then cleaned up by a phenol‐chloroform extraction followed by ethanol precipitation at −20 °C for 1 h. Quantitative PCR was performed at the indicated regions using the primers listed in Table S3 (Supporting Information). All PCR reactions were performed in triplicate. Enrichment of RNA:DNA hybrids is calculated as percentage of input.

### Microarray Analysis

Arraystar Human LncRNA Microarray V4.0 was used to screen the global profiling of human long‐noncoding RNAs (lncRNAs) and protein‐coding transcripts (mRNA) in H1299, H1703, and H358T NSCLC cells transfected with ETV4 siRNA or negative control siRNA as described (GSE137445). The threshold we used to screen the upregulated or downregulated mRNA/lncRNAs was a fold change ≥ 2.0 and a P‐value ≤ 0.05.

### Statistical Analysis

Data are presented as mean ± standard deviation (SD). *t*‐test, one‐way analysis of variance (ANOVA), two‐way ANOVA, the extra‐sum‐of‐squares F test, and Kaplan‐Meier survival analysis were performed using the GraphPad Prism Software. The χ2 test was analyzed using computer‐aided SPSS (Chicago, IL) 13.0 statistical software. A value of *p* < 0.05 was considered significant. The statistical parameters are specified within the figure legends.

## Conflict of Interest

The authors declare no conflict of interest.

## Author Contributions

J.Z. and Y.W. contributed equally to this work. J.Z. and Y.W. contributed to IHC, in vitro, and in vivo analyses. S.C. performed the prediction of protein–protein interaction and data analysis. S.M.X. participated in writing and revising the manuscript. B.L. and Y.L. helped to perform the animal studies. Y.H., X.M., M.R., D.B., J.K. and R.L. helped to perform in vitro assay, FCM analysis, and IF tests. L.L. and J.W. reviewed the slides of all human NSCLC cases. L.X. designed the experiments and wrote the manuscript. All authors have read and approved the final manuscript.

## Supporting information



Supporting Information

## Data Availability

The data that support the findings of this study are available in the supplementary material of this article.
