# Supplementary material for: Topoisomerase I Inhibition in ETV4‐overexpressed Non‐Small Cell Lung Cancer Promotes Replication and Transcription Mediated R‐Loop Accumulation and DNA Damage
Source: Adv Sci (Weinh). 2025 Jun 23;12(35):e09307. doi: 10.1002/advs.202409307 (PMC12462987; doi:10.1002/advs.202409307)
Supplement: Supplementary file 1 — Supporting Information [file ADVS-12-e09307-s001.docx]

Supporting Information for

**Topoisomerase I Inhibition in ETV4-overexpressed Non-Small Cell Lung Cancer Promotes Replication and Transcription Mediated R-Loop Accumulation and DNA Damage**

Jiaxi Zhang, Yan Wang, Shanhu Cao, Shelly M. Xie, Bei Liu, Yimeng Li, Yuqi Hou, Xue Meng, Mingzhu Ruan, Di Bu, Jia Kang, Ruxin Li, Lei Lou, Juan Wang, Lingxiao Xing*

J. Zhang, Y. Wang, S. M. Xie, B. Liu, Y. Li, Y. H, X. Meng, M. Ruan, D. Bu, J. Kang, R. Li, L. Xing

Department of Pathology, Hebei Medical University

Center of Metabolic Diseases and Cancer Research, Institute of Medical and Health Science, Hebei Medical University

Shijiazhuang, 050017, P. R. China.

E-mail: xinglingxiao@hebmu.edu.cn, or xinglingxiao@hotmail.com.

S. Cao

Department of Cardiology, The First Hospital of Hebei Medical University

Hebei Key Laboratory of Heart and Metabolism

Department of Biochemistry and Molecular Biology, Hebei Medical University, Shijiazhuang, 050017, P. R. China.

L. Lou, J. Wang

Department of Pathology, Second Hospital of Hebei Medical University

Shijiazhuang, 050017, P. R. China.

**Supporting Figures**

**
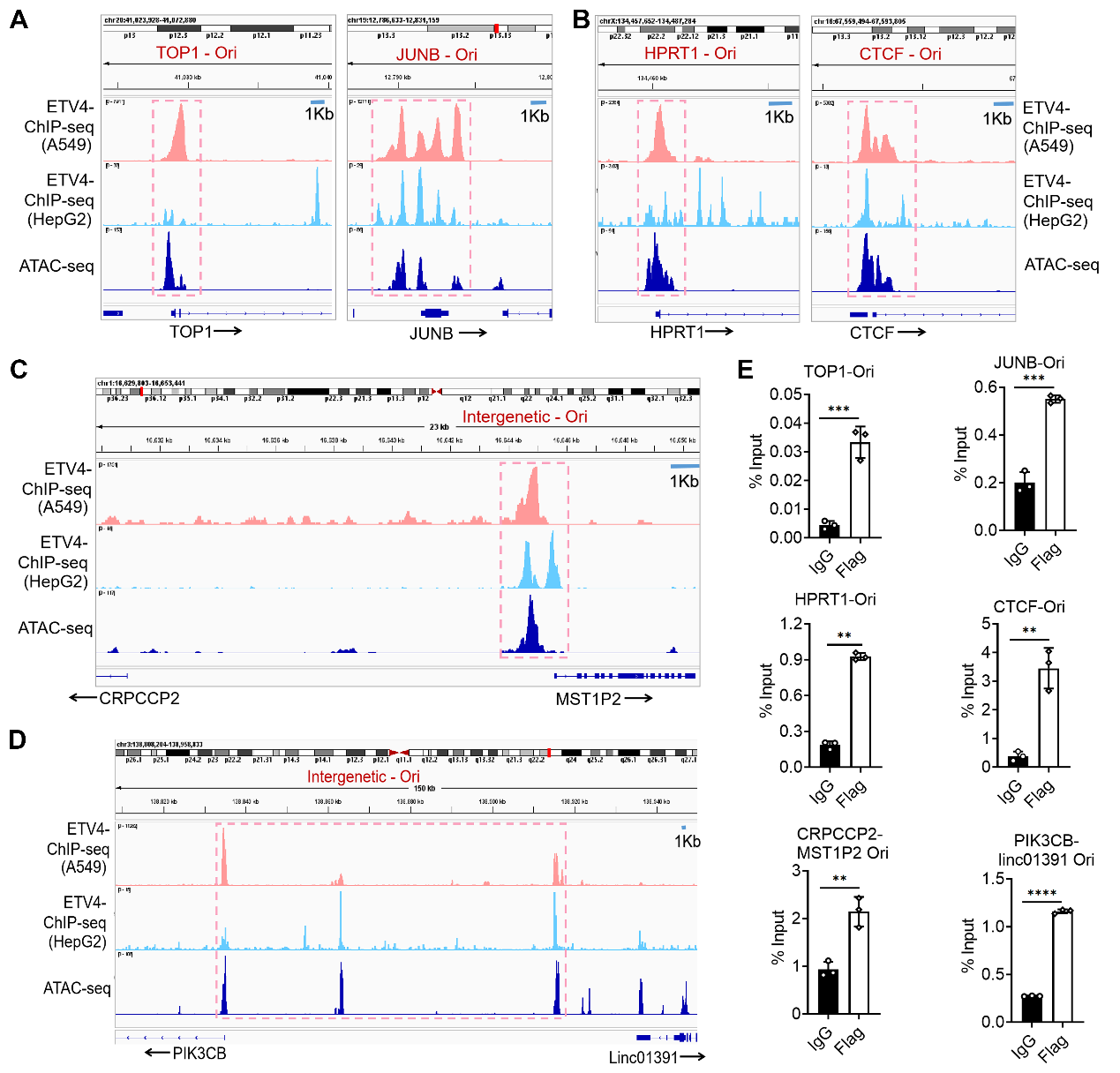
**

**Figure S1.** ETV4 is located at certain replication initiation sites. **(A-D)** IGV tracks showing the enrichment of ETV4 at specific human replication initiation loci such as TOP1, JUNB, HPRT1, CTCF, and two intergenic replication initiation sites (CRPCCP2-MST1P2 and PIK3CB-Linc01391 origins) by aligning the data obtained from our ETV4 ChIP-seq with that of ETV4 ChIP-seq (ENCSR537FDJ, HepG2) and ATAC-seq (from ENCSR220ASC, A549). **(E)** Anti-Flag-ChIP qPCR assay showing the binding of ETV4 at the above origin regions in HEK293T cells transfected with Flag-ETV4 plasmids (mean ± SD, n = 3; two-tailed unpaired *t*-test). ***P* < 0.01; ****P* < 0.001; *****P* < 0.0001;

**
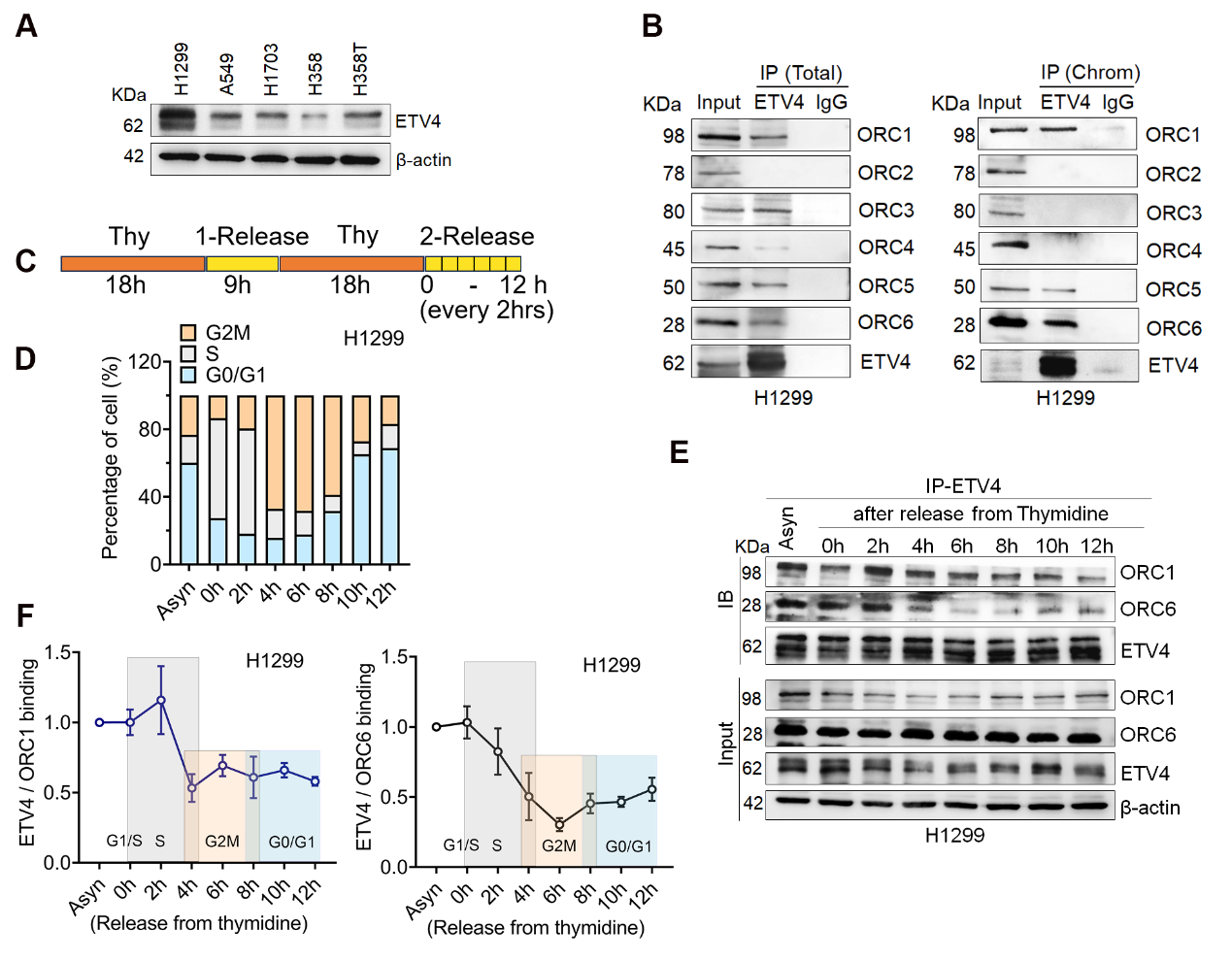
**

**Figure S2.** ETV4 interacts with ORC subunits in H1299 cells. **(A)** ETV4 protein expression in NSCLC cell lines used in the study was analyzed with Western blot assay. H358T is TGF-β-induced EMT-ed H358 cells with higher ETV4 expression than H358 cells. **(B)** IP of ETV4 and Immunoblots against ORC1-6 subunits from whole-cell proteins or chromatin-bound (Chrom.) proteins of H1299 cells. **(C)** Schematic of the protocol for cells synchronized and released using a double thymidine block. **(D)** The cell cycle distribution was detected by Flow cytometry. **(E)** IP of ETV4 and Immunoblots against ORC1 and ORC6 from thymidine block and released synchronized H1299 cells. **(F)** Quantification of ETV4-ORC1 or ORC6 binding in (E).

**
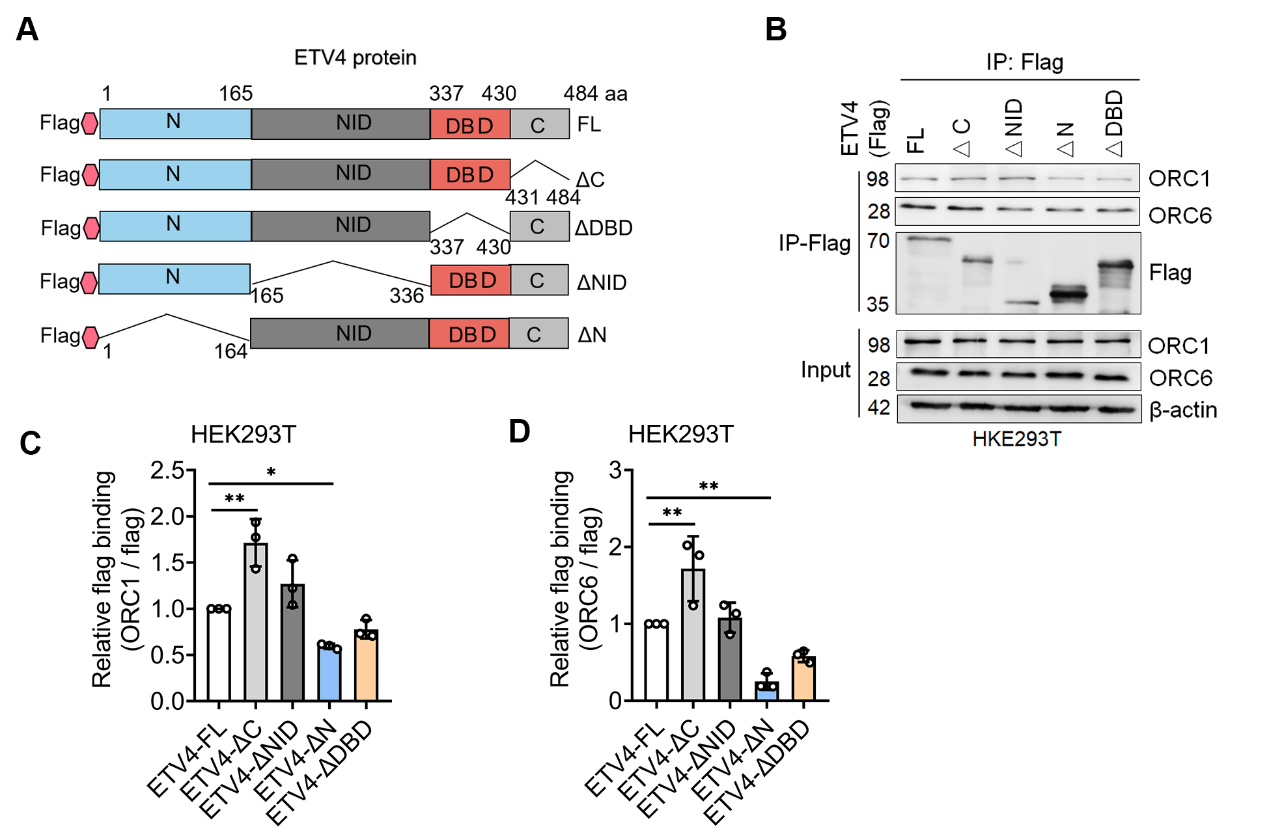
**

**Figure S3.** ETV4-DBD or N-terminal deletion on the interaction with ORC1/6 subunits. **(A)** Schematic representation of the N-terminally Flag-tagged ETV4 deletion constructs. **(B)** HEK293T cells were transfected with the indicated Flag-ETV4 vectors, and the lysates were subjected to anti-flag immunoprecipitation followed by immunoblot analysis with ORC1 and ORC6 antibodies. **(C, D)** Quantification of (B) (n = 3) presented as mean ± SD. Relative ETV4 binding with ORC1 and ORC6 was normalized with flag protein. One-way ANOVA, **P* < 0.05; ***P* < 0.01.

**
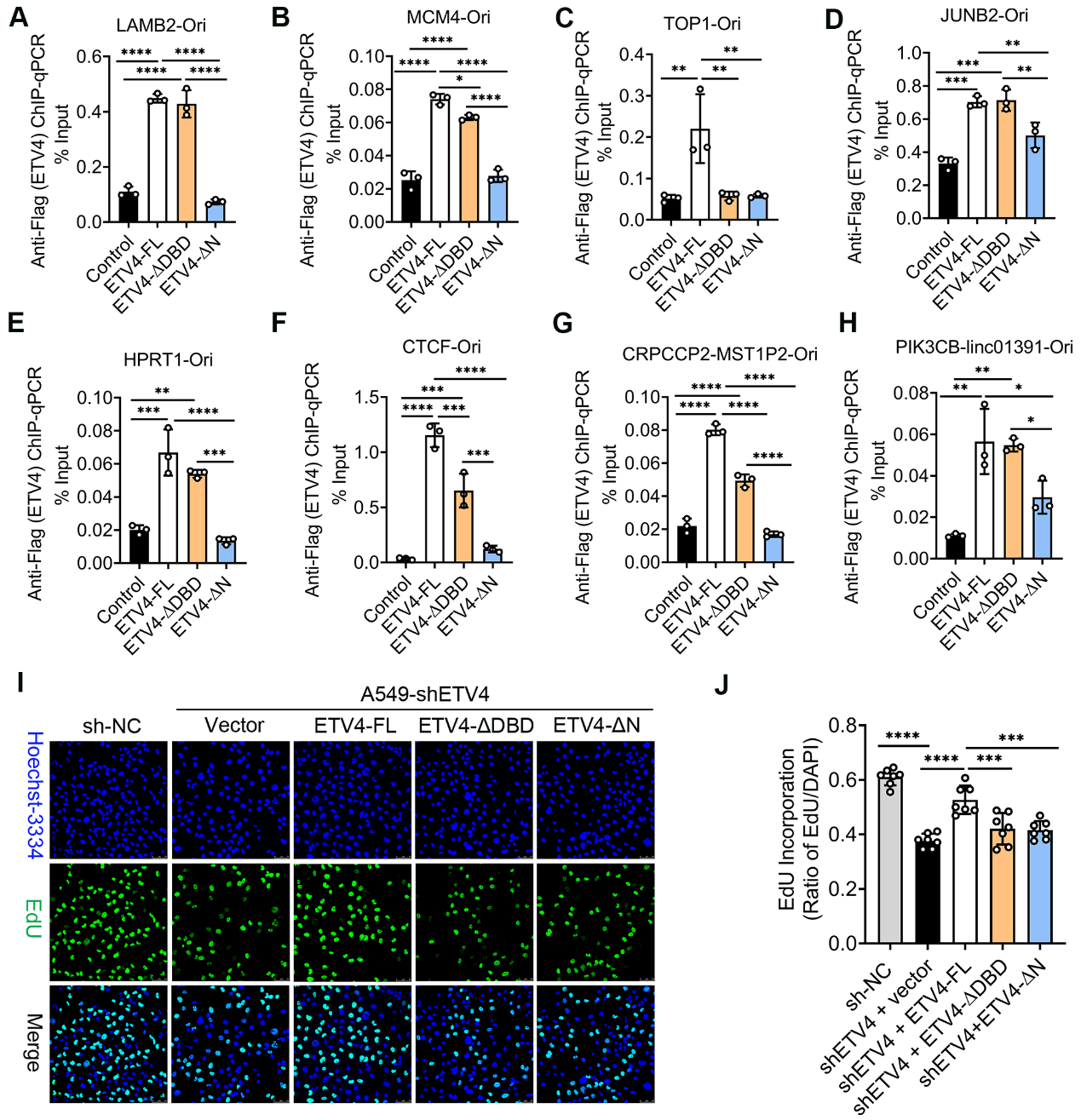
**

**Figure S4.** ETV4-DBD or N-terminal deletion on the recruitment of flag (ETV4) at DNA replication origins. **(A-H)** ETV4 N-terminal or DBD deletion on the recruitment of ETV4 protein at specific human replication initiation loci such as LAMB2, MCM4, TOP1, JUNB, HPRT1, CTCF, and two intergenic replication initiation sites (CRPCCP2-MST1P2 and PIK3CB-Linc01391 origins) in A549 sh-ETV4 cells transfected with control, the full-length Flag-tagged ETV4, ETV4-ΔDBD, or ETV4-ΔN plasmids, respectively. (mean ± SD, n = 3; One-way ANOVA followed by Tukey’s multiple comparisons test). **P* < 0.05; ***P* < 0.01; ****P* < 0.001; *****P* < 0.0001. **(I, J)** DNA replication was evaluated using EdU-incorporation assays in A549 sh-ETV4 cells transfected with control, the full-length Flag-tagged ETV4, ETV4-ΔDBD, or ETV4-ΔN plasmids, respectively. (mean ± SD; n = 7; One-way ANOVA followed by Tukey’s multiple comparisons test). ****P* < 0.001; *****P* < 0.0001.


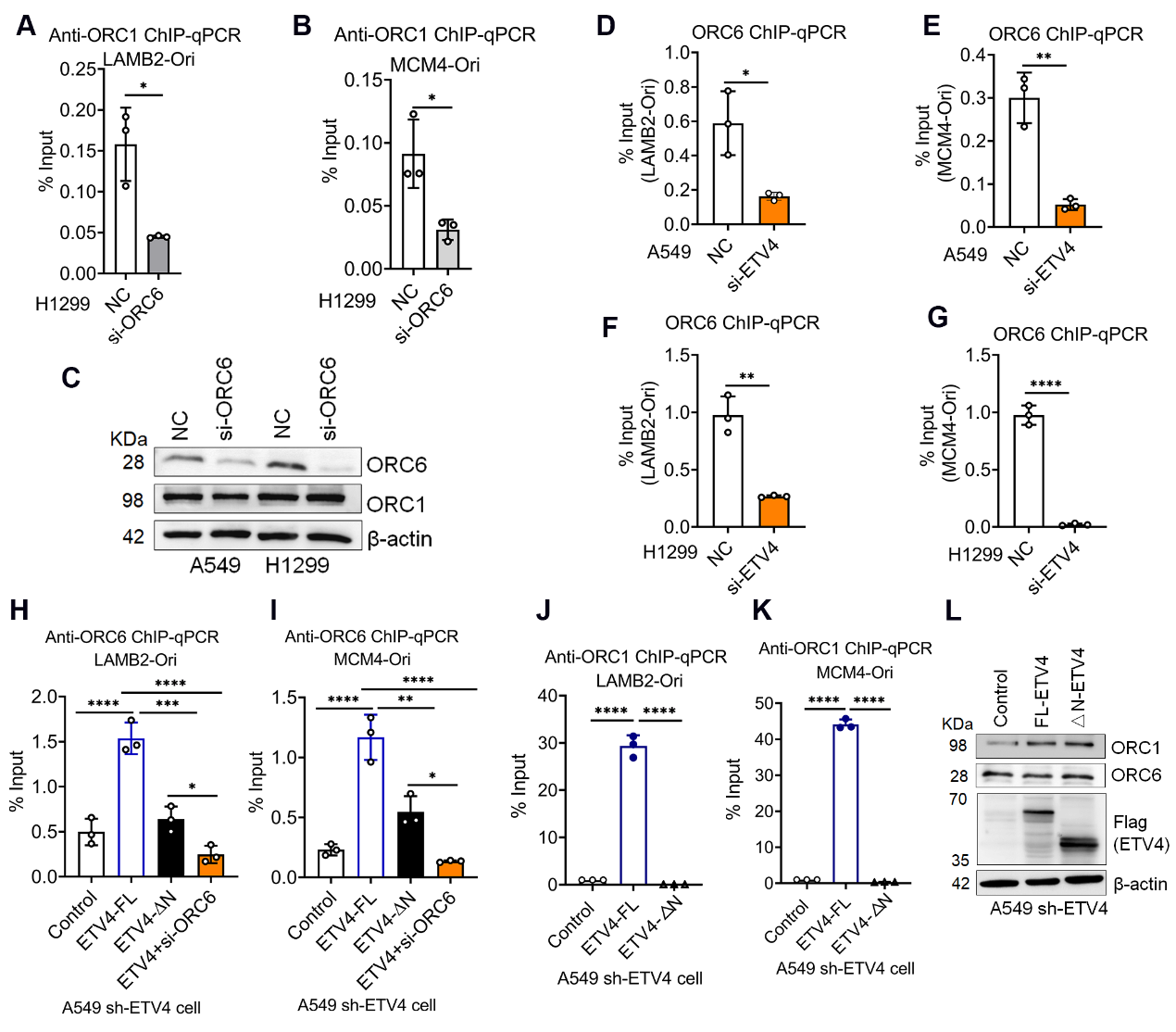


**Figure S5.** ETV4 and ORC6 on the recruitment of ORC at MCM4 and LAMB2 origin in NSCLC cells. **(A, B)** ChIP-qPCR assay showing the binding of ORC1 at LAMB2 and MCM4 origins in H1299 cells transfected with ORC6 siRNA or NC siRNA. (mean ± SD, n = 3; two-tailed unpaired *t-*test) **P* < 0.05. **(C)** siRNA-mediated ORC6 knockdown on the expression of ORC1 protein in A549 and H1299 cells using Western blot assay. **(D-G)** ChIP-qPCR results showing the recruitment of ORC6 at both origins in si-ETV4 knockdown A549 and H1299 cells. (mean ± SD, n = 3; two-tailed unpaired *t*-test) **P* < 0.05; ***P* < 0.01; *****P* < 0.0001. **(H, I)** A549 sh-ETV4 cells were transfected with the full-length Flag-tagged ETV4, ETV4-ΔN plasmids, or ETV4 + si-ORC6, and ChIP-qPCR was performed to show the binding of ORC6 at MCM4 and LAMB2 origins. (mean ± SD, n = 3; One-way ANOVA followed by Tukey’s multiple comparisons test). **P* < 0.05; ***P* < 0.01; ****P* < 0.001; *****P* < 0.0001. **(J, K)** ETV4-ΔN on the role of ETV4 recruiting of ORC1 at LAMB2 and MCM4 origins. (mean ± SD, n = 3; One-way ANOVA followed by Tukey’s multiple comparisons test). *****P* < 0.0001. **(L)** ETV4 N-terminal deletion on the expression of ORC1 and ORC6 protein using Western blot assay.

**
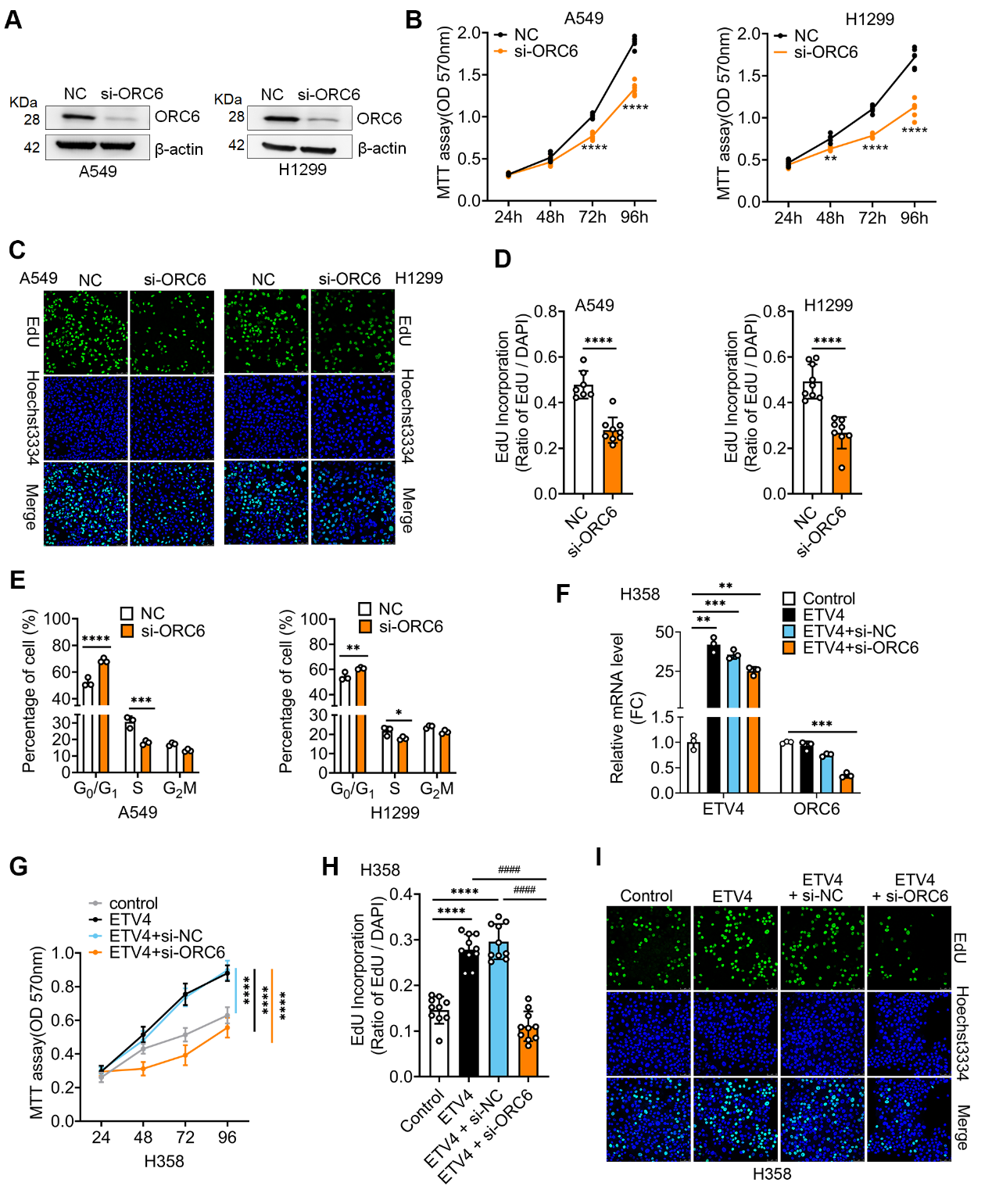
**

**Figure S6.** Depleting ORC6 reversed the effects of ETV4 on cell proliferation and EdU incorporation in NSCLC cells. **(A)** Immunoblots showing ORC6 protein levels in NC and siRNA mediated ORC6 knockdown cells. **(B)** Cell viability analysis of A549 and H1299 cells in the indicated periods (0 – 96 hrs) after transfection with NC or si-ORC6 (mean ± SD, n = 6; multiple *t*-test). ***P* < 0.01; *****P* < 0.0001. **(C, D)** EdU-incorporation assay of A549 and H1299 cells transfected with ORC6 or NC siRNA (mean ± SD, n = 3; two-tailed unpaired *t*-test). *****P* < 0.0001. (**E)** The cell cycle distribution of cells transfected with ORC6 or NC siRNA at a final concentration of 50 nM for 48 hrs by FCM assay (mean ± SD; n = 3; two-tailed unpaired *t*-test). **P* < 0.05; ***P* < 0.01; ****P* < 0.001; *****P* < 0.0001. **(F)** RT-qPCR showing ETV4 and ORC6 mRNA levels in H358-ETV4 cells transfected with ORC6 or NC siRNA. **(G-I)** MTT and EdU-incorporation analysis of H358-ETV4 cells transfected with ORC6 or NC siRNA (mean ± SD, multiple *t*-test). ***P* < 0.01; ****P* < 0.001; ^####^*P* < 0.0001.

**
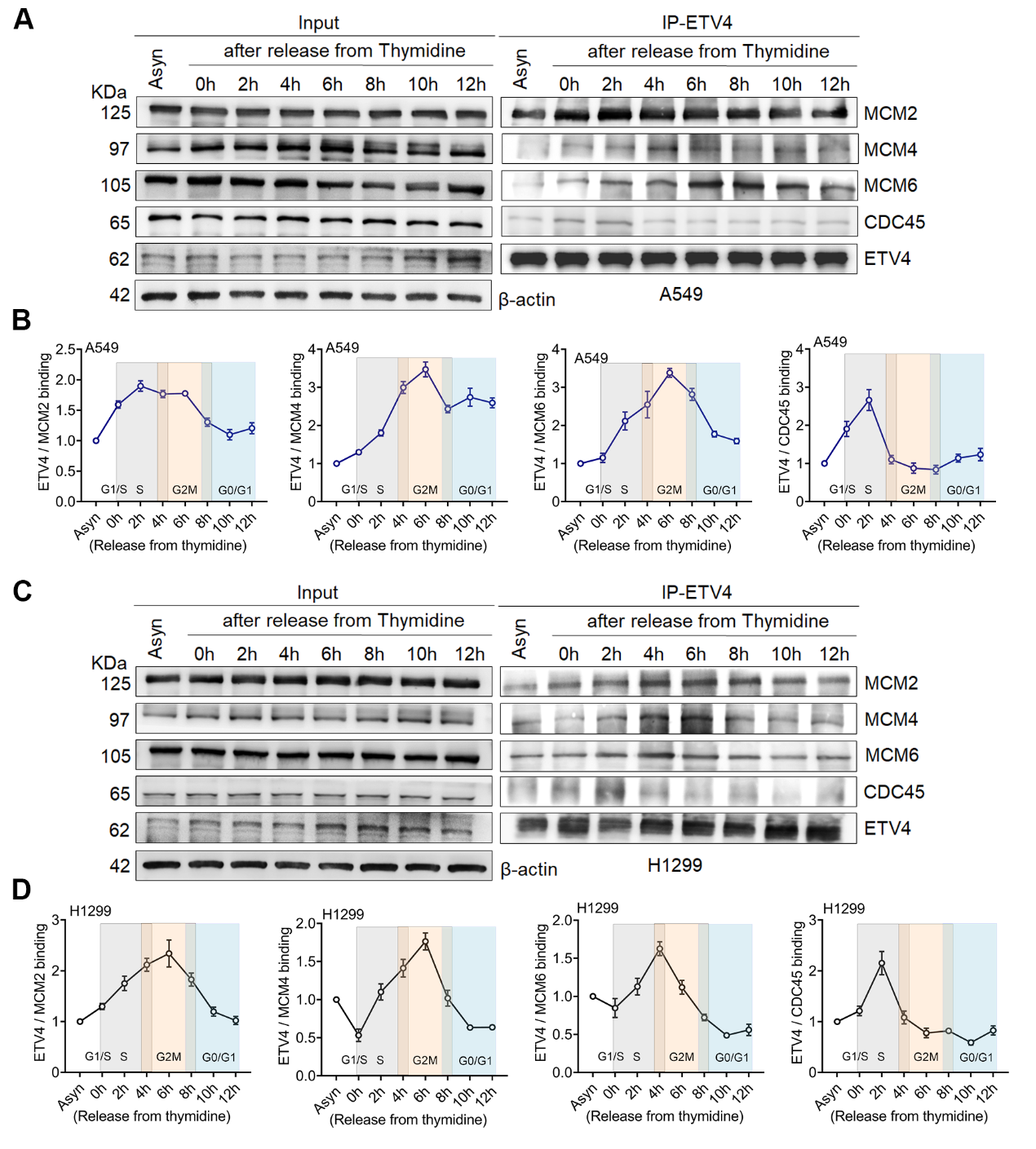
**

**Figure S7.** The associations of ETV4 with MCMs subunits in synchronized NSCLC cells. **(A)** IP of ETV4 and Immunoblots against MCM2, MCM4, MCM6, and CDC45 from thymidine block and released synchronized A549 cells. **(B)** Quantification of ETV4-protein binding in (A). **(C)** IP of ETV4 and Immunoblots against MCM2, MCM4, MCM6, and CDC45 from thymidine block and released synchronized H1299 cells. **(D)** Quantification of ETV4-protein binding in (C).


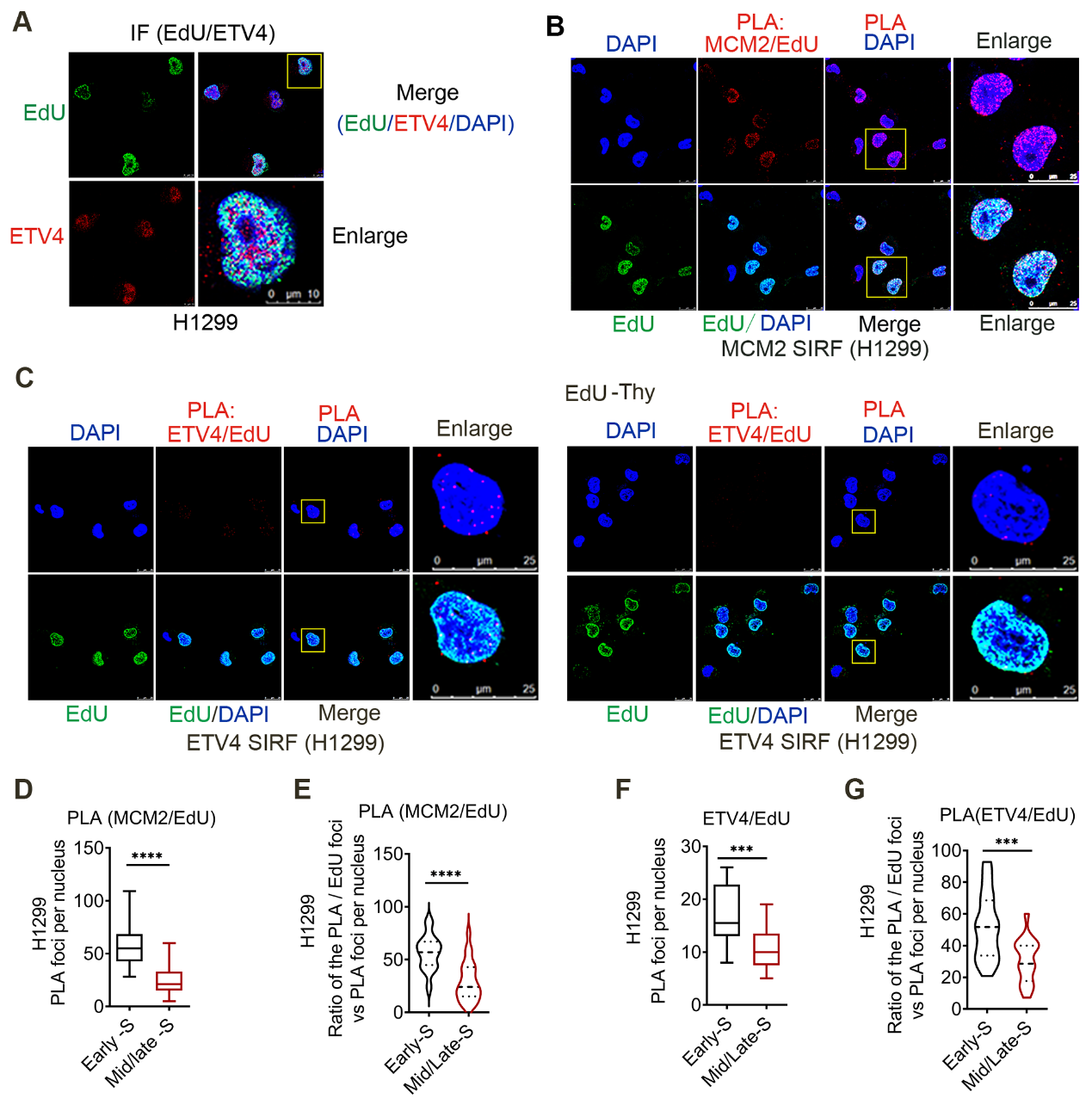


**Figure S8.** MCM2 and ETV4 are in proximity to newly replicated DNA in H1299 cells. **(A)** After a 15 min EdU pulse and covalent linkage to a biotin-azide using click chemistry, endogenous ETV4 co-localizes with sites of replication, marked by EdU incorporation, was detected in H1299 cells by EdU-labeling combined with immunostaining method. **(B)** After EdU pulse and click chemistry, the interaction of MCM2 with newly replicated DNA was detected in H1299 cells by SIRF assay. PLA of MCM2/EdU (Red) was performed using anti-biotin and anti-MCM2 antibodies. FITC-conjugated anti-biotin IF (Green) indicates cells undergoing DNA synthesis. DNA was counterstained with DAPI (Blue). **(C)** ETV4/EdU SIRF assay detected in H1299 cells labeled with 125 µM EdU for 15 min with or without 100 µM thymidine chases for 60 min. **(D, E)** Average MCM2 SIRF (PLA foci) signals or the ratio of the PLA/EdU foci (yellow) to the PLA foci per nucleus in early and later S-phase H1299 cells. **(F, G)** Average ETV4 SIRF (PLA foci) signals or the ratio of the PLA/EdU foci (yellow) to the PLA foci per nucleus in early and later S-phase H1299 cells. *P*-values for PLA signals in (D-G) were based on the two-tailed Mann-Whitney test. For all statistical tests, ****P* < 0.001; *****P* < 0.0001.


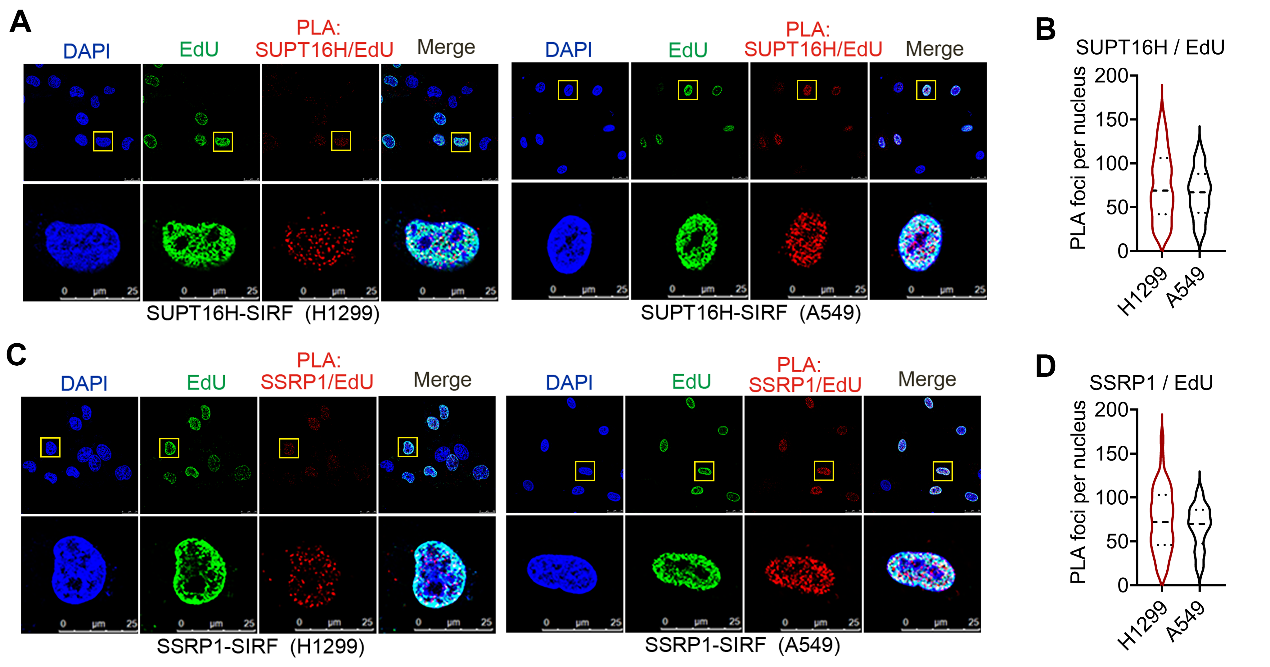


**Figure S9.** SUPT16H and SSRP1 are in proximity to newly replicated DNA in NSCLC cells. **(A, B)** SUPT16H/EdU SIRF assay was detected in H1299 and A549 cells labeled with 125 µM EdU for 15 min. PLA signal (Red) was performed using anti-biotin and anti-SUPT16H antibodies. FITC-conjugated anti-biotin IF (Green) indicates cells undergoing DNA synthesis. DNA was counterstained with DAPI (Blue). Violin diagram showing the average SUPT16H SIRF (PLA foci) signals per nucleus in H1299 and A549 cells. **(C, D)** SSRP1/EdU SIRF assay was detected in cells as in (A) using anti-biotin and anti-SSRP1 antibodies.

**
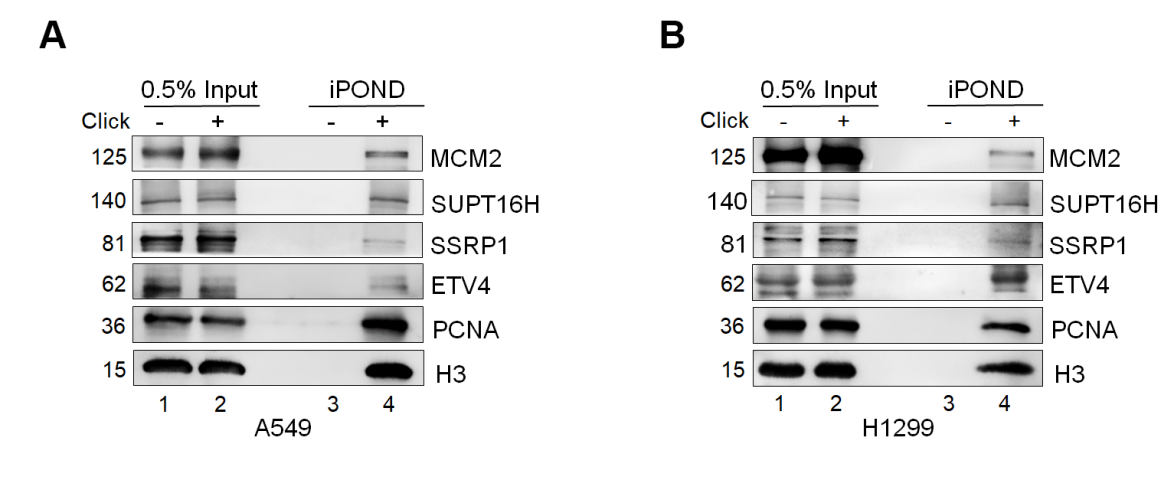
Figure S10.** MCM2, FACT, and ETV4 interact with newly replicated DNA in A549 **(A)** and H1299 **(B)** cells using iPOND analysis. 1 × 10^8^ cells were labeled with 10 μM EdU for 60 min. The protein-DNA complexes were cross-linked, nascent DNA was conjugated to biotin using click chemistry, and protein-DNA complexes were purified using Streptavidin agarose. The eluted proteins were analyzed using Western blot. A sample that did not include biotin-azide was used as a negative control (lane #3). The replisome component PCNA was used as positive control within each experiment to ensure the procedure worked as expected.


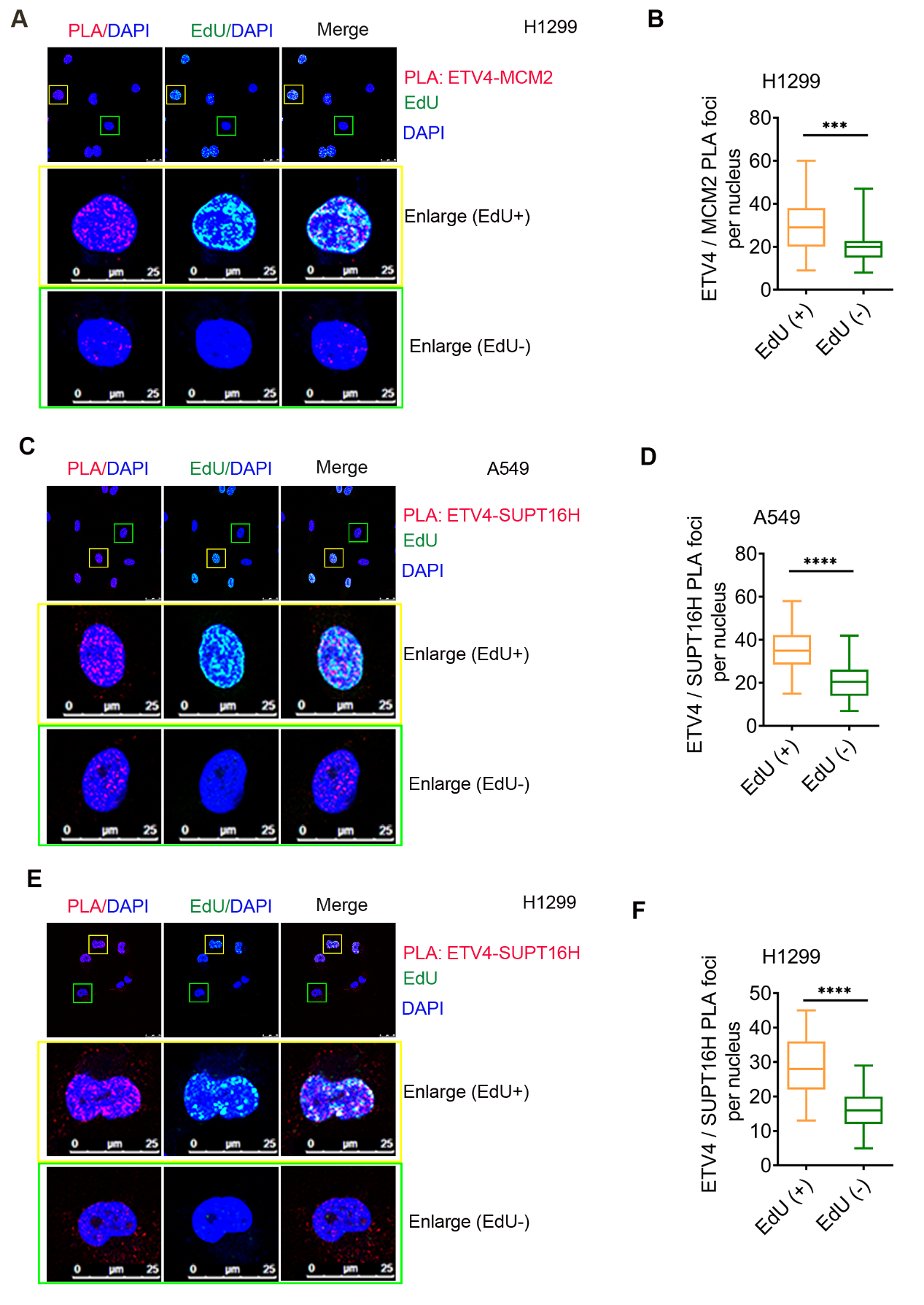


**Figure S11.** ETV4-MCM2 or ETV4-SUPT16H interaction occurs on nascent DNA during the S phase in NSCLC cells. **(A, B)** PLA between ETV4-MCM2 was performed using the Duolink® Proximity Ligation Assay, co-stained of EdU with a Cell-Light EdU Apollo 488 In Vitro Imaging Kit to label S-phase cells, and analyzed the proportion of PLA signal in EdU positive vs negative H1299 cells. Average PLA foci per nucleus in each group were analyzed using the two-tailed Mann-Whitney test. ****P* < 0.001. **(C-F)** PLA between ETV4-SUPT16H was performed, co-stained of EdU, and the proportion of PLA signal in EdU positive vs. negative A549 and H1299 cells were analyzed. Average PLA foci per nucleus in each group were analyzed using the two-tailed Mann-Whitney test. *****P* < 0.0001.

**
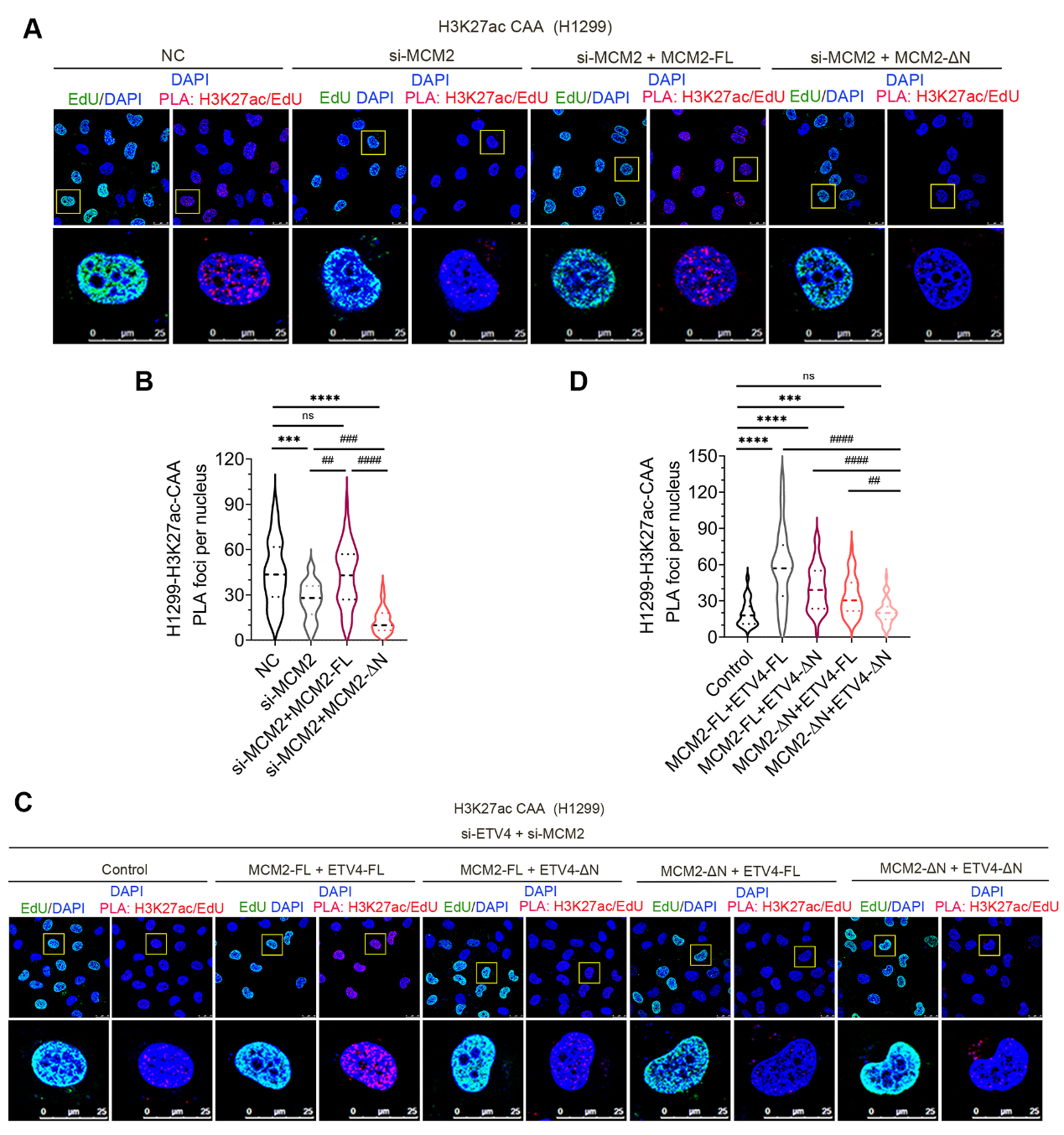
**

**Figure S12.** The effects of MCM2 and/or ETV4 N-terminal deletion on the histone acetylation level at replicating DNA in H1299 cells using Chromatin Assembly Assay (CAA). **(A)** H1299 cells were transfected with NC, si-MCM2, si-MCM2 + MCM2-FL, or si-MCM2 + MCM2-ΔN 48h after transfection, the cells were pulsed with 125 μM EdU for 15 min. CAA was performed for H3K27ac/EdU. FITC-conjugated anti-biotin IF (Green) indicates cells undergoing DNA synthesis. DNA was counterstained with DAPI (Blue). **(B)** Violin diagram showing the average PLA foci per nucleus in each group using the Kruskal-Wallis test with Dunnett’s multiple comparisons. ****P* < 0.001; *****P* < 0.0001; ^##^*P* < 0.01, ^###^*P* < 0.001. ^####^*P* < 0.0001. n.s., not significant. **(C)** H1299 cells were transfected with si-ETV4 and si-MCM2 for 24h, then MCM2 FL+ ETV4 FL, MCM2 FL + ETV4 ΔN, MCM2 ΔN + ETV4 FL, or MCM2 ΔN + ETV4 ΔN plasmids were transfected, respectively. 48h after transfection, cells were pulsed with 125 μM EdU for 15 min. CAA was performed for H3K27ac/EdU. **(D)** Violin diagram showing the average PLA foci per nucleus in each group using the Kruskal-Wallis test with Dunnett’s multiple comparisons. n.s., not significant; ****P* < 0.001; *****P* < 0.0001; ^##^*P* < 0.01, ^####^*P* < 0.0001.

**
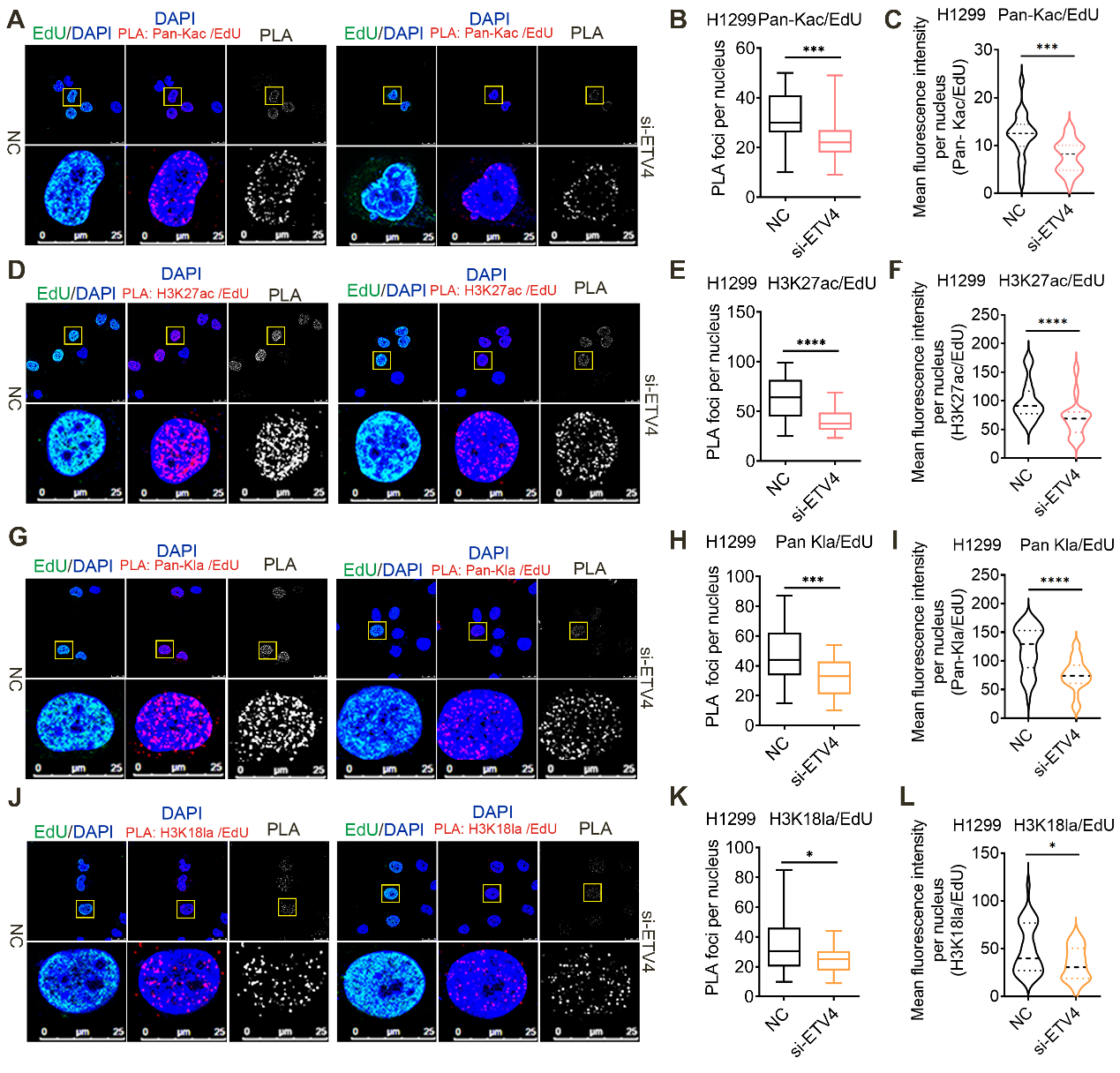
**

**Figure S13.** The effects of ETV4 on the histone acetylation and lacylation level near sites of DNA replication in H1299 cells. **(A)** H1299 cells were pulsed with 125 μM EdU for 15 min and CAA was performed for pan-Kac/EdU. FITC-conjugated anti-biotin IF (Green) indicates cells undergoing DNA synthesis. DNA was counterstained with DAPI (Blue). **(B, C)** Average PLA foci per nucleus (B) and the intensity of Immunofluorescence per nucleus (C) were determined. **(D-F)** CAA was performed for H3K27ac/EdU in H1299 cells as in A-C. **(G-I)** CAA was performed for Pan-kla/EdU in H1299 cells as in A-C. **(J-L)** CAA was performed for H3K18la/EdU as in A-C. All CAA data were analyzed using the two-tailed Mann-Whitney test. For all statistical tests, **P* < 0.05; ****P* < 0.001; *****P* < 0.0001.

**
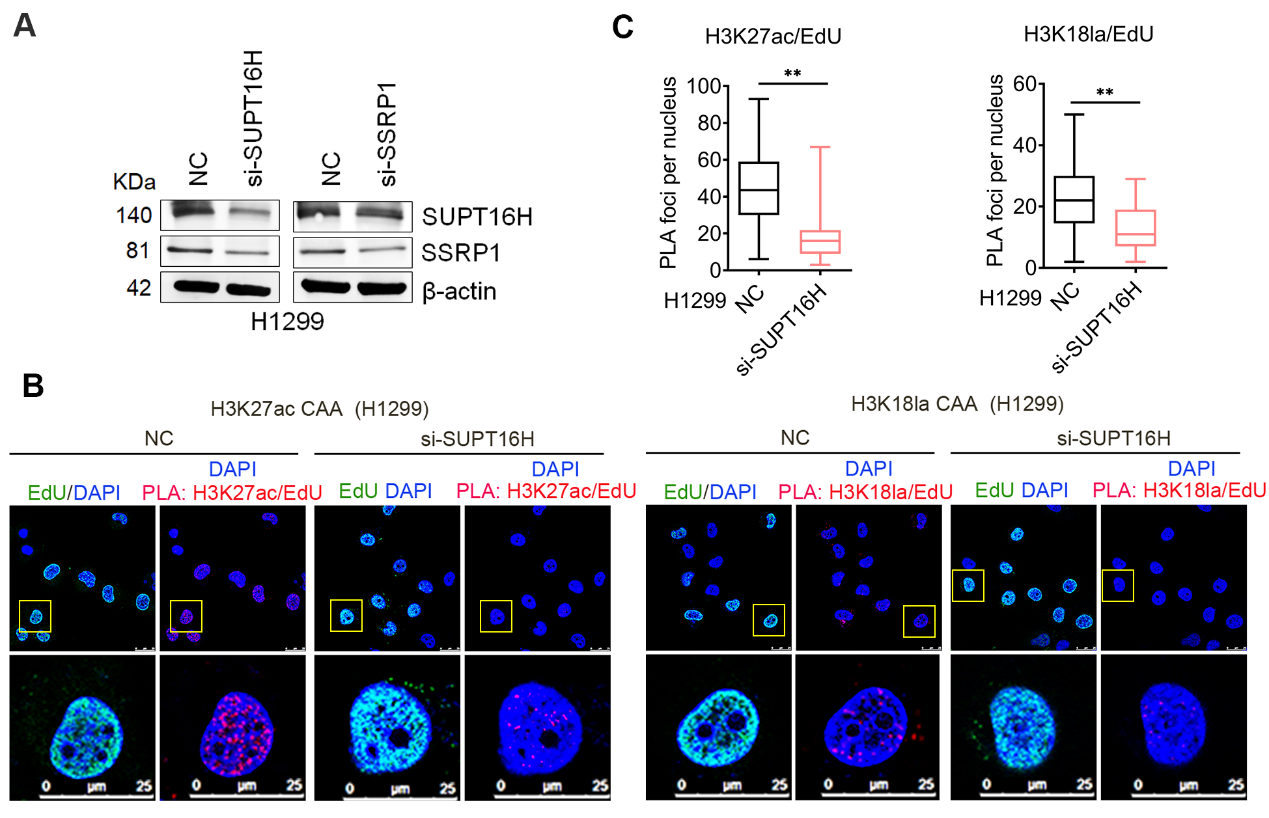
**

**Figure S14.** SUPT16H knockdown on the H3K27ac and H3K18la level at replicating DNA in H1299 cells using CAA. **(A)** siRNA-mediated SUPT16H or SSRP1 knockdown on the expression of both proteins in H1299 cells using Western blot assay. **(B)** 48h after transfection of NC or si-SUPT16H, H1299 cells were pulsed with 125 μM EdU for 15min. CAA was performed for H3K27ac/EdU and H3K18la/EdU. FITC-conjugated anti-biotin IF (Green) indicates cells undergoing DNA synthesis. DNA was counterstained with DAPI (Blue). **(C)** Average PLA foci per nucleus in each group of (B) was analyzed using the two-tailed Mann-Whitney test. ***P* < 0.01.

**
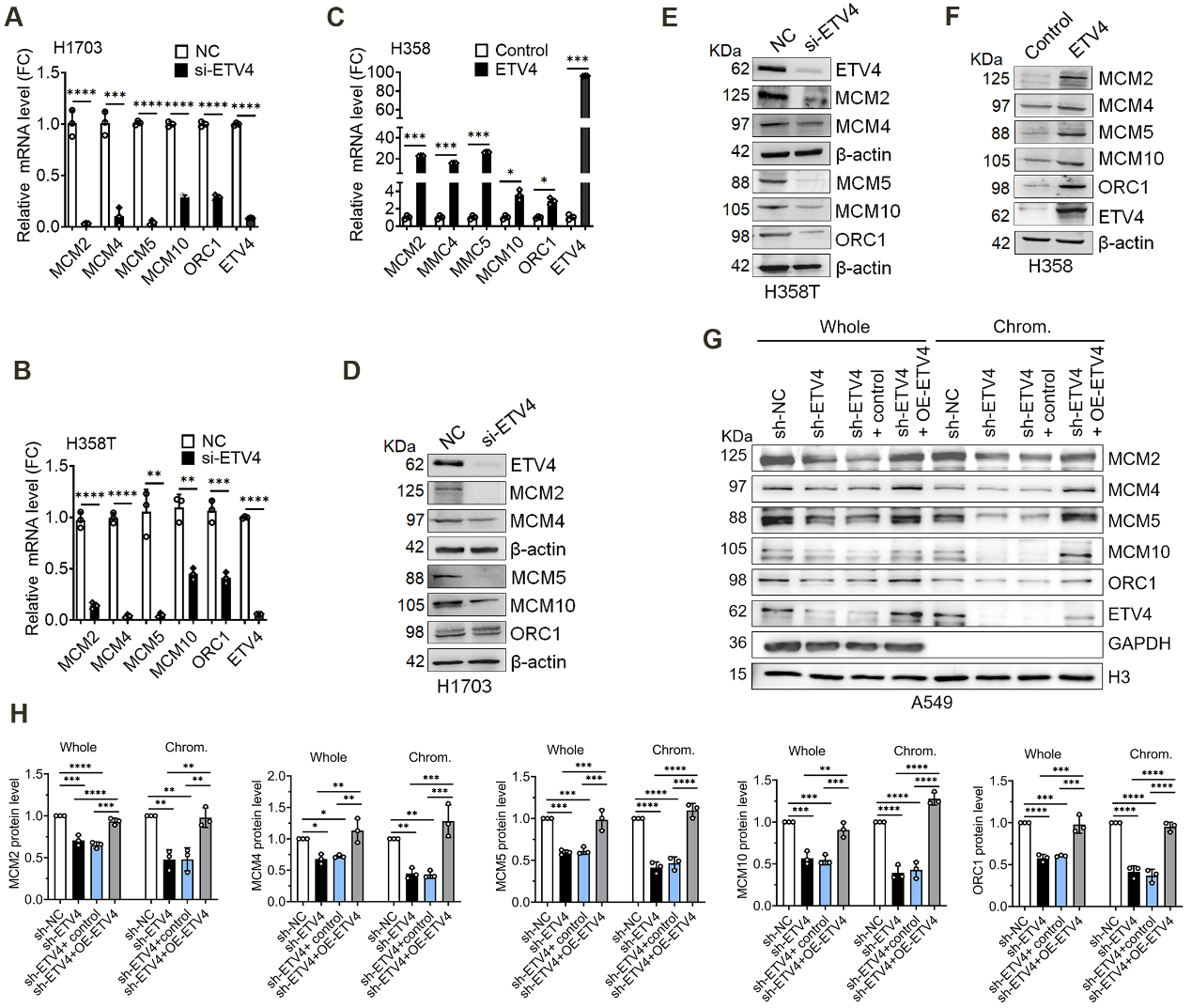
**

**Figure S15.** The effects of ETV4 on the expressions of Pre-RC factors MCM2, -4, -5, -10, and ORC1 in NSCLC cells. **(A-C)** RT-qPCR analysis of MCM2, -4, -5, -10, and ORC1 mRNA expression in H1703 and H358T cells transfected with ETV4 or NC siRNA, or H358 cells with ETV4 overexpression. Transcript levels were normalized to ACTB gene expression (mean ± SD, n = 3; two-tailed unpaired *t*-test). **P* < 0.05; ***P* < 0.01; ****P* < 0.001; *****P* < 0.0001. **(D-F)** Immunoblots showing MCM2, -4, -5, -10, and ORC1 protein levels in NC and ETV4-knockdown H1703 and H358T cells, or control and ETV4-overexpression H358 cells. **(G)** Immunoblots showing the levels of MCM2, -4, -5, -10, and ORC1 protein in control, sh-ETV4, and sh-ETV4 + OE-ETV4 A549 cells from whole-cell proteins or chromatin-bound proteins. **(H)** Quantification of G (n = 3) presented as mean ± SD. One-way ANOVA, **P* < 0.05; ***P* < 0.01; ****P* < 0.001; *****P* < 0.0001.

**
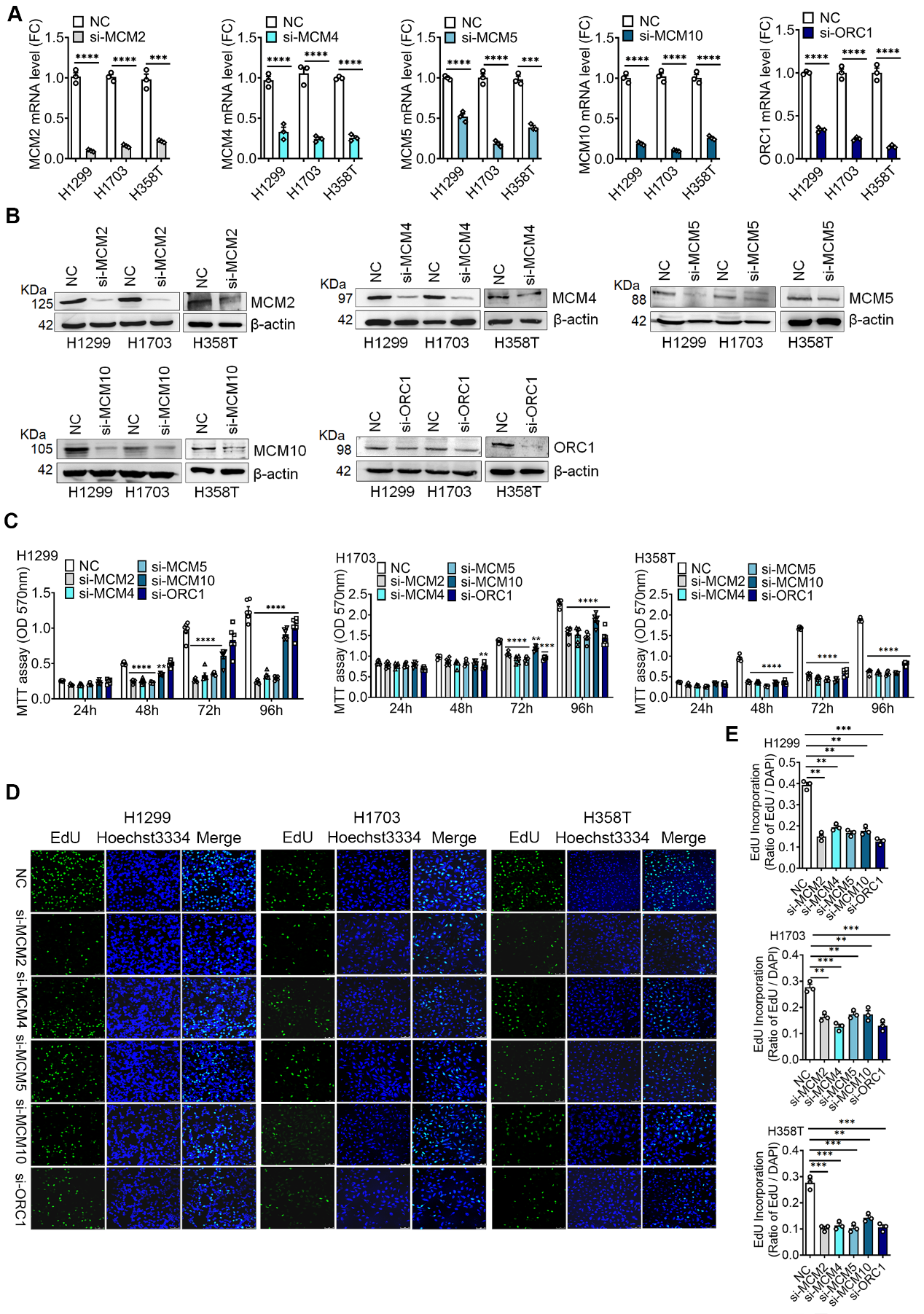
**

**Figure S16.** Knockdown of MCMs and ORC1 expression inhibits NSCLC cell proliferation. **(A)** The relative MCM2, -4, -5, -10, and ORC1 mRNA expressions in H1299, H1703, and H358T cells transfected with MCM2, -4, -5, -10, ORC1, or NC siRNA at a final concentration of 50 nM for 48 hrs (mean ± SD, n = 3; two-tailed unpaired *t*-test). ****P* < 0.001; *****P* < 0.0001. **(B)** Immunoblots showing MCM2, -4, -5, -10, and ORC1 protein levels in NC and MCM2, -4, -5, -10, ORC1-knockdown cells. **(C)** Cell viability analysis of H1299, H1703, and H358T cells in the indicated periods (0 – 96 hrs) after transfection with NC or MCM2, -4, -5, -10, or ORC1 siRNA at a final concentration of 50 nM. (mean ± SD, n = 6; Two-way ANOVA with Bonferroni’s multiple comparisons test). ***P* < 0.01; ****P* < 0.001; *****P* < 0.0001. **(D, E)** EdU-incorporation assay of H1299, H1703 and H358T cells transfected with MCM2, -4, -5, -10, and ORC1 siRNA or NC siRNA (mean ± SD, n = 3; two-tailed unpaired *t*-test). ***P* < 0.01; ****P* < 0.001.

**
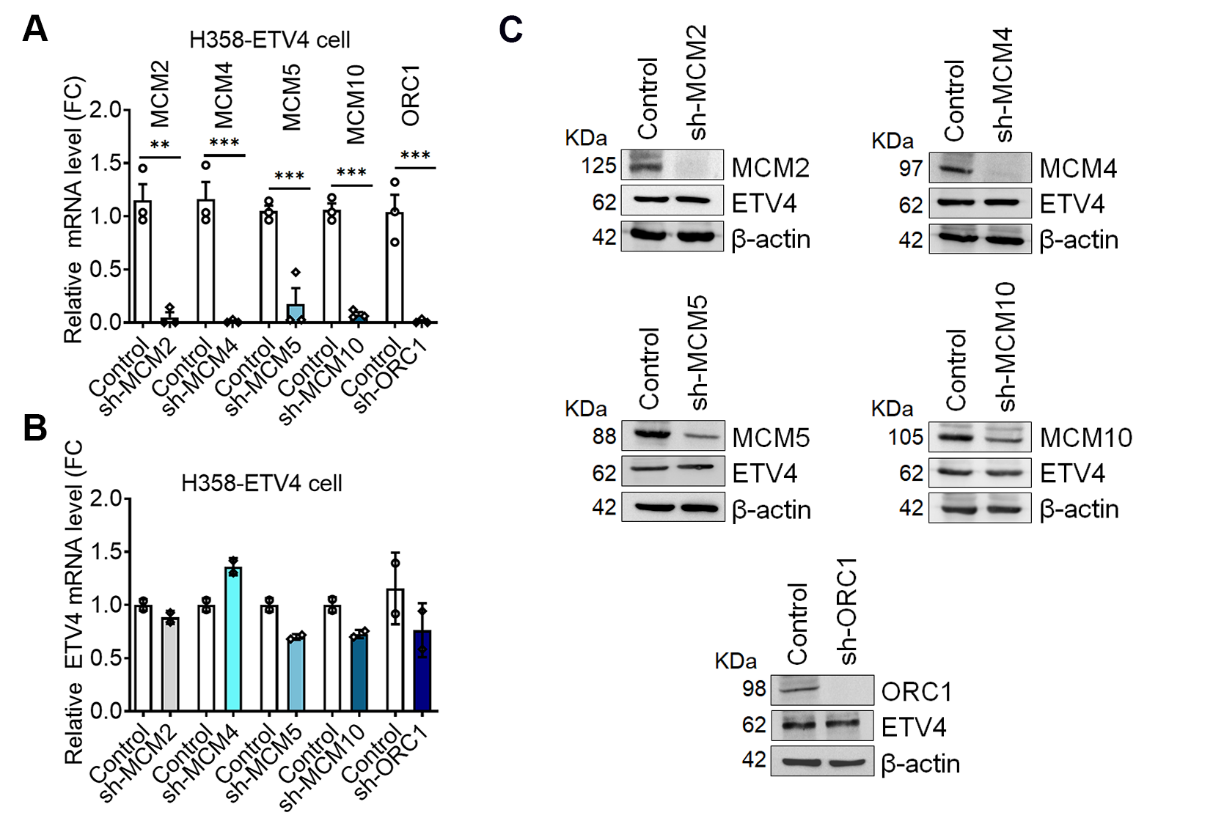
**

**Figure S17.** The efficiency of H358-ETV4 cells stably expressing shRNA targeting MCM2, -4, -5, -10, ORC1, or negative control plasmid. **(A, B)** H358-ETV4 cells stably expressing shRNA targeting MCM2, -4, -5, -10, ORC1, or negative control were generated after double selection using neomycin and puromycin. The relative mRNA expressions of MCM2, -4, -5, -10, ORC1, and ETV4 were determined by RT-qPCR. mean ± SD, n = 3; two-tailed unpaired *t*-test. ***P* < 0.01; ****P* < 0.001. **(C)** Immunoblots showing MCM2, -4, -5, -10, and ORC1 protein levels in H358-ETV4 cells stably expressing shRNA targeting MCM2, -4, -5, -10, ORC1, or negative control cells.

**
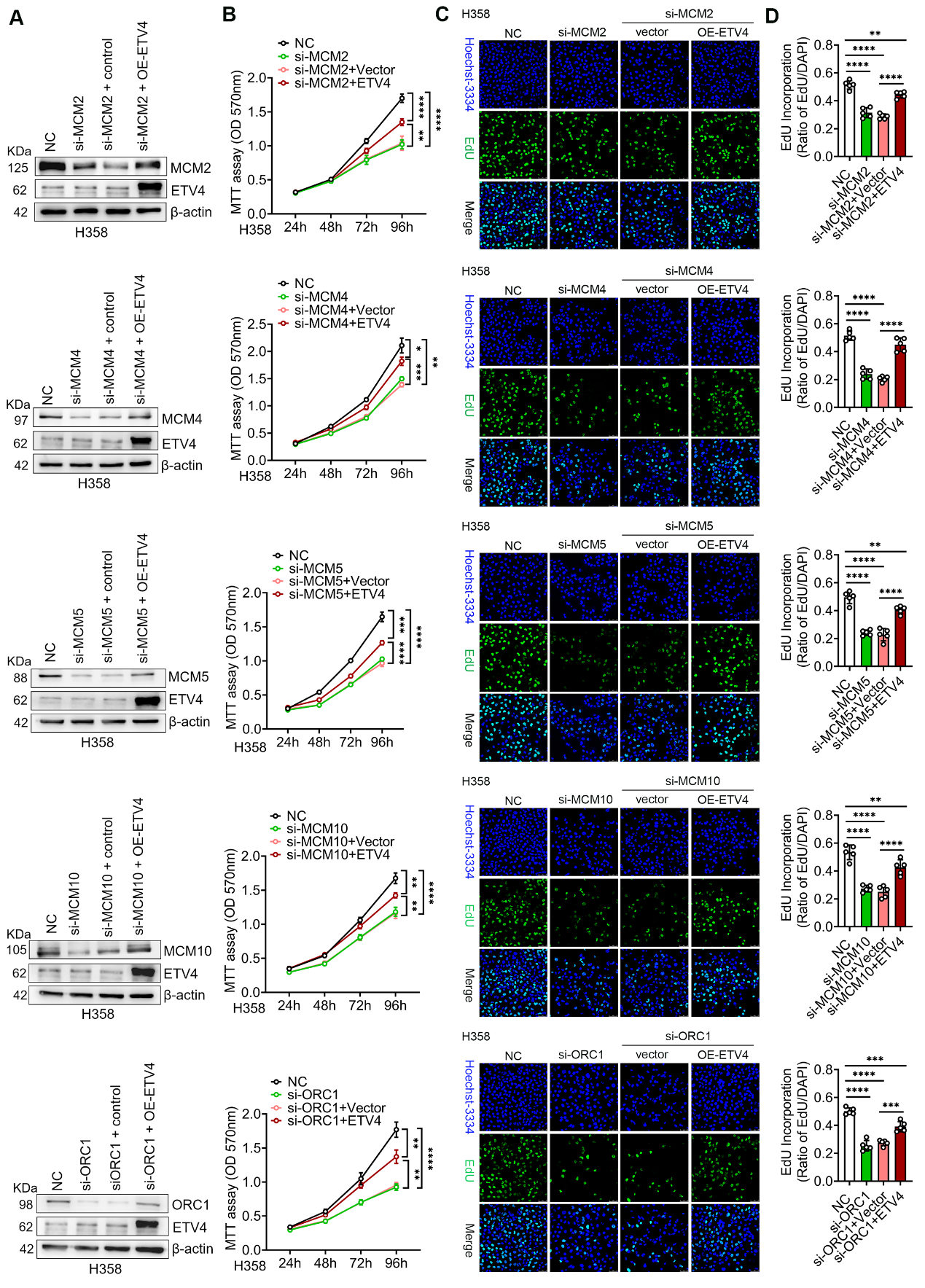
**

**Figure S18.** ETV4 overexpression on cell proliferation and EdU incorporation in MCM2, -4, -5, -10, or ORC1 deletion H358 cells. **(A)** Immunoblots showing the effects of ETV4 overexpression on the expression of MCM2, -4, -5, -10, and ORC1 protein in NC and MCM2, -4, -5, -10, ORC1-knockdown cells, respectively. **(B)** ETV4 overexpression on cell viability of H358 MCMs/ORC1 knockdown cells in the indicated periods using MTT assay (mean ± SD, n = 5; Two-way ANOVA with Bonferroni’s multiple comparisons test). **P* < 0.05; ***P* < 0.01; ****P* < 0.001; *****P* < 0.0001. **(C, D)** EdU-incorporation analysis of ETV4 overexpression in H358 MCMs/ORC1 knockdown cells (mean ± SD, multiple *t*-test). ***P* < 0.01; ****P* < 0.001; *****P* < 0.0001.


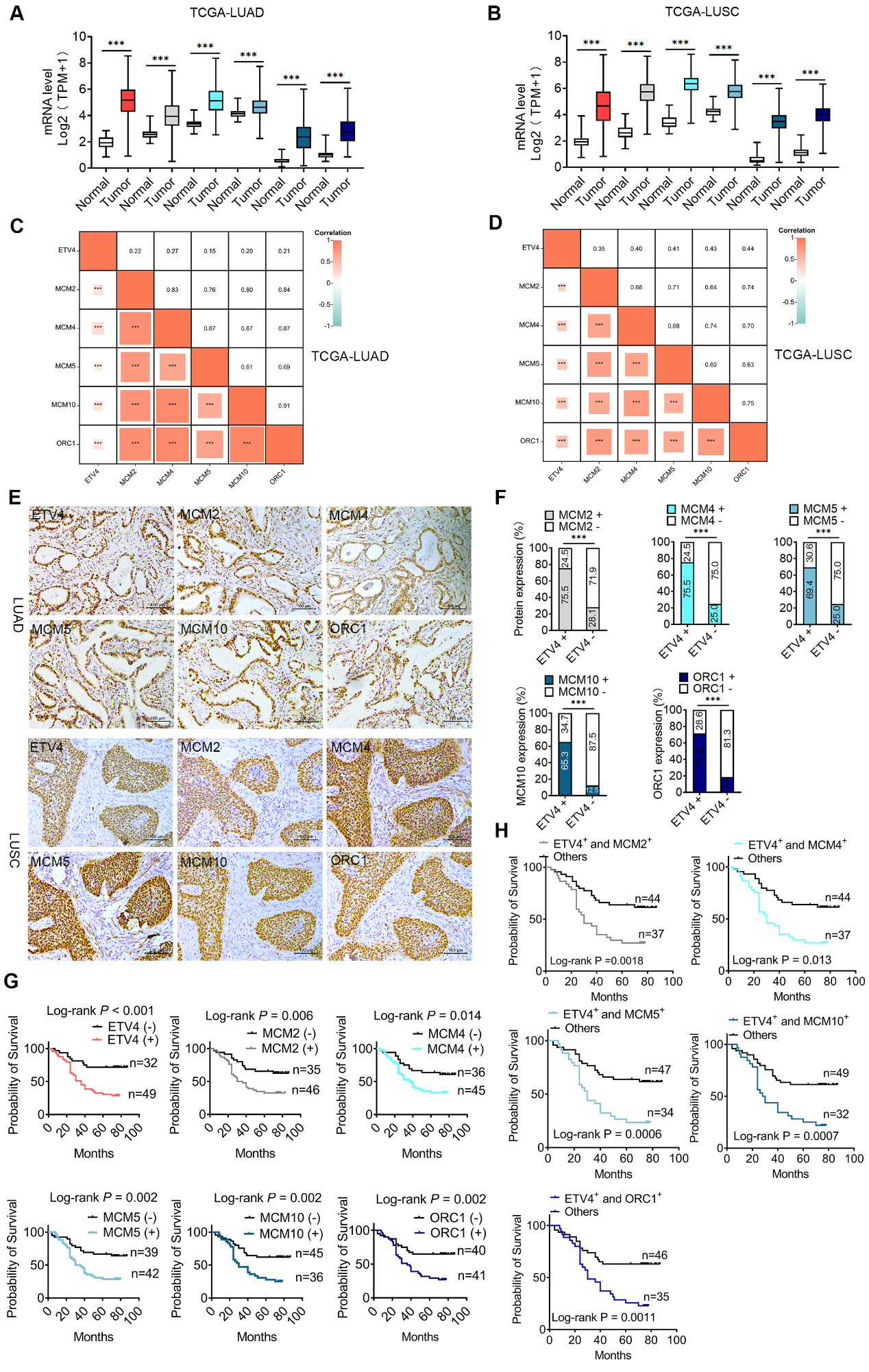


**Figure S19.** Upregulated MCMs and ORC1 expression correlated with ETV4 expression and poor survival. of NSCLC patients. **(A, B)** ETV4, MCM2/4/5/10, and ORC1 mRNA levels were all upregulated in 515 lung adenocarcinoma (LUAD) and 503 squamous cell carcinomas (LUSC) from TCGA datasets. Wilcoxon rank sum test. ****P* < 0.001. **(C, D)** The significant positive co-expression patterns between ETV4 and MCM2/4/5/10, ORC1 mRNA in LUSC and LUAD from TCGA datasets by using Spearman’s correlation analysis. **(E)** Representative images of IHC staining for ETV4, MCM2, -4, -5, -10, and ORC1 protein in the tissues of LUAD or LUSC (Scale bar, 100 µm). **(F)** The distribution of MCMs or ORC1 in ETV4-positive (n = 49) or ETV4-negative (n = 32) NSCLCs from 81 patients based on Two-way ANOVA. ****P* < 0.001. **(G)** Kaplan-Meier analysis of overall survival of NSCLC patients based on the expression level of ETV4, MCM2, MCM4, MCM5, MCM10, or ORC1. Log-rank (Mantel-Cox) test was used to calculate *P* values for survival analysis. **(H)** Kaplan-Meier analysis of overall survival of NSCLC patients based on the expression level of ETV4/MCM2, ETV4/MCM4, ETV4/MCM5, ETV4/MCM10, or ETV4/ORC1. Log-rank (Mantel-Cox) test was used to calculate *P* values for survival analysis.

**
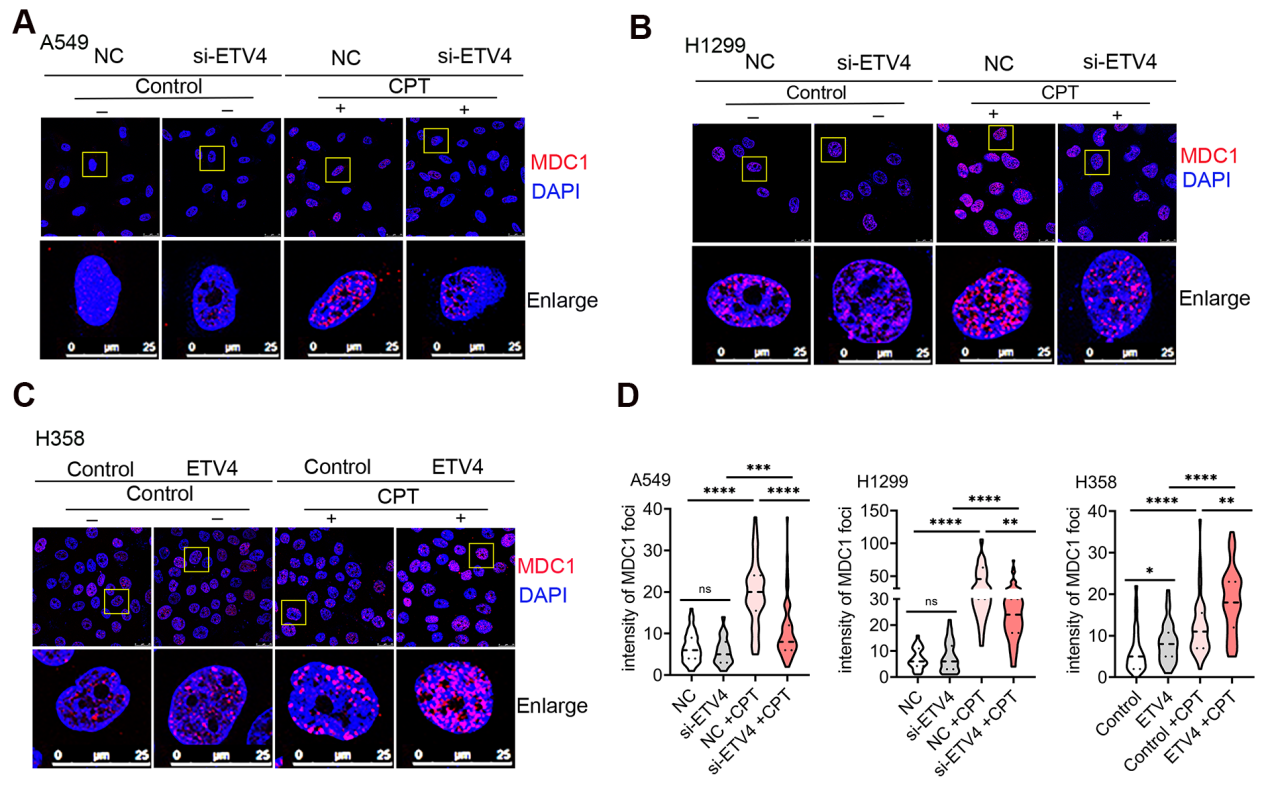
**

**Figure S20.** Immunofluorescence assay showing the accumulation of MDC1 foci in NSCLC cells after ETV4-knockdown or overexpression. **(A-C)** Immunofluorescence assay showing the accumulation of MDC1 foci in NC and ETV4-knockdown A549 and H1299 cells, or control and ETV4-overexpression H358 cells in the presence or absence of 25 μM CPT treatment for 30 min. **(D)** Violin plot quantification of MDC1 foci intensity per cell as shown in A-C. *P*-values for γ-H2AX signals were based on Kruskal-Wallis test with Dunnett’s multiple comparisons. ***P* < 0.01; ****P* < 0.001; *****P* < 0.0001.

**
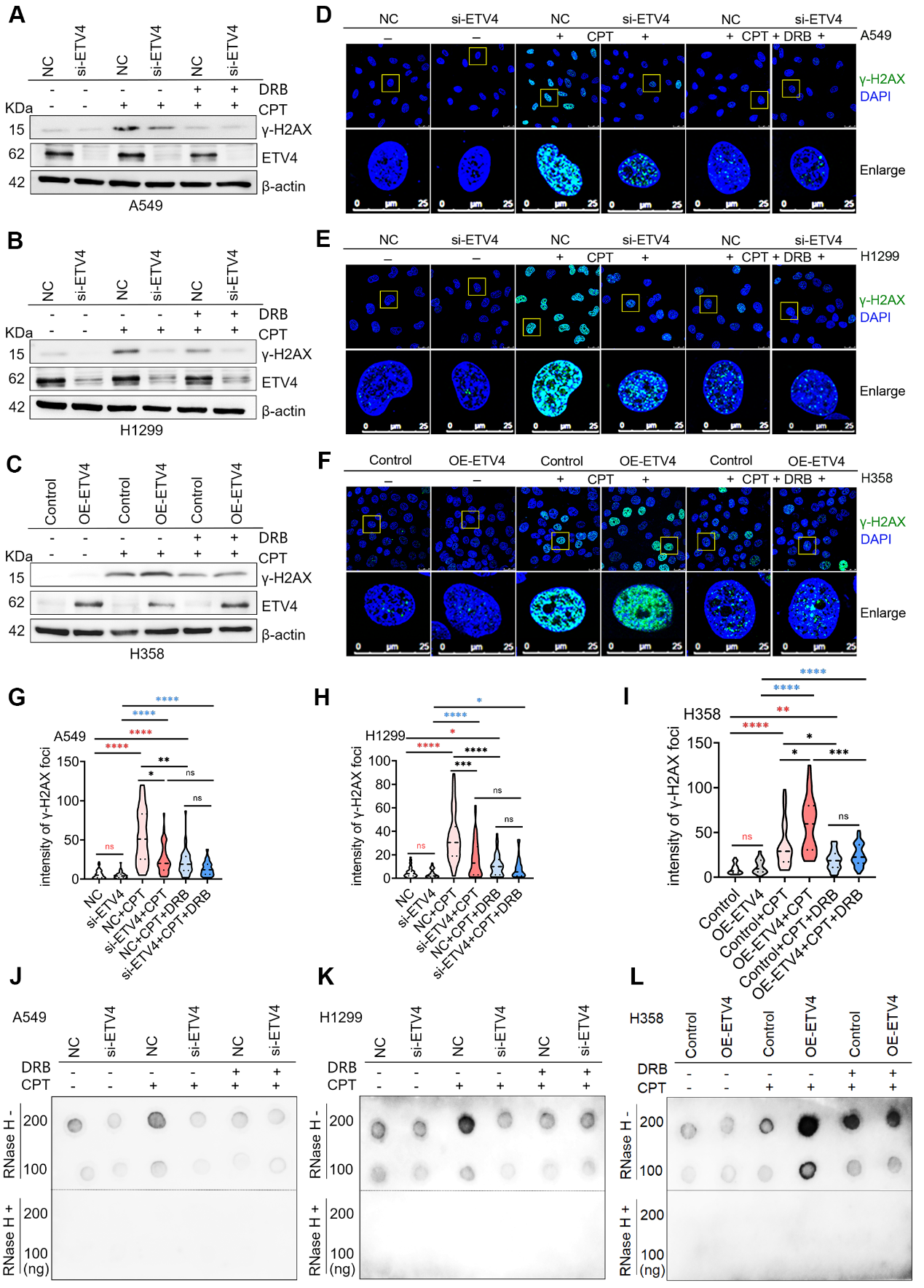
**

**Figure S21.** The effects of RNAPII-mediated transcription inhibitor DRB in combination with CPT treatment on γ-H2AX and R-loop formation in ETV4-knockdown or overexpression NSCLC cells. **(A-C)** Immunoblots showing γ-H2AX protein levels in NC and ETV4-knockdown A549 and H1299 cells, or control and ETV4-overexpression H358 cells after DRB (100 μM, 3h) and/or CPT (25 μM, 30 min) treatment. **(D-F)** Immunofluorescence assay showing the accumulation of γ-H2AX foci in NC and ETV4-knockdown A549 and H1299 cells, or control and ETV4-overexpression H358 cells after DRB and/or CPT treatment. **(G-I)** Violin plot quantification of γ-H2AX foci intensity per cell as shown in D-F. P-values for γ-H2AX signals were based on the Kruskal-Wallis test with Dunnett’s multiple comparisons. n.s., not significant; **P* < 0.05; ***P* < 0.01; ****P* < 0.001; *****P* < 0.0001. **(J-L)** Dot blot analysis showing the abundance of R-loop in ETV4-knockdown A549 and H1299 cells, or control and ETV4-overexpression H358 cells treatments with DRB and/or CPT using the S9.6 antibody. RNase H treatment was included as a negative control.

**
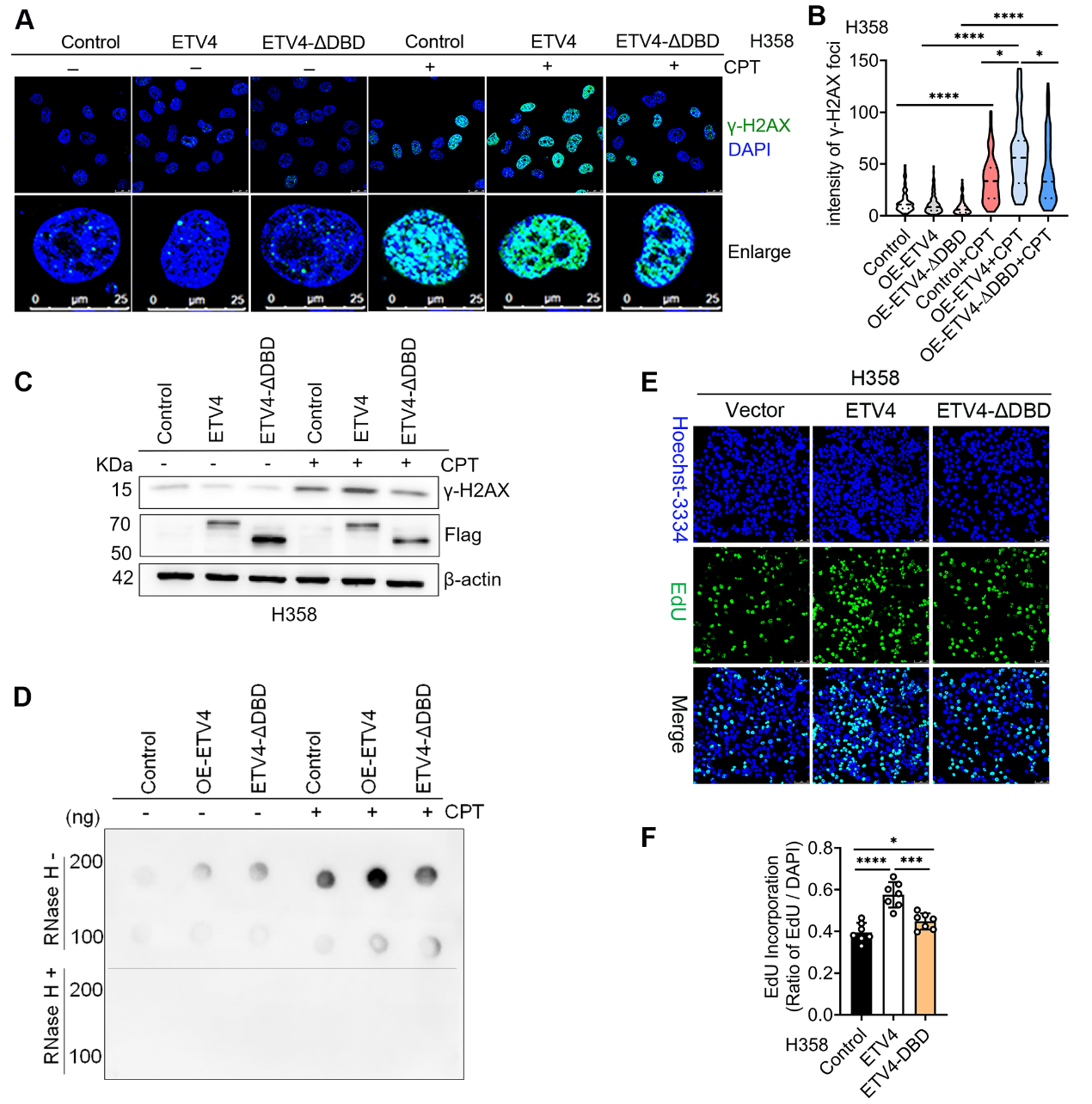
**

**Figure S22.** The effects of ETV4-DBD mutation on γ-H2AX and R-loop formation in H358 cells treated with or without CPT. **(A, B)** Immunofluorescence assay showing the accumulation of γ-H2AX foci in H358 cells transfected with ETV4 or ETV4-ΔDBD plasmids with or without CPT treatment. Violin plot quantification of γ-H2AX foci intensity per cell. Kruskal-Wallis test with Dunnett’s multiple comparisons. n.s., not significant; **P* < 0.05; *****P* < 0.0001. **(C)** Immunoblots showing γ-H2AX protein expression in H358 cells transfected with ETV4 or ETV4-ΔDBD plasmids with or without CPT treatment. **(D)** Dot blot analysis showing the abundance of R-loop in control, H358-ETV4 and H358-ΔDBD cells in the absence or presence of CPT using the S9.6 antibody. RNase H treatment was included as a negative control. **(E, F)** DNA replication was evaluated using EdU-incorporation assays in H358 cells transfected with ETV4 or ETV4-ΔDBD plasmids. Replicating cells label with EdU. (mean ± SD; n =7; two-tailed unpaired *t*-test). **P* < 0.05; ****P* < 0.001; *****P* < 0.0001.


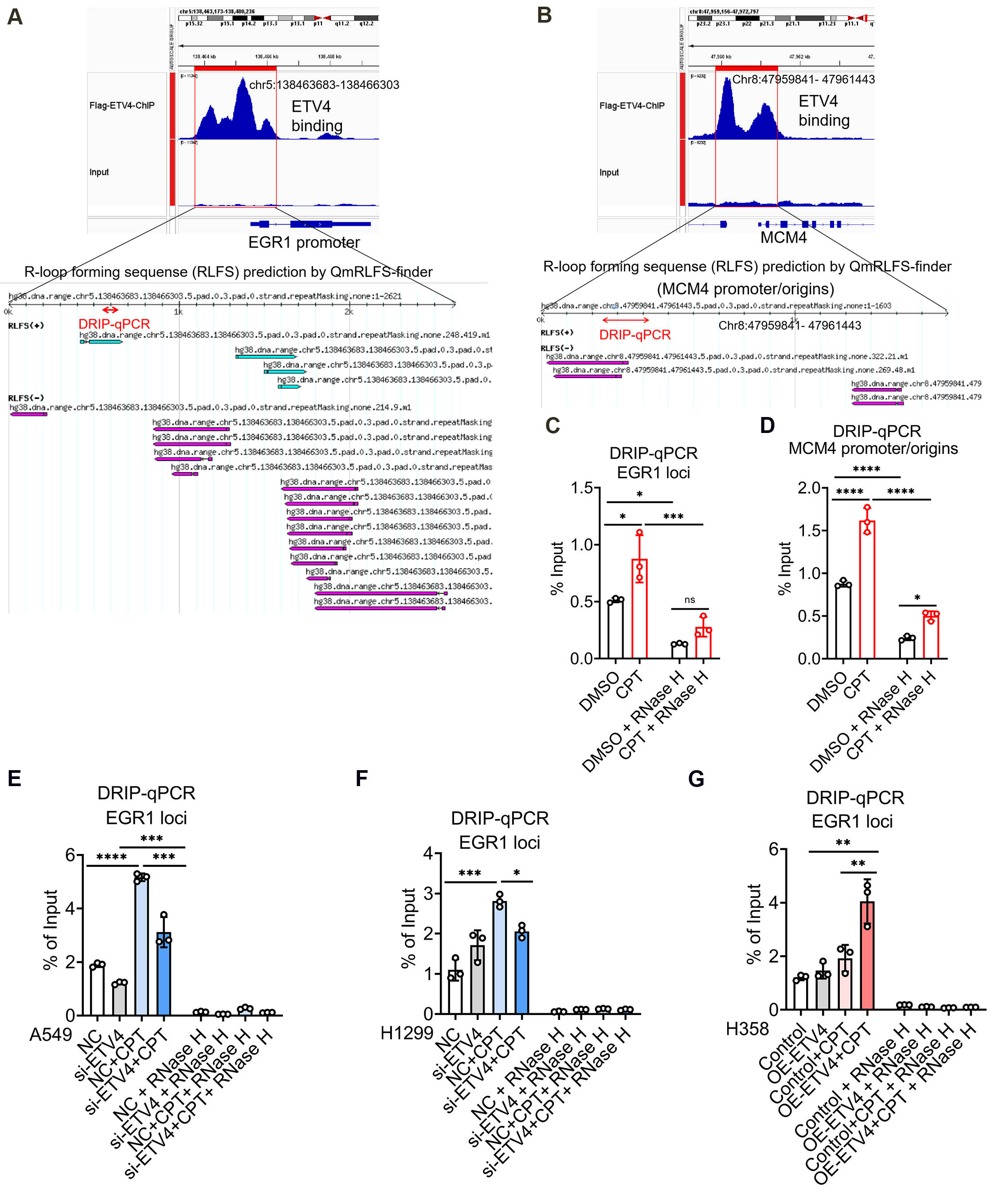


**Figure S23.** DRIP-qPCR assays showing the relationship between ETV4 expression and CPT-induced R-loop accumulation at precise genomic loci. **(A, B)** R-loop forming sequence (RLFS) prediction by QmRLFS-finder in ETV4 binding regions from our Flag-ETV4 ChIP-seq data at EGR1 and MCM4 Promoter/origins loci. **(C, D)** CPT treatment on R-loop accumulation at EGR1 and MCM4 loci in H1299 cells with or without RNase H treatment using DRIP-qPCR analysis. Ordinary one-way ANOVA with Tukey’s multiple comparisons test). **P* < 0.05; ****P* < 0.001; *****P* < 0.0001. **(E-G)** DRIP-qPCR assays mapping the R-loop accumulation at EGR1 loci in ETV4-knockdown A549 and H1299 cells, or H358-ETV4 cells treated with or without CPT using the S9.6 antibody. RNase H treatment was included as a negative control. Ordinary one-way ANOVA with Tukey’s multiple comparisons test. **P* < 0.05; ***P* < 0.01; ****P* < 0.001; *****P* < 0.0001.

**
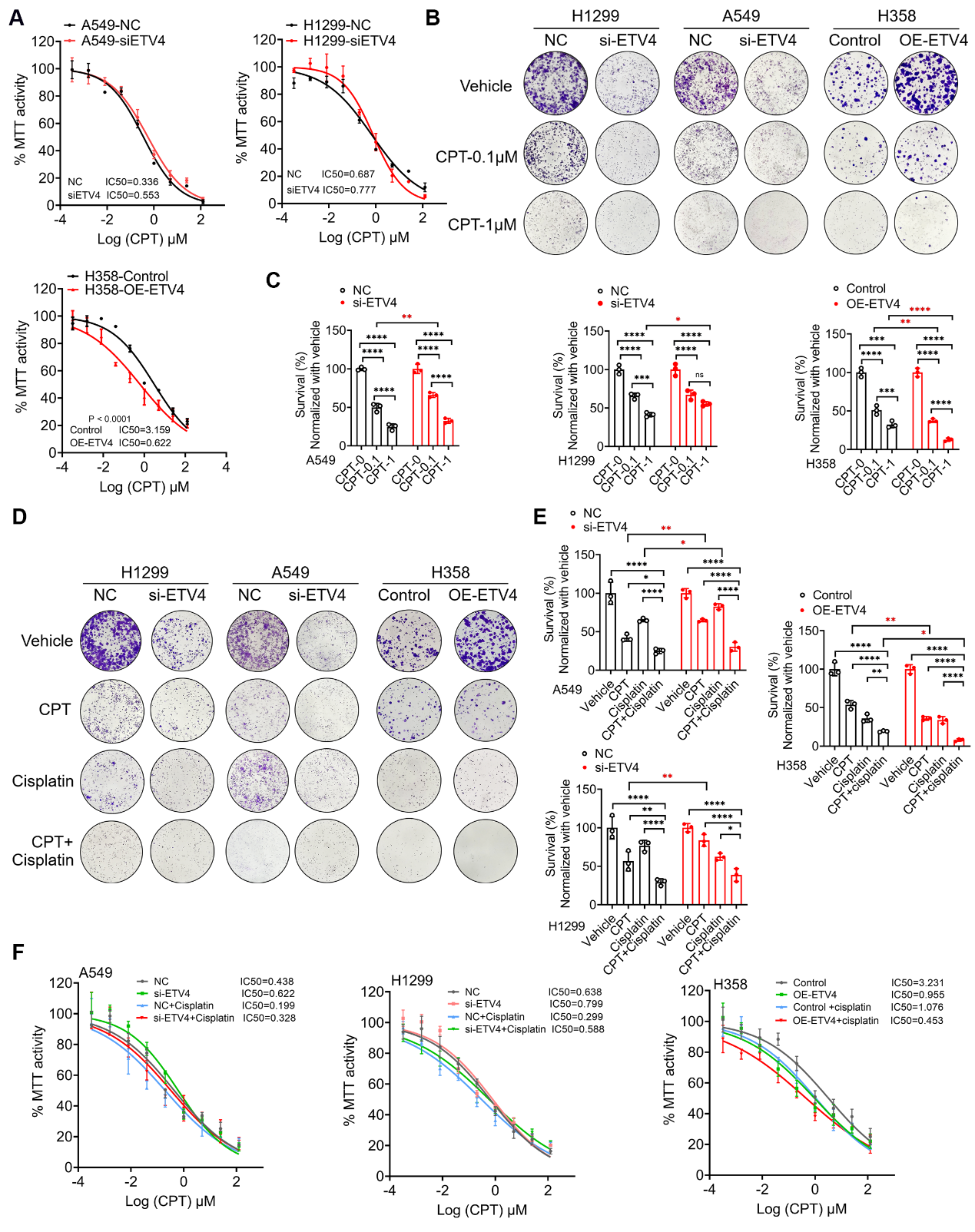
**

**Figure S24.** The effects of CPT and/or cisplatin on cell survival after ETV4 knockdown or overexpression. **(A)** MTT assay of A549 and H1299 ETV4-knockdown cells and H358-ETV4 cells in response to a concentration range of CPT at 1:4 dilution starting at 125 μmol/L. IC_50_ values are shown for each group. *P* values were calculated using the extra-sum-of-squares F test. n = 4 biological experiments. **(B)** Clonogenic assay after CPT treatment in ETV4 knockdown A549 and H1299 cells or H358 overexpression cells. **(C)** Quantification of B (n = 3) presented as mean ± SD. Two-way ANOVA with Sidak's multiple comparisons test. **P* < 0.05; ***P* < 0.01; ****P* < 0.001, *****P* < 0.0001. **(D)** Clonogenic assay after CPT (1 μM), cisplatin (10 μM), or CPT combines cisplatin treatment for 24h in ETV4 knockdown A549 and H1299 cells or H358 overexpression cells. **(E)** Quantification of D presented as mean ± SD. Two-way ANOVA with Sidak's multiple comparisons test. **P* < 0.05; ***P* < 0.01; *****P* < 0.0001. **(F)** Forty eight-hour MTT assay of ETV4 knockdown or overexpression cells in response to CPT and cisplatin combination. IC_50_ values to CPT are shown for each cell line. *P* values were calculated using the extra-sum-of-squares F test. n = 4 biological experiments. **P* < 0.05; ***P* < 0.01; ****P* < 0.001; *****P* < 0.0001.

**
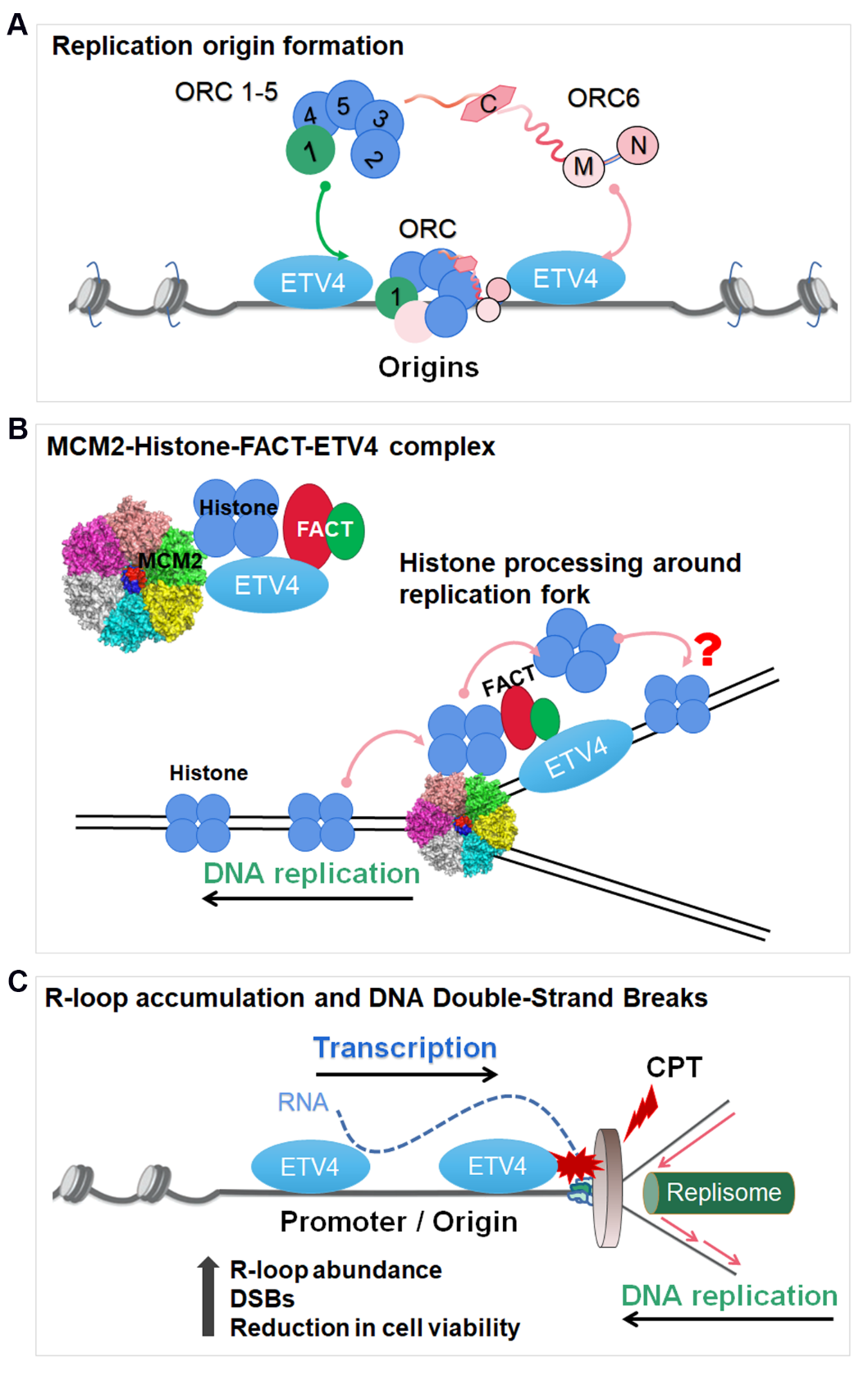
**

**Figure S25.** Schematic model illustrating the mechanisms by which ETV4-mediated DNA replication in NSCLC cells.

**Supplementary Table. 1** Relationships between ETV4, MCM2, MCM4 expression and clinicopathologic parameters in 81 NSCLCs

| **Characteristics** | **cases** | **ETV4** | ***P***  **value** | **MCM2** | ***P***  **value** | **MCM4** | ***P***  **value** |
| --- | --- | --- | --- | --- | --- | --- | --- |
|  |  | **+ -** |  | **+ -** |  | **+ -** |  |
| **All cases** | 81 | 49 32 |  | 46 35 |  | 45 36 |  |
| **Age (y)**  <=60  >60 | 37  44 | 21 16  28 16 | 0.528 | 20 17  26 18 | 0.649 | 20 17  25 19 | 0.803 |
| **Gender**  Male  Female | 62  19 | 41 21  8 11 | 0.061 | 35 27  11 8 | 0.912 | 37 25  8 11 | 0.177 |
| **Histological type**  SCC  AC | 37  44 | 21 16  28 16 | 0.528 | 23 14  23 21 | 0.371 | 20 17  25 19 | 0.803 |
| **Tumor size**  <=3cm  >3cm | 37  44 | 16 21  33 11 | 0.004 | 13 24  33 11 | <0.001 | 12 25  33 11 | <0.001 |
| **Lymphnode metastasis**  Negative  Positive | 43  38 | 14 29  35 3 | <0.001 | 14 29  32 6 | <0.001 | 14 29  31 7 | <0.001 |
| **Metastasis**  No  Yes | 69  12 | 37 32  12 0 | 0.007 | 34 35  12 0 | 0.001 | 33 36  12 0 | 0.001 |
| **Stage**  I  II  III  IV | 43  22  1  15 | 14 29  20 2  1 0  14 1 | <0.001 | 14 29  17 5  1 0  14 1 | <0.001 | 14 29  16 6  1 0  14 1 | <0.001 |

**Supplementary Table. 2** Relationships between MCM5, MCM10, ORC1 expression and clinicopathologic parameters in 81 NSCLCs

| **Characteristics** | **cases** | **MCM5** | | ***P***  **value** | **MCM10** | ***P***  **value** | **ORC1** | ***P***  **value** |
| --- | --- | --- | --- | --- | --- | --- | --- | --- |
|  |  | **+ -** | |  | **+ -** |  | **+ -** |  |
| **All cases** | 81 | 42 39 | |  | 36 45 |  | 41 40 |  |
| **Age (y)**  <=60  >60 | 37  44 | 21 16  21 23 | | 0.418 | 14 23  22 22 | 0.273 | 17 20  24 20 | 0.441 |
| **Gender**  Male  Female | 62  19 | 35 27  7 12 | | 0.134 | 29 33  7 12 | 0.446 | 34 28  7 12 | 0.170 |
| **Histological type**  SCC  AC | 37  44 | 18 19  24 20 | | 0.597 | 16 21  20 24 | 0.842 | 19 18  22 22 | 0.904 |
| **Tumor size**  <=3cm  >3cm | 37  44 | 13 24  29 15 | | 0.006 | 11 26  25 19 | 0.015 | 13 24  28 16 | 0.011 |
| **Lymphnode metastasis**  Negative  Positive | 43  38 | 12 31  30 8 | | <0.001 | 8 35  28 10 | <0.001 | 11 32  30 8 | <0.001 |
| **Metastasis**  No  Yes | 69  12 | 32 37  10 2 | | 0.018 | 25 44  11 1 | <0.001 | 29 40  12 0 | <0.001 |
| **Stage**  I  II  III  IV | 43  22  1  15 | 13 30  17 5  0 1  12 3 | <0.001 | | 8 35  14 8  1 0  13 2 | <0.001 | 11 32  15 7  1 0  14 1 | <0.001 |

**Supplementary** Table. 3 Primers used for siRNA, RT-qPCR, ChIP-PCR, ChIP-qPCR, and luciferase reporter construction in this study.

| siRNA sequences | | |
| --- | --- | --- |
| Gene | Forward (5’-3’) | Reverse (5’-3’) |
| ETV4 | CCAGACAAAUCGCCAUCAATT | UUGAUGGCGAUUUGUCUGGTT |
| MCM2 | GGAUUCCUUGGGAUUCUGGTT | CCAGAAUCCCAAGGAAUCCTT |
| MCM4 | GGCUCUCAUCGAGGCUUAUTT | AUAAGCCUCGAUGAGAGCCTT |
| MCM5 | GGAUCUGGCCAGCUUUGAUTT | AUCAAAGCUGGCCAGAUCCTT |
| MCM10 | CGGCGACGGUGAAUCUUAUTT | AUAAGAUUCACCGUCGCCGTT |
| ORC1 | CUGCACUACCAAACCUAUATT | UAUAGGUUUGGUAGUGCAGTT |
| ORC6 | UGUACAGCCUAAGUUAAUAAAUGTT | AACAUUUAUUAACUUAGGCUGUACAAU |
| SUPT16H | CGAGUGAAGAGACUGUACATT | UGUACAGUCUCUUCACUCGTT |
| SSRP1 | GACUUAAACUGCUUACAAATT | UUUGUAAGCAGUUUAAGUCTT |
| Negative control | UUCUCCGAACGUGUCACGUTT | ACGUGACACGUUCGGAGAATT |
| Primers for RT-qPCR | | |
| Gene | Forward (5’-3’) | Reverse (5’-3’) |
| ETV4 | GAAGGAGACATCAAGCAGGAA | AGCAAGGCCACCAGAAAT |
| MCM2 | AAGCCAGGAGAC GAGATAGA | CTAGGATGACAGTGGCAAAGA |
| MCM4 | GCA GACACCACACACAGTTA | CGAATAGGCACAGCTCGATAG |
| MCM5 | CAATGAGGAGAGGGATGTGATG | CACTCGGCAGTAGGCAATAA |
| MCM10 | GGTTCAGAGGAGGTGTGTTTAT | CGGCTCTCCATTCTTCTTCTT |
| ORC1 | AACTCCTACCCATCCTCTTACC | GAATCCAAGGAGGCACATGAA |
| ORC6 | CCCTTGGACAGGGCTTATTT | CAGGCCCAGTAAACACTCAA |
| ACTB | AGCGAGCATCCCCCAAAGTT | GGGCACGAAGGCTCATCATT |
| Primers for anti-ETV4 ChIP-PCR (gene promoter) | | |
| MCM2 | AGCAAAATGGCCCTGGTGTGACCGA | TGGCGACCAGAAGGGACACGGAGGG |
| MCM4 | CCGCAGCAGGGAGCAACGCACA | GCCAGGCCTTCCCGCAGGGGTC |
| MCM5 | CATCTTTGCGTCCGCCCCGTCCT | ACCGTTCTGAGCCCTCCAGCCCC |
| MCM10 | CCAGAGCGTCAGGAGTTGAGGT | ATCGGATTGGTTTACGTCATTG |
| ORC1 | AAAGCACCTAGAGGAATGGAGAAAG | CCGCCCACTCCGTACACCTAAGA |
| Primers for ChIP-(q)PCR (Origins) | | |
| MCM4-ori | CTTTGGCCCGAATCAACATG | CCTCGCTTGTTTTATCTGCCTCT |
| LAMB2-ori | CGCCGACTCGTAACTAACTG | GTCGTGTGGGAGTTGTAGTC |
| JUNB-locus | ATAAAGGCGTGTGGCTCAGG | ATAGCTTTCCTGGCGTCGTT |
| TOP1-locus | CAGTGAGCCCAAATGCGAAC | GAGACTCCAGAAACGGCTGA |
| CTCF-locus | TTCCCTTATCAGCACCCGC | GCACGGTTTAATCGCTCCAC |
| HPRT-locus | TGGGAAGAGAAGGTGGAAATG | CGCCATCATTCCCGAATCT |
| Inter-ori -MST1P2 | TGTCTCCTACCCATACTTGGA | GAAGCTGTCCACTAGGACTAAAT |
| Inter-ori -PIK3CB | CGGCCTTTCCTAACTCTCTTTG | GAATCTCTCGGGAGGAGGTTAT |
| Primers for luciferase reporter construction（Luc-MCMs/ORC1 wild） | | |
| Luc-MCM2 | CGACGCGTAAGTGCCTGGCTAAGAGGGTTGCGA | CGCGGATCCTGGCGACCAGAAG GGACACGG |
| Luc-MCM4 | CGACGCGTAGGCGCTCGCCACCATGCCCAGTTA | GCTCTAGACACCGCCCCCGACCGCGTTTGTCGT |
| Luc-MCM5 | CGACGCGTAGTGTGTTAGGGTGCGAGAACC | GCTCTAGAGTGGGAACAAACGGCCAGAACAGG |
| Luc-MCM10 | CGACGCGTCATTAGGACACAGAAAAAAGTCAAC | GCTCTAGACAGATGCCCAGATGAGCAATGTAGA |
| Luc-ORC1 | CGACGCGTTTCTAAACCGCCAAAATTAAACCTC | GCTCTAGAAGAAATGAAAAGAAGGTGGAAGGAG |
| **Primers for DRIP-qPCR** | | |
| EGR1 loci | TTCGGATTCCCGCAGTGT | TCACTTTCCCCCCTTTATCCA |
| MCM4 loci | CTTTGGCCCGAATCAACATG | CCTCGCTTGTTTTATCTGCCTCT |
